# Synthesis and biological evaluation of dihydropyrano-[2,3-c]pyrazoles as a new class of PPARγ partial agonists

**DOI:** 10.1371/journal.pone.0162642

**Published:** 2017-02-28

**Authors:** Katrine Qvortrup, Jakob F. Jensen, Mikael S. Sørensen, Irene Kouskoumvekaki, Rasmus K. Petersen, Olivier Taboureau, Karsten Kristiansen, Thomas E. Nielsen

**Affiliations:** 1 Department of Chemistry, Technical University of Denmark, Lyngby, Denmark; 2 Center for Biological Sequence Analysis, Department of Systems Biology, Technical University of Denmark, Lyngby, Denmark; 3 Department of Biology, University of Copenhagen, Ole Maaløes Vej 5, Copenhagen, Denmark; 4 Molécules Thérapeutiques in silico (MTi), Inserm UMR-S 973—Université Paris Diderot, Bat Lamarck A, 35 Rue Hélène Brion, Paris, France; 5 Singapore Centre on Environmental Life Sciences Engineering, Nanyang Technological University, Singapore, Singapore; Universita degli Studi di Bologna, ITALY

## Abstract

Peroxisome proliferator-activated receptor γ (PPARγ) is a well-known target for thiazolidinedione antidiabetic drugs. In this paper, we present the synthesis and biological evaluation of a series of dihydropyrano[2,3-*c*]pyrazole derivatives as a novel family of PPARγ partial agonists. Two analogues were found to display high affinity for PPARγ with potencies in the micro molar range. Both of these hits were selective against PPARγ, since no activity was measured when tested against PPARα, PPARδ and RXRα. In addition, a novel modelling approach based on multiple individual flexible alignments was developed for the identification of ligand binding interactions in PPARγ. In combination with cell-based transactivation experiments, the flexible alignment model provides an excellent analytical tool to evaluate and visualize the effect of ligand chemical structure with respect to receptor binding mode and biological activity.

## Introduction

**Fig 1:**
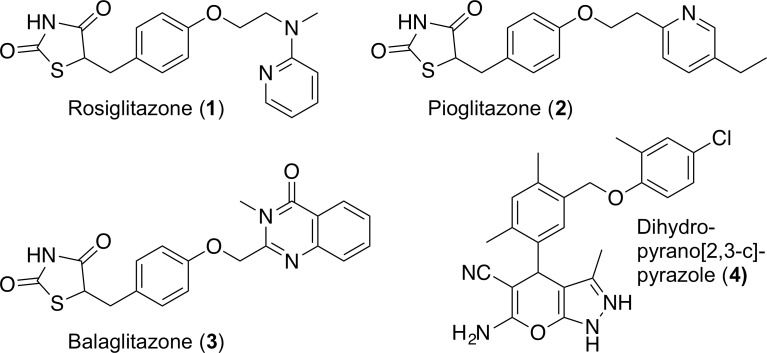
Pharmacologically relevant PPARγ ligands.

Peroxisome proliferator-activated receptors (PPARs) are ligand-activated transcription factors of the nuclear hormone receptor superfamily [1]. Many cellular and systemic roles have been attributed to these receptors, reaching far beyond the stimulation of peroxisome proliferation in rodents after which they were initially named. The three PPAR subtypes PPARα, PPARδ and PPARγ exhibit broad, subtype-specific tissue expression patterns. The PPARγ subtype is most highly expressed in adipose tissue, and agonists for this receptor, increase adipocyte differentiation, enhance insulin sensitivity and improve lipid profiles [2,3]. A variety of synthetic agonists for PPARγ have been studied in the area of antidiabetic drug discovery (Fig 1), such as the full agonists thiazolidinediones (TZDs), including rosiglitazone (**1**) and pioglitazone (**2**). These drugs are now well established in clinical practice for the treatment of type II diabetes. However, despite their effectiveness in lowering blood glucose levels and improving insulin sensitivity [4], they have recently been associated with severe side effects, such as fluid retention, weight gain, cardiac hypertrophy, and hepatotoxicity [5,6]. Strong evidence suggest that these side-effects may be associated with full agonism, and much recent focus has been given to the development of partial agonists, which are expected to retain full insulin sensitizing effects with minimal side-effects [5,6]. One such partial agonist, balaglitazone (**3**), was shown to activate PPARγ and improve glucose levels without causing undesirable accumulation of fluid and fat [7].

In view of these developments, the discovery of novel selective PPARγ agonists with partial binding properties would be advantageous not only as candidates for the treatment of type II diabetes, but also as chemical probes to investigate the function of PPARγ, as new roles for it are currently being identified, e.g. as anti-obesity drug target [8].

Recently, we reported [9] an integrated *in silico* / *in vitro* workflow, based on pharmacophore- and structure-based virtual screening of the ZINC library, coupled with competitive binding and transactivation assays, and adipocyte differentiation and gene expression studies. In this study, dihydropyrano[2,3-*c*]pyrazole **4** was identified as a promising PPARγ partial agonist combining high inhibitor activity (IC_50_ = 0.03 mM) with a relatively poor induction of adipocyte differentiation. This promoted us to further investigate the dihydropyrano[2,3-*c*]pyrazoles as a novel class of PPARγ ligands. Herein, we present the development of a synthetically tractable scale-up synthesis of dihydropyrano[2,3-*c*]pyrazole **4** for further biological studies. Furthermore, we describe the synthesis and biological evaluation of a series of novel dihydropyrano[2,3-*c*]pyrazole derivatives with high structural variation.

## Results and discussion

### 1. Chemistry

#### 1.1 Dihydropyrano[2,3-c]pyrazoles

Dihydropyrano[2,3-*c*]pyrazoles constitute an important class of compounds with a wide range of biological properties, such as anticancer [10], antimicrobial [11] and anti-inflammatory [12] activities. Only recently, we published data that identified the dihydropyrano[2,3-*c*]pyrazole **4** as a promissing PPARγ partial agonist for the treatment of type II diabetes.

**Fig 2:**
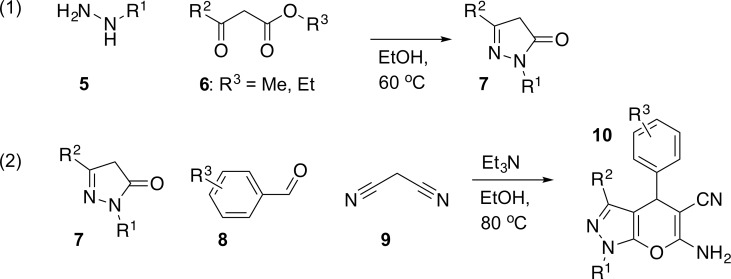
Reaction pathway for the synthesis of dihydropyrano[2,3-*c*]pyrazole derivatives 10.

The most common and convenient approach towards diverse dihydropyrano[2,3-*c*]pyrazoles is a two-step procedure (Fig 2) comprising the following steps: (1) Condensation of hydrazine derivative **5** is condensensed with β-keto ester **6** under basic conditions to produce the 1*H*-pyrazol-5(4*H*)-one derivative **7**; (2) Three-component base-catalyzed reaction of 1*H*-pyrazol-5(4*H*)-one **7**, aromatic aldehyde **8** and malononitrile **9**. The three-component reaction involve a tandem Michael addition-Thorpe-Ziegler-type reaction, followed by tautomerization [13] to generate the dihydropyrano[2,3-*c*]pyrazole core **10**. It should be noted that these compounds may exist in the 1,4-dihydro or 2,4-dihydro tautomeric forms when the N1 position is unsubstituted (R^1^ = H).

The presented protocol represents a synthetically tractable strategy for drug discovery as the target molecule is synthesized in two high-yielding steps without the need for time-consuming chromatography. Furthermore, the general two-step sequence (Fig 2) is easily amenable to the preparation of combinatorial libraries of diverse dihydropyrano[2,3-c]pyrazoles. Given the large number of commercially available aldehydes and the easy access to hydrazines and β-keto esters, this method provides access to dihydropyrano[2,3-c]pyrazole libraries with high appendage diversity introduced through substituents at the 1-, 3-, and 4-positions.

#### 1.2 Scale-up synthesis of lead compound 4

Dihydropyrano[2,3-*c*]pyrazole **4** is a promissing PPARγ partial agonist, that may serve as an entry for the development of new antidiabetics. We initiated our studies by developing a practical and gram-scale synthesis of **4** for compound validation, further medicinal chemistry evaluation, and lead optimization studies.

Considering the synthetic strategy outlined in Fig 2, aldehyde building block **15** constitutes a key intermediate for the scale-up synthesis of **4**. The readily available bromomethylbenzaldehyde **14** was identified as the most readily available precursor to aldehyde **15**. Our stepwise synthesis of **14** commenced with *m*-xylene, which was bis-chloromethylated to afford 1,5-bis(chloromethyl)-2,4-dimethylbenzene **12** [14]. This was then converted to dialdehyde **13** in a Sommelet reaction, following a procedure developed by Wood [15]. Selective reduction of one of the aldehyde moieties with NaBH_4_ and subsequent treatment of the resulting monoalcohol with HBr in boiling AcOH afforded the bromomethylbenzaldehyde **14** [16]. Compound **14** was quantitatively converted to aldehyde **15** by reaction with 4-chloro-2-methylphenol in acetonitrile at 50°C in the presence of K_2_CO_3_.

**Fig 3:**
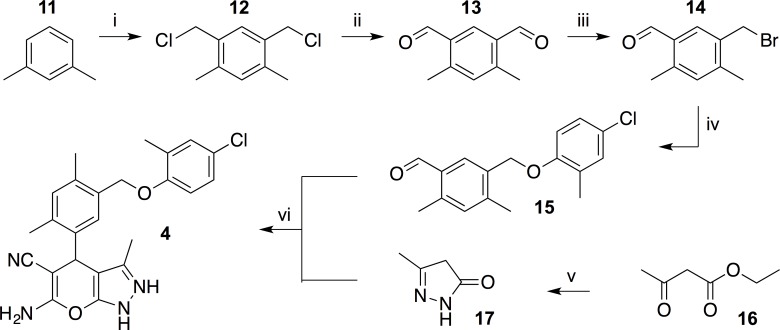
Reaction pathway for the synthesis of dihydropyrano[2,3-*c*]pyrazole 4. (i) Parafomaldehyde, AcOH, conc. HCl (aq), 70°C, 48 h (37%); (ii) hexamethylenetetramine, EtOH, H_2_O, reflux, 16 h (37%); (iii) (a) NaBH_4_, EtOH, (b) HBr, AcOH; (iv) 4-chloro-2-methylphenol. K_2_CO_3_, CH_3_CN, 50°C, 3 h (99%); (v) hydrazine hydrate, EtOH, 0°C to 60°C, 3 h (90%); (vi) malonitrile, Et_3_N, EtOH, 80°C, 17 min (72%).

With substantial amounts of key intermediate **15** in hand, we investigated the final tandem Michael addition-Thorpe-Ziegler reaction (Fig 2) to generate the desired dihydropyrano[2,3-*c*]pyrazole. Reactant **17** [17] was easily available from ethyl acetoacetate and hydrazine hydrate (90% yield after re-crystallization). Finally, treatment of **17** with aldehyde **14** and malononitrile in EtOH resulted in precipitation of **4** [18] directly from the reaction mixture as a pure, white powder in 72% yield (Fig 3).

#### 1.3 Synthesis of 1,4-dihydropyrano-[2,3-c]pyrazoles

**Fig 4:**
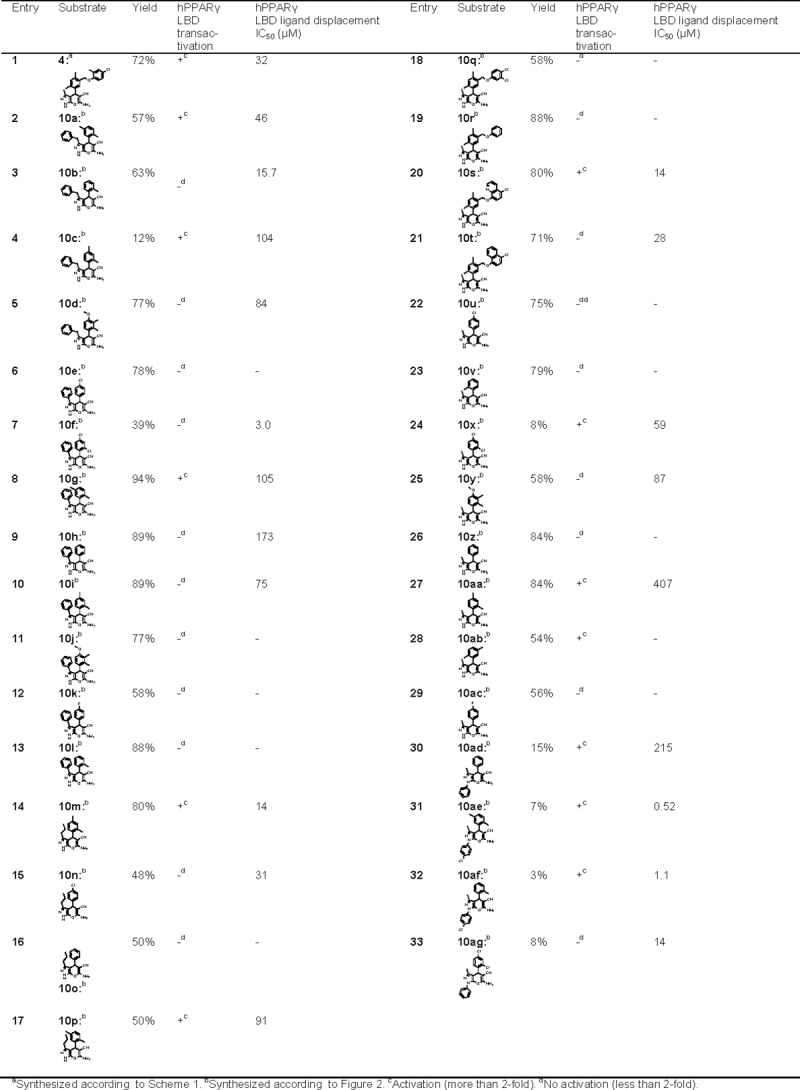
Chemical and biological data for dihydropyrano[2,3-*c*]pyrazoles.

Next, the two-step protocol was applied to prepare a collection of 1,4-dihydropyrano[2,3-c]pyrazoles. All starting hydrazines were purchased from commercial suppliers. The corresponding β-keto esters were synthesized either according to Yuasa and Tsuruta [19] or by deprotonation of esters and subsequent reaction with ethyl acetate [20]. Aldehydes were all purchased or prepared in one step from **14** according to the procedure described for the synthesis of **15**. Following the general procedure (Fig 2), a library of 32 structurally diverse 1,4-dihydropyrano[2,3-c]pyrazoles were synthesized (Fig 4). The ease of this procedure should be emphasized. The reaction was performed at 80°C for 2 h and upon cooling to room temperature, nearly all products precipitated as discrete powders with no need for further purification.

### 2. Biological evaluation

#### 2.1 Competitive binding and transactivation assays

The dihydropyrano[2,3-*c*]pyrazoles **4**, **10a**-**10ag** were tested for transactivation and affinity from competitive binding assay (Fig 4). Twenty-three of the dihydropyrano[2,3-*c*]pyrazoles dose-dependently displaced a labeled PPARγ ligand *in vitro* in a TR-FRET competitive binding assay. Potency of binding as compared with known PPAR ligands was ranging from low affinity, as for compound **10c**, **10g**, **10h**, **10aa** and **10ad** with IC_50_ > 100 μM, to fairly high affinity, as observed for compound **10ae** and **10af** (IC_50_ ≤ 1 μM) (Fig 4). Rosiglitazone was measured to displace with an IC_50_ of 58 nM in this assay, in agreement with previously reported affinities [21]. Further, to examine receptor binding at a more functional level, we analysed the ability of the compounds to induce transcriptional activation in a cell based transactivation assay. Fourteen compounds were found to stimulate PPARγ-mediated transactivation (Fig 4). As several relatively potent binders (compounds **10b**, **10f** and **10ag**) did not induce transcriptional activation, we speculated whether these compounds were behaving as antagonists. However, both cellular uptake and compound “metabolism” could influence the actual concentration, and it cannot be excluded that some of the compounds did not reach saturating concentrations in the cell-based transactivation assay.

It was indeed observed that most of the tested dihydropyrano[2,3-*c*]pyrazole compounds showed some activity against the PPARγ receptor, with the exception of compounds **10e**, **10j**, **10k**, **10l**, **10z**, **10ab** and **10ac**. In analogy with previous SAR analyses of PPARγ interacting compound series, the resulting SAR is not easily interpreted. However, it appears that the 2,5-dimethylaryl (**10a**, **10g**, **10p**, **10ab**, **10ae**) and the N1 4-Cl-phenyl moieties (**10ae**, **10af**) are structural elements that confer transactivation to the dihydropyranozole scaffold. Furthermore, a strong correlation was observed between structure and binding affinity for the compounds, with the two NI 4-chlorophenyl-substituted dihydropyrano[2,3-c]pyrazoles (**10ae** and **10af**) both being identified as high-affinity binders with potency in the micro molar range.

The two most potent hits (**10ae** and **10af**) were further tested for transactivation selectivity towards all three PPAR subtypes. Both of the hits were found to be selective against PPARγ.

#### 2.2 Evaluation of PPARγ binding mode

The binding modes of the dihydropyrano[2,3-*c*]pyrazoles **4**, **10a**-**10ag** were explored with the multiple individual alignment approach [22], [23]. For this purpose, a model set of PPARγ template ligands was generated on the basis of PPARγ agonist-receptor complexes from the Protein Data Bank (PDB). More specifically, the template set was created from twenty-three PDB PPARγ ligands accounting eleven full and twelve partial agonists (see S1 File). Each template was made from an agonist-bound PPARγ structure and removal of the receptor leaving the template agonist in its crystal conformation. Afterwards, individual alignment attempts of a given test molecule on each of the generated templates followed by reintroduction of the receptor and energy calculation, provided a hit list of matching binding modes. According to the targets encoded by these templates, a binding profile for the test ligand was generated.

In order to identify and implement rational threshold values for the docking screen, all twenty-three known PPARγ model ligands were docked with the procedure described above. It was found that all model-ligands could be identified by their own template. To prioritize the best hits and reduce the number of false positives, the internal strain (*dU*), configurational similarity (*dF*) and alignment score (*dS*) constraints were set to a threshold value of 1. Furthermore, only compounds with an Autodock docking score (ΔG) more negative than a threshold value of -10 kcal/mol passed the docking screen, while any remaining compounds were discarded.

To validate the model, a test set of twenty-four known PPARγ ligands, including nine full agonists, seven partial agonists, and eight inactives, was collected from the literature (see S2 File). The set of agonists was constituted by known antidiabetic drugs, including rosiglitazone (**1**, TZD), farglitazar (non-TZD) and other biological relevant agonist with either selective PPARγ or dual PPARα/γ, PPARδ**/**γ selectivity, but no pan PPAR agonists were included. It was ensured that the compounds covered a broad range of activities, from weak to medium and strong agonists. The inactives were manually selected from a set of PPARγ inactive compounds that were structurally similar to the selected PPARγ agonists. Upon evaluation of the test set of PPARγ ligands in the model, seven of the nine full agonists were found to match a full agonist template (with the exception of CID-11384748 and CID-44411945) with four of them matching on more than one template. Six out of seven partial agonists were observed to align on a partial agonist template (with the exception of CID-15069295). Unfortunately, due to the high structural similarity between actives and inactives, the model was not always able to discriminate between the two, leading to several of the inactive test compounds being identified as actives (false positives). However, in combination with cell-based transactivation experiments, the presented model provides a promising analytical tool to evaluate and visualize the effect of ligand chemical structure in respect to receptor binding mode and biological activity.

**Fig 5:**
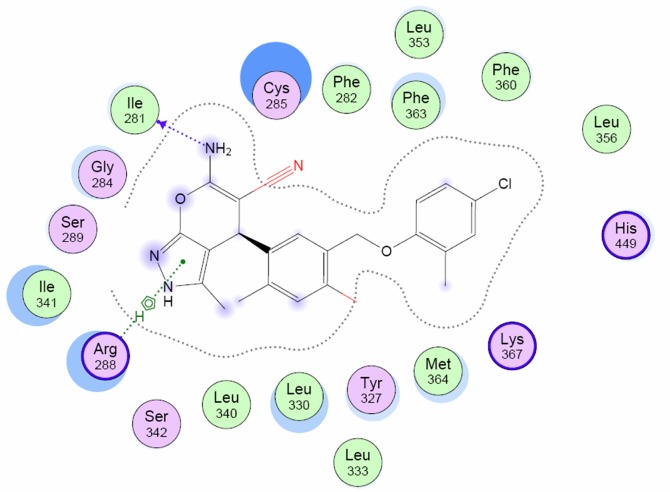
The ligand 4 presented in the aligned binding mode on CID-10229498.

Next, the methodology was applied to the reference dihydropyrano[2,3-c]pyrazole PPARγ partial agonist **4**. The ligand **4** was found to match two of the partial agonist templates (CID-2742752 and CID-10229498). From further inspection of the suggested binding modes, it was concluded that the CID-10229498 binding mode is reasonable (Fig 5). The dihydropyrano[2,3-c]pyrazole group of **4** was observed to make several stabilizing hydrophobic interactions in the β-sheet regions, while the amino group formed a hydrogen bond with Ile281 located in Helix3. Furthermore, the central benzene ring made strong interactions with Cys285.

Finally, we analysed the binding modes of 32 novel potential PPARγ partial agonists (**10a**-**10ag**) by incorporation in twenty-three ligand templates in the individual alignment model. Of the 32 compounds analysed by the model, we were able to identify binding modes for twenty-one of them. All ligands without identifiable binding modes were found to be low affinity binders (IC_50_ < 30 mM) in the ligand displacement experiments.

**Fig 6:**
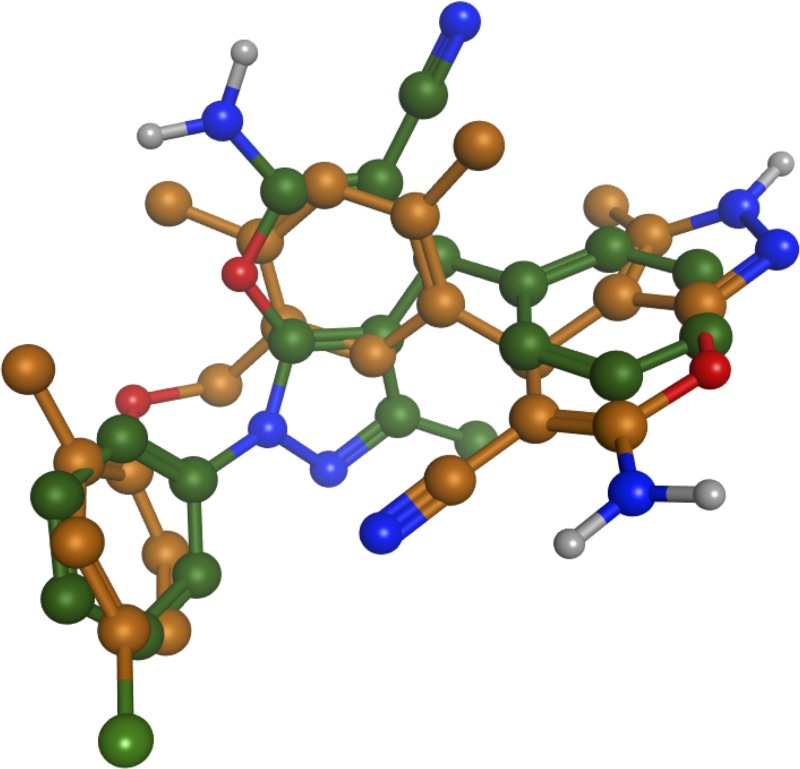
The ligands 4 (orange) and 10ag (green) shown on top of each other from alignment on CID-10229498. The altered ligand structure induces a relocation of the ligand in the receptor, allowing the phenyl-substituent on the dihydropyrano[2,3-c]pyrazole N1 in 10ag to participate in several stabilizing hydrophobic interactions in the β-sheet regions.

Six of the dihydropyrano[2,3-c]pyrazole compounds (**10q**, **10r**, **10s**, **10ae**, **10af** and **10ag**) displaying highest transactivation potential were found to match the same CID-10229498 template as reference dihydropyrano[2,3-c]pyrazole **4**. A closer inspection of the docked ligands revealed that although all of the six ligands matched the same template, they clustered around two distinct binding modes. A strong correlation between binding mode and chemical structure was observed. The ligands **10q**, **10r** and **10s** share high structural similarity with dihydropyrano[2,3-c]pyrazole **4** and were all observed to have identical alignments in the CID-10229498 template displaying the same types of interactions as described for **4**. However, the presence of a substituent on the dihydropyrano[2,3-c]pyrazole N1 clearly influences the receptor ligand complex. The high-affinity ligands **10ae, 10af** and **10ag** have a N1 phenyl-substituent in common. Although they were recognized to match the CID-10229498 template, they seemed to adopt a different but mutually similar scaffold positioning (Fig 6). The altered ligand structure induces a relocation of the ligand in the receptor, allowing the dihydropyranol[2,3-c]pyrazole phenyl-group to participate in several stabilizing hydrophobic interactions with Ser342 and Ile341 in the β-sheet regions. The altered positioning places the pyrano[2,3-c]pyrazole moiety in close proximity to Helix3, where strong interactions with Cys285 are observed. Furthermore, the carbonitrile group may interact with Arg288.

### Conclusions

In here we report dihydropyrano[2,3-*c*]pyrazoles as a novel class of PPARγ partial agonists. A synthetically tractable scale-up synthesis of lead compound dihydropyrano[2,3-*c*]pyrazole **4** has been developed for compound validation as well as further medicinal chemistry efforts. Furthermore, the synthesis and biological evaluation of a series of novel dihydropyrano[2,3-*c*]pyrazole derivatives with high structural variation has been accomplished. Competitive binding and transactivation assay experiments showed that most of the compounds displayed some activity against PPARγ with the two NI 4-chlorophenyl-substituted dihydropyrano[2,3-c]pyrazoles (**10ae** and **10af**) both being identified as selective high-affinity binders with potency in the micro molar range.

Moreover, a novel approach based on multiple individual flexible alignment for the identification of ligand binding interactions in PPARγ was developed. In combination with cell-based transactivation experiments, the flexible alignment model provides an excellent analytical tool to evaluate and visualize the effect of ligand chemical structure in respect to receptor binding mode and biological activity.

## Experimental section

### 1. Materials and measurements

All reagents used were commercially available. All solvents were of HPLC grade. Analytical LC-MS analysis was performed on a Waters AQUITY UPLC system equipped with PDA and SQD MS detector; column: AQUITY UPLC BEH C18 1.7μm, 2.1 x 50mm; column temp: 65°C; solvent A: 0.1% formic acid (aq); solvent B: 0.1% formic acid (acetonitrile); gradient: 5% B to 100% B in 2.4 min, hold for 0.1 min, total run-time ca. 2.6 min. ^1^H and ^13^C NMR 300 MHz spectra were recorded on a Varian Mercury 300 BB spectrometer at room temperature. All NMR spectra were recorded using CDCl_3_ or DMSO-*d*_6_ as solvents

### 2. Synthesis

#### 2.1 1,3-Bis(chloromethyl)-4,6-dimethylbenzene (12) [15]

A stirred solution of *m*-xylene (100.0 mL, 0.818 mol), paraformaldehyde (58.0 g, 1.93 mol), acetic acid (99%, 200 mL) and conc. HCl (aq, 800 mL) was mechanical stirred for 48 h at 70°C. During the last part of the reaction time (18 h), the product precipitated as a white solid. The reaction mixture was cooled to room temperature, and the product isolated by filtration. The filter cake was washed with water and then dissolved in boiling heptane (200 mL). The boiling heptane phase was washed with hot 10% aqueous NaHCO_3_ (50 mL) and then phase separated. Upon cooling to room temparature, the product precipitated as a white solid. Yield: 60.3 g (37%). ^1^H NMR (300 MHz, CDCl_3_): δ = 7.26 (s, 1H, Ar**H**), 7.06 (s, 1H, Ar**H**), 4.58 (s, 4H, C**H**_2_Cl), 2.40 (s, 6H, 2 × ArC**H**_3_); ^13^C NMR (75 MHz, CDCl_3_): δ = 138.09, 133.61, 133.38, 131.33, 44.50, 18.54.

#### 2.2 4,6-Dimethylisophthalaldehydebenzene (13) [16]

A stirred suspension of 1,3-bis(chloromethyl)-4,6-dimethylbenzene (**12**) (80.0 g, 0.394 mol), hexamethylenetetramine (110.5 g, 0.789 mol), ethanol (1000 mL) and water (500 mL) was refluxed for 16 h. The hot reaction mixture was filtered and the filtrate cooled to room temperature. The pH was adjusted to 1–2 with 4.0 M HCl (aq) and extracted with CH_2_Cl_2_ (3 × 700 mL). The combined organic phase was dried with Na_2_SO_4_, filtered and solution concentrated to around 100 mL under reduced pressure. This resulted in precipitation of 4,6-dimethylisophthal-aldehydebenzene as a white solid, which was isolated by filtration. Yield: 23.3 g (37%). ^1^H NMR (300 MHz, CDCl_3_): δ = 10.25 (d, J = 0.6 Hz, 2H, 2 × C**H**O), 8.22 (s, 1H, Ar**H**), 7.18 (s, 1H, Ar**H**), 2.70 (d, J = 0.9 Hz, 6H, 2 × ArC**H**_3_); ^13^C NMR (75 MHz, CDCl_3_): δ = 191.71, 146.38, 136.94, 135.82, 132.78, 20.18; UPLC-MS (ESI): *m/z* = 163.1 (M+H)^+^.

#### 2.3 5-Bromomethyl-2,4-dimethylbenzaldehyde (14)

4,6-Dimethylisophthalaldehyde (23.3 g, 0.144 mol) was dissolved in EtOH (500 mL) with vigorous stirring at room temperature. The reaction mixture was then treated with NaBH_4_ (0.55 g, 0.0146 mol) in one portion. After stirring for 2 h at room temperature, the solution was treated with another portion of NaBH_4_ (0.55 g, 0.0146 mol). After additional 2 h of stirring, the reaction mixture was treated with a third portion of NaBH_4_ (0.38 g, 0.010 mol) and the reaction mixture was left with stirring overnight at room temperature. EtOH was removed under reduced pressure and the remaining oil was treated with boiling water (200 mL) and the mixture cooled to room temperature. Acidification to pH 1–2 with 4.0 M HCl (aq) and treatment with brine (200 mL) and EtOAc (300 mL) afforded a two-phase system, which was separated. The water phase was extracted with EtOAc (2 × 200 mL). The combined organic phase was washed with brine (200 mL), dried over Na_2_SO_4_, filtered and concentrated in *vacuo*. The resulting yellow oil was purified by flash column chromatography on silica gel (eluent: heptane/EtOAc, 95:5) to afford 7.5 g (23%) of 5-bromomethyl-2,4-dimethyl-benzaldehyde (**14**) as a white solid and 6.0 g (26%) of recovered 4,6-dimethylisophthalaldehyde (**13**). ^1^H NMR (300 MHz, CDCl_3_): δ = 10.20 (s, 1H, C**H**O), 7.74 (s, 1H, Ar**H**), 7.10 (s, 1H, Ar**H**), 4.53 (s, 2H, C**H**_2_Br), 2.63 (s, 3H, ArC**H**_3_), 2.45 (s, 3H, ArC**H**_3_); ^13^C NMR (75 MHz, CDCl_3_): δ = 191.82, 143.80, 141.37, 134.54, 134.21, 133.46, 132.64, 31.20, 19.27, 19.13; UPLC-MS (ESI): *m/z* = 227.1, 229.1 (M+H)^+^.

#### 2.4 5-((4-Chloro-2-methylphenoxy)methyl)-2,4-dimethylbenz-aldehyde (15) [17]

5-Bromomethyl-2,4-dimethylbenzaldehyde (1.0 g, 4.40 mmol) and 4-chloro-2-methylphenol (657 mg, 4.62 mmol) were dissolved in acetonitrile (50 mL) and treated with K_2_CO_3_ (912 mg, 6.60 mmol). The suspension was heated to 50°C with stirring. After 3 h the precipitate was removed by filtration and the filtrate concentrated *in vacuo*. Purification by flash column chromatography on silica gel (eluent: heptane/EtOAc 95:5) to afford the aldehyde **15** as a white solid. Yield: 1.26 g (99%). ^1^H NMR (300 MHz, CDCl_3_): δ = 10.22 (d, *J* = 0.9 Hz, 1H, C**H**O), 7.84 (s, 1H, Ar**H**), 7.20–7.07 (m, 2H, Ar**H**), 6.83 (dd, *J* = 8.0, 1.1 Hz, 1H, Ar**H**), 5.02 (s, 2H, OC**H**_2_Ar) 2.65 (s, 3H, ArC**H**_3_), 2.40 (s, 3H, ArC**H**_3_), 2.22 (d, *J* = 0.8 Hz, 3H, ArC**H**_3_); ^13^C NMR (75 MHz, CDCl_3_): δ = 192.29, 155.38, 143.27, 140.71, 134.17, 133.27, 132.31, 132.26, 130.71, 129.05, 126.49, 125.59, 112.35, 68.23, 19.31, 19.23, 16.38; UPLC-MS (ESI): *m/z* = 289.2 (M+H)^+^.

#### 2.5 3-Methyl-1H-pyrazol-5(4H)-one (17) [18]

Ethyl acetoacetate (10.0 g, 0.077 mol) was dissolved in EtOH (150 mL) and cooled to 0°C. With stirring the solution was treated dropwise with hydrazine hydrate (3.5 g, 0.070 mol). After complete addition the solution was heated to 60°C for 3 h and the concentrated *in vacuo*. The resulting solid was re-crystallized from Et_2_O/EtOAc/EtOH to give **17** as a light yellow powder. Yield: 6.2 g (90%). ^1^H NMR (300 MHz, DMSO-*d*_d_): δ = 10.40 (bs, 2H, N**H** + O**H** (tautomeric enol form)), 5.21 (s, 1H, C**H** = COH (tautomeric enol form)), 2.08 (s, 3H, ArC**H**_3_); ^13^C NMR (75 MHz, CDCl_3_): δ = 161.22, 139.54, 89.03 (tautomeric enol form), 49.37 (tautomeric keto form), 11.28.

#### 2.6 6-Amino-4-(5-chloro-2-methylphenoxy)methyl)-2,4-dimethylphenyl)-3-methyl-1,4-dihydropyrano[2,3-c]pyrazole-5-carbonitrile (4) [19]

5-((4-chloro-2-methylphenoxy)methyl)-2,4-dimethyl-benzaldehyde (800 mg, 2.77 mmol) and malonitrile (183 mg, 2.77 mmol) was suspended in EtOH (30 mL) and treated with Et_3_N (280 mg, 2.77 mmol). The solution was placed in a pre-heated oil bath (80°C) and stirred for 2 min., followed by addition of 3-methyl-1*H*-pyrazol-5(4*H*)-one **17** (272 mg, 2.77 mmol. The solution was stirred at 80°C for 15 min. Cooling of the reaction to room temperature, resulted in precipitation of **4** as a white solid, which was isolated by filtration. Yield: 862 mg (72%). ^1^H NMR (300 MHz, DMSO-*d*_6_): δ = 12.06 (s, 1H, ArN**H**), 7.21–7.18 (m, 1H, Ar**H**), 7.14 (d, *J* = 8.8 Hz, 1H, Ar**H**), 7.06 (s, 1H, Ar**H**), 7.03–6.96 (m, 2H, Ar**H**), 6.82 (s, 2H, ArN**H**_2_), 4.99 (s, 2H, ArC**H**_**2**_OAr), 4.80 (s, 1H, C**H**Ar), 2.28 (s, 3H, ArC**H**_3_), 2.24 (s, 3H ArC**H**_3_), 2.06 (s, 3H, ArC**H**_3_), 1.63 (s, 3H ArC**H**_3_); ^13^C NMR (75 MHz, DMSO-*d*_6_): δ = 160.76, 155.15, 155.09, 139.44, 135.27, 134.38, 134.26, 132.85, 132.19, 129.91, 128.69, 128.40, 126.30, 123.80, 120.88, 113.32, 97.64, 67.80, 56.99, 49.28, 18.53, 17.94, 15.78, 9.57; UPLC-MS (ESI): *m/z* = 435.3 (M+H)^+^.

#### 2.7 General synthetic procedure for 1H-pyrazol-5(4H)-one compounds

A solution of β-ketoester (0.011 mmol) in ethanol (50 mL) was treated with the appropriate hydrazine (0.011 mmol) at 0°C. The mixture was allowed to reach room temperature and then heated to 60°C for 3 h. The solvent was removed *in vacuo* and the residue purified by recrystallization or column chromatography.

**2.7.1 3-Benzyl-1H-pyrazol-5(4H)-one.** The title compound was synthesized from methyl 3-oxo-phenylbutanoate (2.2 g) and hydrazine hydrate (0.44 g) according to the general procedure. The product was purified by recrystallization from toluene/heptane and isolated as a white/yellow powder. Yield: 0.84 g (42%). M.p.: 190°C. ^1^H NMR (300 MHz, DMSO-*d*_6_): δ = 3.35 (s, 1H), 3.78 (s, 2H), 5.21 (s, 1H), 7.20–7.32 (m, 5H).

#### 2.8 General synthetic procedure for dihydropyrano[2,3-c]pyrazole compounds (10a-10ah)

The appropriate aldehyde (0.0014 mmol) and malononitrile (0.095 g, 0.0014 mmol) was suspended in ethanol (20 ml) and treated with Et_3_N (0.145 g, 0.0014 mmol). The solution was raised into an preheated oil bath (80°C) and stirred for 2 min, followed by the addition of the appropriate 1*H*-pyrazol-5(4*H*)-one (0.0014 mmol). After stirring at 80°C for 15 min, the solution was cooled to room temperature. The resulting precipitate was isolated by filtration and washed ethanol.

**2.8.1 6-Amino-3-benzyl)-4-(2,5-dimethylphenyl)-1,4-dihydropyrano[2,3-c]pyrazole-5-carbonitrile (10a).** The title compound was synthesized from 2,5-dimethylbenzaldehyde (0.188 g) and 3-benzyl-1*H*-pyrazol-5(4*H*)-one (0.244 g) according to the general procedure. The product was isolated as a white powder. Yield: 0.512 g (57%). ^1^H NMR (300 MHz, DMSO-*d*_6_): δ = 12.33 (s, 1H, ArN**H**), 7.13 (s, 3H, 3 × Ar**H**), 7.04–6.79 (m, 4H, 4 × Ar**H**), 6.80–6.63 (m, 3H, Ar**H +** ArN**H**_**2**_), 4.70 (s, 1H, C**H**Ar), 3.56 (d, *J* = 15.6 Hz, 1H, ArC**H**H), 3.27 (d, *J* = 15.7 Hz, 1H, ArC**H**H), 2.15 (s, 3H, ArC**H**_3_), 2.11 (s, 3H, ArC**H**_3_). ^13^C NMR (75 MHz, DMSO-*d*_d_): δ = 160.53, 155.23, 138.08, 137.34, 135.14, 132.02, 130.52, 129.50, 129.43, 128.31, 127.98, 127.43, 126.28, 120.69, 97.59, 56.90, 49.38, 30.36, 20.68, 18.42. IR (neat): 1486, 1597, 1632, 2194, 3088, 3243, 3473. UPLC-MS (ESI): *m/z* = 357.2 (M+H)^+^.

**2.8.2 6-Amino-3-benzyl-5(o-tolyl)-1,4-dihydropyrano[2,3-c]pyrazole-5-carbonitrile (10b).** The title compound was synthesized from 2-methyl-benzaldehyde (0.172 g) and 3-benzyl-1*H*-pyrazol-5(4*H*)-one (0.244 g) according to the general procedure. The product was isolated as a white powder. Yield: 0.312 g (63%). M.p.: 211–213°C. ^1^H NMR (300 MHz, DMSO-*d*_6_): δ = 12.34 (s, 1H, ArNH), 7.93–6.15 (m, 11H, 9 × ArH + ArNH_2_), 4.74 (s, 1H, CHAr), 3.58 (d, *J* = 16.1 Hz, 1H, ArCHH), 3.24 (d, *J* = 16.2 Hz, 1H, ArCHH), 2.12 (s, 3H, ArCH_3_). ^13^C NMR (75 MHz, DMSO-*d*_6_): δ = 160.59, 155.23, 141.95, 138.07, 137.34, 135.12, 130.62, 129.07, 128.38, 127.99, 126.73, 126.37, 126.31, 120.66, 97.54, 56.76, 56.09, 30.35, 18.77. IR (neat): 1208, 1387, 1640, 2201, 3218, 3312, 3439. UPLC-MS (ESI): *m/z* = 343.2 (M+H)^+^.

***2*.*8*.*3* 6-Amino-3-benzyl-4-(2,4-dimethylphenyl)-1,4-dihydropyrano[2,3-c]pyrazole-5-carbonitrile (10c)** [24]. The title compound was synthesized from 2,4-dimethylbenzaldehyde (0.188 g) and 3-benzyl-1*H*-pyrazol-5(4*H*)-one (0.244 g) according to the general procedure. The product was isolated as a white powder. Yield: 0.312 g (63%). M.p.: 211–213°C. ^1^H NMR (300 MHz, DMSO-*d*_6_): δ = 12.33 (s, 1H, ArN**H**), 7.23–7.10 (m, 3H, Ar**H**), 7.01–6.81 (m, 4H, Ar**H**), 6.78–6.65 (m, 3H, Ar**H** + ArN**H**_**2**_), 4.70 (s, 1H, C**H**Ar), 3.56 (d, *J* = 15.6 Hz, 1H, ArC**H**H), 3.27 (d, *J* = 15.7 Hz, 1H, ArC**H**H), 2.15 (s, 3H, ArC**H**_3_), 2.11 (s, 3H, ArC**H**_3_). ^13^C NMR (75 MHz, DMSO-*d*_6_): δ = 160.49, 155.20, 138.94, 138.05, 137.37, 135.64, 134.87, 131.26, 129.05, 128.33, 127.99, 126.95, 126.24, 120.67, 97.63, 56.94, 50.43, 30.33, 20.58, 18.69. IR (neat): 1208, 1387, 1640, 2201, 3218, 3312, 3439. UPLC-MS (ESI): *m/z* = 357.2 (M+H)^+^.

**2.8.4 6-Amino-3-benzyl)-4-(4-methoxy-2,3-dimethylphenyl)-1,4-dihydropyrano[2,3-c]pyrazole-5-carbonitrile (10d)** [25]. The title compound was synthesized from 2,3-dimethyl-4-methoxybenzaldehyde (0.236 g) and 3-benzyl-1*H*-pyrazol-5(4*H*)-one (0.244 g) according to the general procedure. The product was isolated as a white powder. Yield: 0.428 g (77%). ^1^H NMR (300 MHz, DMSO-*d*_6_): δ 12.30 (s, 1H, ArN**H**), 7.18–7.08 (m, 3H, Ar**H**), 6.86–6.69 (m, 6H, 4 (Ar**H** + ArN**H**_2_), 4.74 (s, 1H, C**H**Ar), 3.73 (s, 3H, ArOC**H**_3_), 3.55 (d, *J* = 15.7 Hz, 1H, ArC**H**H), 3.26 (d, *J* = 15.7 Hz, 1H, ArC**H**H), 2.03 (s, 6H, 2 × ArC**H**_3_). ^13^C NMR (101 MHz, DMSO-*d*_*6*_) δ = 160.3, 155.7, 155.1, 138.0, 137.4, 137.3, 135.0, 128.1, 127.9, 126.1, 120.7, 108.2, 98.3, 58.0, 55.3, 30.3, 14.7, 11.9; IR (neat): 1103, 1399, 2192, 3080, 3236, 3482. UPLC-MS (ESI): *m/z* = 387.2 (M+H)^+^.

**2.8.5 6-Amino-4-(4-chlorophenyl)-3-phenyl-1,4-dihydropyrano[2,3-c]pyrazole-5-carbonitrile** (**10e)** [26]. The title compound was synthesized from 4-chlorobenzaldehyde (0.197 g) and 3-phenyl-1*H*-pyrazol-5(4*H*)-one (0.224 g) according to the general procedure. The product was isolated as a white powder. Yield: 0.381 g (78%). ^1^H NMR (300 MHz, DMSO-*d*_6_): δ = ^1^H-NMR (400 MHz, DMSO-*d*_6_): δ = 12.93 (s, 1H, ArN**H**), 7.50–7.43 (m, 2H, Ar**H**), 7.37–7.23 (m, 5H, 5 (Ar**H**), 7.17–7.09 (m, 2H, 2 (Ar**H**), 6.98 (s, 2H, ArN**H**_2_), 5.06 (s, 1H, C**H**Ar); ^13^C-NMR (100 MHz, DMSO-*d*_*6*_): δ = 160.56, 156.35, 144.04, 138.47, 131.54, 129.68, 129.07, 128.93, 128.87, 128.74, 126.69, 120.92, 97.40, 58.24, 36.48. UPLC-MS (ESI): m/z = 349.1 (M+H)^+^.

**2.8.6 6-Amino)-4-(2,4-dichlorophenyl)-3-methyl-1-phenyl-1,4-dihydropyrano[2,3-c]pyrazole-5-carbonitrile** (**10f).** The title compound was synthesized from from 2,4-dichlorobenzaldehyde (0.243 g) and 3-phenyl-1*H*-pyrazol-5(4*H*)-one (0.224 g) according to the general procedure. The product was isolated as a white powder. Yield: 0.208 g (39%). ^1^H NMR (300 MHz, DMSO-*d*_6_): δ = 5.45 (s, 1H), 7.00–7.50 (m, 10H), 12.92 (s, 1H). ^13^C NMR (75 MHz, DMSO-*d*_d_): δ = 33.67, 49.26, 55.96, 96.14, 120.02, 126.13, 127.80, 128.38, 128.58, 131.98, 132.08, 132.97, 138.05, 140.22, 156.10, 160.58. UPLC-MS (ESI): *m/z* = 383.1 (M+H)^+^.

**2.8.7 6-Amino)-4-(2,5-dimethylphenyl)-3-phenyl-1,4-dihydropyrano[2,3-c]pyrazole-5-carbonitrile (10g)** [25]. The title compound was synthesized from from 2,5-dimethylbenzaldehyde (0.243 g) and 3-phenyl-1*H*-pyrazol-5(4*H*)-one (0.224 g) according to the general procedure. The product was isolated as a white powder. Yield: 0.208 g (39%). ^1^H NMR (300 MHz, DMSO-*d*_6_): δ = 5.45 (s, 1H), 7.00–7.50 (m, 10H), 12.92 (s, 1H). ^13^C NMR (75 MHz, DMSO-*d*_d_): δ = 33.67, 49.26, 55.96, 96.14, 120.02, 126.13, 127.80, 128.38, 128.58, 131.98, 132.08, 132.97, 138.05, 140.22, 156.10, 160.58. UPLC-MS (ESI): *m/z* = 343.2 (M+H)^+^.

**2.8.8 6-Amino-3,4-diphenyl-1,4-dihydropyrano[2,3-c]pyrazole-5-carbonitrile (10h)** [27]. The title compound was synthesized from from benzaldehyde (0.149 g) and 3-phenyl-1*H*-pyrazol-5(4*H*)-one (0.224 g) according to the general procedure. The product was isolated as a white powder. Yield: 0.392 g (89%). ^1^H NMR (300 MHz, DMSO-*d*_6_): δ = 12.84 (s, 1H, ArN**H**), 7.44 (d, *J* = 7.1 Hz, 2H, Ar**H**), 7.33–7.24 (m, 3H, Ar**H**), 7.22–7.17 (m, 2H, Ar**H**), 7.13–7.07 (m, 3H, Ar**H**), 6.93 (s, 2H + ArN**H**_2_), 4.97 (s, 1H, C**H**Ar). ^13^C NMR (75 MHz, DMSO-*d*_6_): δ = 160.05, 156.03, 144.69, 137.87, 128.63, 128.57, 128.34, 128.31, 127.31, 126.63, 126.20, 120.63, 97.42, 58.26, 36.74. UPLC-MS (ESI): *m/z* = 315.1 (M+H)^+^.

**2.8.9 6-Amino-4-(2,4-dimethylphenyl)-3-phenyl-1,4-dihydropyrano[2,3-c]pyrazole-5-carbonitrile (10i).** The title compound was synthesized from from 2,4-dimethylbenzaldehyde (0.243 g) and 3-phenyl-1*H*-pyrazol-5(4*H*)-one (0.224 g) according to the general procedure. The product was isolated as a white powder. Yield: 0.427 g (89%). ^1^H NMR (300 MHz, DMSO-*d*_6_): δ = ^1^H NMR (400 MHz, DMSO-*d*_6_): δ = 12.83 (s, 1H, ArN**H**), 7.64–7.09 (m, 5H, Ar**H**), 6.89–6.81 (m, 4H, 2 (Ar**H** + 2 (ArN**H**_2_), 6.77 (d, *J* = 7.9, 1H, Ar**H**), 5.15 (s, 1H, C**H**Ar), 2.30 (s, 3H, ArC**H**_3_), 2.15 (s, 3H, ArC**H**_3_); ^13^C NMR (100 MHz, DMSO-*d*_6_): δ = 159.98, 156.26, 137.70, 135.27, 134.30, 130.99, 128.79, 128.51 (two overlapping signals), 128.27, 127.01, 126.07 (two overlapping signals), 120.63, 97.78, 57.81, 20.46 (two overlapping signals), 18.81. UPLC-MS (ESI): *m/z* = 343.2 (M+H)^+^.

**2.8.10 6-Amino-4-(4-methoxy-2,3-dimethylphenyl)-3-phenyl-1,4-dihydropyrano[2,3-c]pyrazole-5-carbonitrile (10j).** The title compound was synthesized from 2,3-dimethyl-4-methoxybenzaldehyde (0.236 g) and 3-phenyl-1*H*-pyrazol-5(4*H*)-one (0.224 g) according to the general procedure. The product was isolated as a white powder. Yield: 0.401 g (77%). ^1^H NMR (300 MHz, DMSO-*d*_6_): δ = 12.84 (s, 1H, ArN**H**), 7.94–7.01 (m, 5H, Ar**H**), 6.78–6.72 (m, 4H, 2 (Ar**H +** ArN**H**_**2**_), 5.23 (s, 1H, C**H**Ar), 3.65 (s, 3H, ArOC**H**_3_), 2.26 (s, 3H, ArC**H**_3_), 2.05 (s, 3H, ArC**H**_3_). ^13^C NMR (75 MHz, DMSO-*d*_6_): δ = 159.87, 156.29, 155.25, 137.56, 134.50, 128.84, 128.51, 128.26, 126.74, 126.10, 123.54, 120.73, 109.81, 108.46, 98.46, 58.54, 55.17, 15.06, 12.06 (two overlapping signals). UPLC-MS (ESI): *m/z* = 373.2 (M+H)^+^.

**2.8.11 6-Amino-4-(4-fluorophenyl)-3-phenyl-1,4-dihydropyrano[2,3-c]pyrazole-5-carbonitrile (10k)** [26]. The title compound was synthesized from 4-fluorobenzaldehyde (0.174 g) and 3-phenyl-1*H*-pyrazol-5(4*H*)-one (0.224 g) according to the general procedure. The product was isolated as a white powder. Yield: 0.270 g (58%). ^1^H NMR (400 MHz, DMSO-*d*_6_): δ = 12.90 (s, 1H, ArN**H**), 7.48–7.39 (m, 2H, 2 × Ar**H**), 7.34–7.22 (m, 3H, 3 × Ar**H**), 7.17–7.09 (m, 2H, 2 × Ar**H**), 7.06–6.97 (m, 2H, 2× Ar**H**), 6.94 (s, 2H, ArN**H**_**2**_), 5.04 (s, 1H, C**H**Ar); ^13^C NMR (100 MHz, DMSO-*d*_6_): δ = 160.77 (d, *J* = 241 Hz), 160.02, 155.88, 140.80 (d, *J* = 3 Hz), 137.98, 129.20 (d, *J* = 3 Hz), 128.55, 128.52, 128.35, 126.25, 120.52, 115.02 (d, *J* = 21 Hz), 97.27, 58.09, 35.91. UPLC-MS (ESI): *m/z* = 333.1 (M+H)^+^.

**2.8.12 6-Amino-3-phenyl-4-(o-tolyl)-1,4-dihydropyrano[2,3-c]pyrazole-5-carbonitrile (10l).** The title compound was synthesized from 2-methylbenzaldehyde (0.168 g) and 3-phenyl-1*H*-pyrazol-5(4*H*)-one (0.224 g) according to the general procedure. The product was isolated as a white powder. Yield: 0.405 g (88%). ^1^H NMR (300 MHz, DMSO-*d*_6_): δ = 12.84 (s, 1H, ArN**H**), 7.33–7.20 (m, 6H, 5 × Ar**H**), 7.06–6.88 (m, 3H, 3 × Ar**H**), 6.91 (d, *J* = 2.2 Hz, 1H, Ar**H**), 6.88 (s, 2H, ArN**H**_**2**_), 5.19 (s, 1H, C**H**Ar), 2.33 (s, 3H, ArC**H**_3_); ^13^C NMR (100 MHz, DMSO-*d*_6_): δ = 160.09, 156.23, 142.67, 137.84, 134.59, 130.36, 128.75, 128.59, 128.46, 128.30, 126.46, 126.31, 126.14, 120.59, 97.70, 57.55, 33.20, 18.88. UPLC-MS (ESI): *m/z* = 329.1 (M+H)^+^.

**2.8.13 6-Amino-4-(2,4-dimethylphenyl)-3-propyl-1,4-dihydropyrano[2,3-c]pyrazole-5-carbonitrile (10m)** [25]. The title compound was synthesized from 2,4-dimethylbenzaldehyde (0.188 g) and 3-propyl-1*H*-pyrazol-5(4*H*)-one (0.177 g) according to the general procedure. The product was isolated as a white powder. Yield: 0.345 g (80%). ^1^H NMR (300 MHz, DMSO-*d*_6_): δ = 12.07 (s, 1H, ArN**H**), 6.96–6.85 (m, 3H, Ar**H**), 6.80 (s, 2H, ArN**H**_**2**_), 4.83 (s, 1H, C**H**Ar), 2.20 (s, 6H, 2 × ArC**H**_3_), 2.17–1.88 (m, 2H, C**H**_2_), 1.29–0.91 (m, 2H, C**H**_2_), 0.57 (t, *J* = 7.3 Hz, 3H, C**H**_3_). ^13^C NMR (75 MHz, DMSO-*d*_6_): δ = 160.59, 155.00, 139.54, 139.20, 135.51, 134.73, 131.21, 129.03, 126.86, 120.78, 97.20, 56.94, 26.28, 20.98, 20.57, 18.78, 13.36 (two overlapping aliphatic signals). UPLC-MS (ESI): *m/z* = 309.2 (M+H)^+^.

**2.8.14 6-Amino-4-(4-chlorophenyl)-3-propyl-1,4-dihydropyrano[2,3-c]pyrazole-5-carbonitrile (10n).** The title compound was synthesized from 4-chlorobenzaldehyde (0.197 g) and 3-propyl-1*H*-pyrazol-5(4*H*)-one (0.177 g) according to the general procedure. The product was isolated as a white powder. Yield: 0.441 g (48%). ^1^H NMR (300 MHz, DMSO-*d*_6_): δ = 12.15 (s, 1H, ArN**H**), 7.37 (d, J = 8.4 Hz, 2H, 2 × Ar**H**), 7.19 (d, J = 8.4 Hz, 2H, 2 × Ar**H**), 6.91 (s, 2H, ArN**H**_**2**_), 4.63 (s, 1H, C**H**Ar), 2.17 (ddd, *J* = 15.0, 8.5, 6.7 Hz, 1H, ArC**H**HCH_2_), 2.05 (ddd, *J* = 14.7, 8.5, 6.4 Hz, 1H, ArC**H**HCH_2_), 1.33–1.06 (m, 2H, CH_2_C**H**_2_CH_3_), 0.63 (t, *J* = 7.3 Hz, 3H CH_2_C**H**_3_); ^13^C NMR (100 MHz, DMSO-*d*_6_): δ = 160.75, 154.59, 143.91, 139.82, 131.23, 129.41, 128.40, 120.63, 96.90, 56.97, 35.72, 26.19, 20.95, 13.31. UPLC-MS (ESI): *m/z* = 315.1 (M+H)^+^.

**2.8.15 6-Amino-4-phenyl-3-propyl-1,4-dihydropyrano[2,3-c]pyrazole-5-carbonitrile (10o).** The title compound was synthesized from benzaldehyde (0.149 g) and 3-propyl-1*H*-pyrazol-5(4*H*)-one (0.177 g) according to the general procedure. The product was isolated as a white powder. Yield: 0.441 g (48%). ^1^H NMR (300 MHz, DMSO-*d*_6_): δ = 12.10 (s, 1H, ArN**H**), 7.36–7.27 (m, 2H, 2 × Ar**H**), 7.25–7.19 (m, 1H, Ar**H**), 7.18–7.13 (m, 2H, 2 × Ar**H**), 6.85 (s, 2H, ArN**H**_**2**_), 4.58 (s, 1H, C**H**Ar), 2.16 (ddd, *J* = 14.9, 8.6, 6.6 Hz, 1H, ArC**H**HCH_2_), 2.04 (ddd, *J* = 14.7, 8.6, 6.4 Hz, 1H, ArC**H**HCH_2_), 1.30–1.03 (m, 2H, CH_2_C**H**_2_CH_3_), 0.61 (t, *J* = 7.3 Hz, 3H CH_2_C**H**_3_); ^13^C NMR (100 MHz, DMSO-*d*_6_): δ = 160.71, 154.63, 144.87, 139.76, 128.39, 127.52, 126.75, 120.77, 97.33, 57.39, 36.44, 26.21, 20.93, 13.31. UPLC-MS (ESI): *m/z* = 281.1 (M+H)^+^.

**2.8.16 6-Amino-4-(2,5-dimethylphenyl)-3-propyl-1,4-dihydropyrano[2,3-c]pyrazole-5-carbonitrile (10p).** The title compound was synthesized from 2,5-dimethylbenzaldehyde (0.188 g) and 3-propyl-1*H*-pyrazol-5(4*H*)-one (0.177 g) according to the general procedure. The product was isolated as a white powder. Yield: 0.216 g (50%). ^1^H NMR (300 MHz, DMSO-*d*_6_): δ = 12.09 (s, 1H, ArN**H**), 7.35–6.45 (m, 5H, 3 × Ar**H** + ArN**H**_2_), 4.79 (s, 1H, C**H**Ar), 2.20–1.75 (m, 2H, ArCH_2_), 2.18 (s, 6H, 2 × ArCH_3_), 1.39–0.83 (m, 2H, CH_2_C**H**_2_CH_3_), 0.53 (t, *J* = 7.7 Hz, 3H, CH_2_C**H**_3_). ^13^C NMR (75 MHz, DMSO-*d*_6_): δ = 160.65, 155.00, 142.08, 139.57, 135.00, 131.89, 130.50, 129.45, 127.42, 127.35, 120.80, 97.21, 56.84, 26.28, 20.94, 20.66, 18.46, 13.31(two overlapping). UPLC-MS (ESI): *m/z* = 309.2 (M+H)^+^.

**2.8.17 6-Amino-4-(5-((2,3-dichloro-phenoxy)methyl)-2,4-dimethylphenyl)-3-methyl-1,4-dihydropyrano[2,3-c]pyrazole-5-carbonitrile (10q).** The title compound was synthesized from 5-((3,4-dichlorophenoxy)methyl)-2,4-dimethylbenzaldehyde (0.433 g) and 3-methyl-1*H*-pyrazol-5(4*H*)-one (0.137 g) according to the general procedure. The product was isolated as a white powder. Yield: 0.529 g (83%). ^1^H NMR (300 MHz, DMSO-*d*_6_): δ = 12.06 (s, 1H, ArN**H**), 7.51 (m, 1H, Ar**H**), 7.29 (m, 1H, Ar**H**), 7.00 (m, 3H, ArH), 6.83 (m, 2H, ArN**H**_2_), 5.02 (s, 2H, ArC**H**_**2**_OAr), 4.80 (s, 1H, C**H**Ar), 2.23 (s, 6H, 2 × ArC**H**_3_), 1.64 (s, 3H ArC**H**_3_). ^13^C NMR (75 MHz, DMSO-*d*_6_): δ = 160.74, 157.84, 155.06, 135.31, 135.24, 132.54, 132.03, 131.99, 131.55, 130.86, 129.87, 129.83, 122.51, 120.79, 116.77, 115.95, 97.43, 68.93, 56.74, 40.35, 18.56, 18.04 (two overlapping), 9.59. UPLC-MS (ESI): *m/z* = 455.1 (M+H)^+^.

**2.8.18 6-Amino-4-(2,4-dimethyl-(5-(phenoxy)methyl)phenyl)-3-methyl-1,4-dihydropyrano[2,3-c]pyrazole-5-carbonitrile (10r).** The title compound was synthesized from 2,4-dimethyl-5-phenoxymethyl)-benzaldehyde (0.336 g) and 3-methyl-1*H*-pyrazol-5(4*H*)-one (0.137 g) according to the general procedure. The product was isolated as a white powder. Yield: 0.400 g (74%). ^1^H NMR (300 MHz, DMSO-*d*_6_): δ = 12.06 (s, 1H, ArN**H**), 7.38–7.19 (m, 3H, Ar**H**), 7.14–6.87 (m, 4H, Ar**H**), 6.83 (s, 2H, ArN**H**_2_), 4.97 (s, 2H, ArC**H**_**2**_OAr), 4.81 (s, 1H, C**H**Ar), 2.25 (s, 6H 2 × ArCH_3_), 1.64 (s, 3H ArCH_3_). ^13^C NMR (75 MHz, DMSO-*d*_6_): δ = 160.74, 158.38, 155.07, 139.21, 135.34, 135.03, 134.83, 132.75, 132.44, 129.72, 129.41, 120.81, 120.67, 114.86, 97.38, 68.04, 56.80, 18.55, 18.04 (two overlapping), 9.62. UPLC-MS (ESI): *m/z* = 387.2 (M+H)^+^.

**2.8.19 6-Amino-4-(5-(((5-chloroquinolin-8-yl)oxy)methyl)-2,4-dimethylphenyl)-3-methyl-1,4-dihydropyrano[2,3-c]pyrazole-5-carbonitrile (10s).** The title compound was synthesized from 5-(((5-chloroquinolin-8-yl)oxy)methyl)-2,4-dimethylbenzaldehyde (0.456 g) and 3-methyl-1*H*-pyrazol-5(4*H*)-one (0.137 g) according to the general procedure. The product was isolated as a white powder. Yield: 0.297 g (63%). ^1^H NMR (300 MHz, DMSO-*d*_6_): δ = 12.04 (s, 1H, ArN**H**), 9.05–8.82 (m, 1H, Ar**H**), 8.57–8.38 (m, 1H, Ar**H**), 7.80–7.53 (m, 2H, Ar**H**), 7.28 (m, 1H, Ar**H**), 7.14 (s, 1H, Ar**H**), 7.01 (s, 1H, Ar**H**), 6.83 (s, 2H, ArN**H**_2_), 5.19 (s, 2H, ArC**H**_**2**_OAr), 4.86 (s, 1H, C**H**Ar), 2.31 (s, 3H ArCH_3_), 2.29 (s, 3H ArCH_3_), 1.70 (s, 3H ArCH_3_). ^13^C NMR (75 MHz, DMSO-*d*_6_): δ = 160.75, 155.06, 153.64, 149.86, 140.49, 135.50, 135.03, 132.50, 132.47, 132.17, 130.13, 130.07, 126.69, 126.16, 123.00, 120.83, 120.77, 110.34, 97.50, 69.23, 56.80, 40.08, 18.60, 18.13, 9.66. UPLC-MS (ESI): *m/z* = 472.2 (M+H)^+^.

**2.8.20 6-Amino-4-(5-(((5-chloronaphthalen-1-yl)oxy)methyl)-2,4-dimethylphenyl)-3-methyl-1,4-dihydropyrano[2,3-c]pyrazole-5-carbonitrile (10t).** The title compound was synthesized from 5-(((5-chloronaphthalen-1-yl)oxy)methyl)-2,4-dimethylbenz-aldehyde (0.455 g) and 3-methyl-1*H*-pyrazol-5(4*H*)-one (0.137 g) according to the general procedure. The product was isolated as a white powder. Yield: 0.468 g (71%). ^1^H NMR (300 MHz, DMSO-*d*_6_): δ = 12.10 (s, 1H, ArN**H**), 8.25–8.01 (m, 2H, Ar**H**), 7.74–7.56 (m, 2H, Ar**H**), 7.58 (d, *J* = 8.3 Hz, 2H, Ar**H**), 7.25 (s, 1H, Ar**H**), 7.07 (d, *J* = 8.4 Hz, 1H, Ar**H**), 7.02 (s, 1H, Ar**H**), 6.88 (s, 2H, ArN**H**_2_), 5.19 (s, 2H, ArC**H**_**2**_OAr), 4.86 (s, 1H, C**H**Ar), 2.31 (s, 3H ArCH_3_), 2.29 (s, 3H ArCH_3_), 1.70 (s, 3H ArCH_3_). ^13^C NMR (75 MHz, DMSO-*d*_6_): δ = 160.81, 155.21, 153.04, 135.31, 134.61, 134.57, 134.38, 132.65, 132.37, 130.44, 127.98, 126.62, 126.25, 126.13, 123.67, 122.08, 121.76, 120.91, 109.35, 106.29, 97.69, 68.25, 56.97, 18.55, 18.02 (two overlapping), 9.66. UPLC-MS (ESI): *m/z* = 471.2 (M+H)^+^.

**2.8.21 6-Amino-4-(4-chlorophenyl)-3-methyl-1,4-dihydropyrano[2,3-c]pyrazole-5-carbonitrile (10u)** [28]. The title compound was synthesized from 4-chlorobenzaldehyde (0.197 g) and 3-methyl-1*H*-pyrazol-5(4*H*)-one (0.137 g) according to the general procedure. The product was isolated as a white powder. Yield: 0.301 g (75%). ^1^H NMR (300 MHz, DMSO-*d*_6_): δ = 12.13 (s, 1H, ArN**H**), 7.38 (d, *J* = 8.4 Hz, 2H, 2 × Ar**H**), 7.19 (d, *J* = 8.5 Hz, 2H, 2 × Ar**H**), 6.92 (s, 2H, ArN**H**_2_), 4.63 (s, 1H, C**H**Ar), 1.79 (s, 3H, ArC**H**_3_); ^13^C NMR (75 MHz, DMSO-*d*_6_): δ = 160.90, 154.69, 143.49, 135.65, 131.21, 129.36, 128.45, 120.64, 97.19, 56.74, 35.55, 9.74. UPLC-MS (ESI): *m/z* = 287.1 (M+H)^+^.

**2.8.22 6-Amino-3-methyl-4-(o-tolyl)-1,4-dihydropyrano[2,3-c]pyrazole-5-carbonitrile (10v)** [29]. The title compound was synthesized from 2-methylbenzaldehyde (0.168 g) and 3-methyl-1*H*-pyrazol-5(4*H*)-one (0.137 g) according to the general procedure. The product was isolated as a white powder. Yield: 0.295 g (79%). ^1^H NMR (300 MHz, DMSO-*d*_6_): δ = 12.07 (s, 1H, ArN**H**), 7.18–7.06 (m, 3H, 3 × Ar**H**), (dd, *J* = 7.7, 2.1 Hz, 1H, Ar**H**), 6.82 (s, 2H, ArN**H**_2_), 4.84 (s, 1H, C**H**Ar), 2.28 (s, 3H, ArC**H**_3_), 1.68 (s, 3H, ArC**H**_3_); ^13^C NMR (100 MHz, DMSO-*d*_6_): δ = 160.74, 155.06, 141.86, 135.30, 134.95, 130.46, 128.88, 126.56, 126.30, 120.69, 97.53, 56.73, 33.07, 18.90, 9.52. UPLC-MS (ESI): *m/z* = 267.1 (M+H)^+^.

**2.8.23 6-Amino-4-(2,4-dichlorophenyl)-3-methyl-1,4-dihydropyrano[2,3-c]pyrazole-5-carbonitrile (10x)** [30]. The title compound was synthesized from 2,4-dichlorobenzaldehyde (0.243 g) and 3-methyl-1*H*-pyrazol-5(4*H*)-one (0.137 g) according to the general procedure. The product was isolated as a white powder. Yield: 0.062 g (8%). ^1^H NMR (300 MHz, DMSO-*d*_6_): δ = 1.78 (s, 3H), 5.06 (s, 1H), 7.02 (s, 2H), 7.21 (d, 1H), 7.42 (d, 1H), 7.58 (s, 1H), 12.17 (s, 1H). ^13^C NMR (75 MHz, DMSO-*d*_d_): δ = 9.60, 33.11, 38.69, 55.24, 96.38, 120.33, 128.08, 128.88, 132.16, 132.87, 135.49, 140.13, 154.93, 161.36. IR (neat): 1410, 1585, 2183, 3101, 3246. UPLC-MS (ESI): *m/z* = 321.0 (M+H)^+^.

**2.8.24 6-Amino-4-(4-methoxy-2,3-dimethylphenyl)-3-methyl-1,4-dihydropyrano[2,3-c]pyrazole-5-carbonitrile (10y).** The title compound was synthesized from 2,5-dimethylbenzaldehyde (0.188 g) and 3-methyl-1*H*-pyrazol-5(4*H*)-one (0.137 g) according to the general procedure. The product was isolated as a white powder. Yield: 0.249 g (58%). ^1^H NMR (300 MHz, DMSO-*d*_6_): δ = 1.69 (s, 3H), 2.09 (s, 3H), 2.10 (s, 3H), 3.22 (s, 3H), 4.85 (s, 1H), 6.75–6.80 (m, 4H), 12.04 (s, 1H). ^13^C NMR (75 MHz, DMSO-*d*_d_): δ = 9.69, 10.06, 11.73, 12.01, 14.97, 16.50, 16.68, 30.75, 55.25, 55.53, 55.57, 57.61, 98.19, 99.82, 108.23, 108.37, 120.85, 123.71, 124.98, 125.09, 125.67, 127.24, 134.24, 134.85, 135.31, 154.98, 155.51, 156.90, 157.88, 160.56, 206.56. IR (neat): 1391, 1596, 2191, 3099, 3221. UPLC-MS (ESI): *m/z* = 311.1 (M+H)^+^.

**2.8.25 6-Amino)-3-methyl-4-phenyl-1,4-dihydropyrano[2,3-c]pyrazole-5-carbonitrile (10z)** [31]. The title compound was synthesized from benzaldehyde (0.149 g) and 3-methyl-1*H*-pyrazol-5(4*H*)-one (0.137 g) according to the general procedure. The product was isolated as a white powder. Yield: 0.297 g (84%). ^1^H NMR (300 MHz, DMSO-*d*_6_): δ = 12.09 (s, 1H, ArN**H**), 7.31 (dd, *J* = 8.0, 6.8 Hz, 2H, Ar**H**), 7.23 (d, *J* = 7.4 Hz, 1H, Ar**H**), 7.20–7.13(m, 2H, Ar**H**), 6.86 (s, 2H, ArN**H**_2_), 4.59 (s, 1H, C**H**Ar), 1.78 (s, 3H ArCH_3_); ^13^C NMR (75 MHz, DMSO-*d*_6_): δ = 160.86, 154.76, 144.45, 135.55, 128.43, 127.46, 126.72, 120.79, 97.64, 57.17, 36.23, 9.74. UPLC-MS (ESI): *m/z* = 253.1 (M+H)^+^.

**2.8.26 6-Amino-4-(2,4-dimethylphenyl)-3-methyl-1,4-dihydropyrano[2,3-c]pyrazole-5-carbonitrile (10aa).** The title compound was synthesized from 2,4-dimethylbenzaldehyde (0.188 g) and 3-methyl-1*H*-pyrazol-5(4*H*)-one (0.137 g) according to the general procedure. The product was isolated as a white powder. Yield: 0.330 g (84%). ^1^H NMR (300 MHz, DMSO-*d*_6_): δ = 12.05 (s, 1H, ArN**H**), 6.93 (d, *J* = 2.3 Hz, 2H, Ar**H**), 6.86 (d, *J* = 8.3 Hz, 1H, Ar**H**), 6.79 (s, 2H, ArN**H**_2_), 4.79 (s, 1H, C**H**Ar), 2.24 (s, 3H ArC**H**_3_), 2.23 (s, 3H ArC**H**_3_), 1.69 (s, 3H ArC**H**_3_); ^13^C NMR (75 MHz, DMSO-*d*_6_): δ = 160.65, 155.05, 135.40, 135.27 (two overlapping signals), 134.72, 131.11, 128.85, 126. UPLC-MS (ESI): *m/z* = 281.1 (M+H)^+^.

**2.8.27 6-Amino-4-(2,5-dimethylphenyl)-3-methyl-1,4-dihydropyrano[2,3-c]pyrazole-5-carbonitrile (10ab)** [32]. The title compound was synthesized from 1,5-dimethylbenzaldehyde (0.188 g) and 3-methyl-1*H*-pyrazol-5(4*H*)-one (0.137 g) according to the general procedure. The product was isolated as a white powder. Yield: 0.212 g (54%). ^1^H NMR (300 MHz, DMSO-*d*_6_): δ = 12.05 (s, 1H, ArN**H**), 7.01 (d, *J* = 7.7 Hz, 1H, Ar**H**), 6.92 (dd, *J* = 7.8, 1.8 Hz, 1H, Ar**H**), 6.81 (s, 2H, ArN**H**_2_), 6.78 (s, 1H, Ar**H**), 4.79 (s, 1H, C**H**Ar), 2.24 (s, 3H ArC**H**_3_), 2.19 (s, 3H ArC**H**_3_), 1.69 (s, 3H ArC**H**_3_); ^13^C NMR (75 MHz, DMSO-*d*_6_): δ = 160.71, 155.05, 135.30, 135.01, 131.86, 130.40, 129.19, 127.31 (two overlapping signals), 120.72, 97.59, 56.87, 20.67 (two overlapping signals), 18.52, 9.56. UPLC-MS (ESI): *m/z* = 281.1 (M+H)^+^.

**2.8.28 6-Amino-4-(4-fluorophenyl)-3-methyl-1,4-dihydropyrano[2,3-c]pyrazole-5-carbonitrile (10ac)** [30]. The title compound was synthesized from 4-fluorobenzaldehyde (0.172 g) and 3-methyl-1*H*-pyrazol-5(4*H*)-one (0.137 g) according to the general procedure. The product was isolated as a white powder. Yield: 0.249 g (58%). ^1^H NMR (300 MHz, DMSO-*d*_6_): δ = 1.79 (s, 3H), 4.64 (s, 1H), 6.92 (s, 2H), 7.10–7.30 (m, 4H), 12.13 (s, 1H). ^13^C NMR (75 MHz, DMSO-*d*_d_): δ = 9.78, 35.47, 38.69, 38.96, 39.24, 45.01, 49.29, 57.07, 97.53, 115.07, 115.36, 120.77, 129.33, 129.43, 135.67, 140.68, 140.72, 154.72, 159.37, 160.85. IR (neat):1395, 1491, 1591, 2198, 3090, 3226. UPLC-MS (ESI): *m/z* = 271.1 (M+H)^+^.

**2.8.29 6-Amino-3-methyl-1,4-diphenyl-1,4-dihydropyrano[2,3-c]pyrazole-5-carbonitrile (10ad)** [31]. The title compound was synthesized from benzaldehyde (0.149 g) and 1-phenyl-3-methyl-1*H*-pyrazol-5(4*H*)-one (0.244 g) according to the general procedure. The product was isolated as a white powder. Yield: 0.069 g (15%). ^1^H NMR (300 MHz, DMSO-*d*_6_): δ = 7.82–7.75 (m, 2H, ArN**H**), 7.49 (dd, *J* = 8.5, 7.4 Hz, 2H, Ar**H**), 7.39–7.29 (m, 4H, Ar**H**), 7.29–7.23 (m, 4H, Ar**H**), 7.20 (s, 2H, ArN**H**_2_), 4.68 (s, 1H, C**H**Ar), 1.78 (s, 3H ArC**H**_3_); ^13^C NMR (75 MHz, DMSO-*d*_6_): δ = 159.41, 145.25, 143.87, 143.60, 137.52, 129.33, 128.52, 127.77, 127.04, 126.16, 120.00, 119.96, 98.63, 58.16, 36.73, 12.57. UPLC-MS (ESI): *m/z* = 329.1 (M+H)^+^.

**2.8.30 6-Amino-1-(4-chlorophenyl)-4-(2,5-dimethylphenyl)-3-methyl-1,4-dihydropyrano[2,3-c]pyrazole-5-carbonitrile (10ae).** The title compound was synthesized from 2,5-dimethylbenzaldehyde (0.188 g) and 1-(4-chlorophenyl)-3-methyl-1*H*-pyrazol-5(4*H*)-one (0.288 g) according to the general procedure. The product was isolated as a white powder. Yield: 0.040 g (7%). M.p.: 166–168°C. ^1^H NMR (300 MHz, DMSO-*d*_6_): δ = 1.69 (s, 3H), 2.19 (s, 3H), 2.31 (s, 3H), 4.88 (s, 1H), 6.87 (s, 1H), 6.94 (d, 1H), 7.05 (d, 1H), 7.20 (s, 2H), 7.52 (d, 2H), 7.83 (d, 2H). ^13^C NMR (75 MHz, DMSO-*d*_d_): δ = 12.42, 18.63, 20.69, 49.30, 57.92, 99.00, 119.96, 121.25, 127.71, 129.28, 130.15, 130.41, 132.07, 135.39, 136.47, 140.88, 144.24, 145.70, 159.28. IR (neat): 1383, 1512, 1663, 2198, 3195, 3317, 3465. IR (neat): 1383, 1512, 1663, 2198, 3195, 3317, 3465. UPLC-MS (ESI): *m/z* = 391.1 (M+H)^+^.

**2.8.31 6-Amino-1-(4-chlorophenyl)-3-methyl-4-(o-tolyl))-1,4-dihydropyrano[2,3-c]pyrazole-5-carbonitrile (10af).** The title compound was synthesized from 2-methylbenzaldehyde (0.172 g) and 1-(4-chlorophenyl)-3-methyl-1*H*-pyrazol-5(4*H*)-one (0.288 g) according to the general procedure. The product was isolated as a white powder. Yield: 0.015 g (3%). M.p.: 178°C. ^1^H NMR (300 MHz, DMSO-*d*_6_): δ = 1.69 (s, 3H), 2.37 (s, 3H), 4.94 (s, 1H), 7.09–7.22 (m, 6H), 7.53 (d, 2H), 7.83 (d, 2H). IR (neat): 1384, 1511, 1662, 2224, 3192, 3332, 3453. UPLC-MS (ESI): *m/z* = 377.1 (M+H)^+^.

**2.8.32 6-Amino-4-(2,4-dichlorophenyl)-3-methyl-1-phenyl-1,4-dihydropyrano[2,3-c]pyrazole-5-carbonitrile (10ag)** [32]. The title compound was synthesized from 2,4-dichlorobenzaldehyde (0.243 g) and 3-methyl-1-phenyl-1*H*-pyrazol-5(4*H*)-one (0.242 g) according to the general procedure. The product was isolated as a white powder. Yield: 0.047 g (8%). ^1^H NMR (300 MHz, DMSO-*d*_6_): δ = 1.78 (s, 3H), 5.15 (s, 1H), 7.24–7.55 (m, 7H), 7.61 (s, 1H), 7.77 (d, 2H). ^13^C NMR (75 MHz, DMSO-*d*_d_): δ = 12.37, 49.15, 49.23, 56.15, 97.31, 119.56, 120.06, 126.31, 128.13, 128.38, 128.98, 129.36, 132.50, 132.56, 133.09, 137.44, 139.28, 144.28, 144.84, 159.95. IR (neat): 1387, 1557, 2198, 3322, 3456. UPLC-MS (ESI): *m/z* = 399.1 (M+H)^+^.

### 3. Competitive binding assays

IC_50_ values for respective compounds were determined by competitive binding using time-resolved fluorescence resonance energy transfer (LanthaScreen, Invitrogen) on a Victor2 microplate reader (PerkinElmer). Briefly, a terbium labeled anti-GST antibody was used to label purified GST-tagged PPARγ-LBD (ligand binding domain). Energy transfer from terbium to the tracer, a fluorescent pan PPAR agonist, enabled read-out of each test compounds’s ability to displace the tracer. RFU values from dose-response curves (triplicate sampling) for test compounds as well as positive controls (rosiglitazone) were then analyzed using GraphPad Prism [33]. An unrestrained sigmoidal (one-binding site) dose-response curve was fitted to each data set by linear regression and allowing for determination of IC_50_ values. All compounds were tested in 10 different concentrations ranging from 3 nM to 500 μM (rosiglitazone from 0.3 nM to 10 μM).

### 4. Nuclear receptor-LBD transactivation

Hepa 1–6 cells were grown in minimum essential medium (MEM) (Gibco) supplemented with 10% fetal calf serum (FCS) and antibiotics (62.5 *μ*g/mL penicillin and 100 *μ*g/mL streptomycin).

Mouse embryo fibroblasts (MEFs) were propagated in Dulbeccos Modified Eagle’s Media (DMEM) supplemented with 10% FCS and antibiotics.

Cells (Hepa 1–6 were used for PPARα whereas MEFs were used for PPARγ, PPARδ and RXRα transfections) were transfected in solution by Metafectene (Biontex) lipofection, essentially according to the manufacturer’s instructions and seeded in media (MEM or DMEM supplemented with 10% fetal calf serum and antibiotics in 96-well dishes at 24000 cells/cm^2^. The transfection plasmid mix included the Gal4-responsive luciferase reporter, the expression vector for the fusion between the Gal4 DNA-binding domain and the nuclear receptor ligand binding domains, and a CMV-Renilla luciferase normalization vector (pRL-CMV, Promega). Six hours after addition of transfection mix to the cells, the media was changed to media (MEM or DMEM) supplemented with vehicle (0.1% DMSO), positive control (1 μM Rosiglitazone, 30 nM GW7647, 1 μM L165041 or 0.2 μM LG1069 for PPARγ, PPARα, PPARδ and RXRα, respectively), or compound. Approx. 18 hours later, cells were harvested and lysates analyzed for Photinus and Renilla luciferase activity by luminometry. All data points were performed in at least triplicate and each sample measured in duplicate. Luminometer raw data was analyzed in Microsoft Excel spreadsheets and presented as column graphs depicting average values of triplicates and including standard deviations.

### 5. Multiple flexible alignment template

Evaluation of the dihydropyrano[2,3-*c*]pyrazoles **4**, **10a**-**10ah** PPARγ binding modes was carried out using a multiple flexible alignment approach. The model was developed, refined and evaluated using the flexible alignment application of the MOE software. The agonist model set was generated using the SVL Batch FlexAlign script, based on a PDB compound set of eleven PPARγ full agonists and twelve seven PPARγ partial aonists. The set of model agonists was composed of known antidiabetic drugs, including Rosiglitazone (TZD) and Farglitazar (non-TZD) and other biological relevant agonist with either selective PPARγ or dual PPAR-α/γ, PPAR-δ/γ selectivity; no pan PPAR agonists were included. The compound set employed in the development of the PPARγ agonist binding model is given in S2 File in the Supporting Information. The binding model was then used for the virtual screening of the library of the dihydropyrano[2,3-*c*]pyrazoles (**4**, **10a**-**10ah**). To be a hit, the test ligand pose had to fit the defined threshold of the binding model (ΔG_intr =_ ΔG–ΔG_torsions_, i.e. the torsion scoring term was excluded) less negative than a threshold value of -10 kcal/mol passed the docking screen, and the remaining compounds were discarded.

## Supporting information

S1 File General Supporting Information. (PDF)

S2 File Model set of compounds for development of binding mode model. (DOCX)

S3 File Test set of compounds for validation of binding mode model. (DOCX)
