# Supplementary material for: Synthesis and biological evaluation of dihydropyrano-[2,3-c]pyrazoles as a new class of PPARγ partial agonists
Source: PLoS One. 2017 Feb 28;12(2):e0162642. doi: 10.1371/journal.pone.0162642 (PMC5330453; doi:10.1371/journal.pone.0162642)
Supplement: S1 File — (PDF) [file pone.0162642.s001.pdf]

Supporting Information

Synthesis and biological evaluation of dihydropyrano[2,3-*c*]-pyrazoles as a new class of PPARγ partial agonists

Katrine Qvortrup[1], Jakob F. Jensen[1], Mikael S. Sørensen[2], Irene Kouskoumvekaki[2], Rasmus K. Petersen[3], Olivier Taboureau[2,4], Karsten Kristiansen[3], and Thomas E. Nielsen[1,5] *

[1]*Department of Chemistry, Technical University of Denmark, DK-2800 Kgs. Lyngby, Denmark*
[2]*Center for Biological Sequence Analysis, Department of Systems Biology, Technical University of Denmark, DK-2800 Kgs. Lyngby, Denmark*
[3]*Department of Biology, University of Copenhagen, Ole Maaløes Vej 5, DK-2200, Copenhagen, Denmark*
[4]*Molécules Thérapeutiques in silico (MTi), Inserm UMR-S 973 - Université Paris Diderot, Bat Lamarck A, 35 Rue Hélène Brion, 75205 Paris, France*
[5]*Singapore Centre on Environmental Life Sciences Engineering, Nanyang Technological University, Singapore 637551, Singapore*

S1: Supporting Information

Contents

General Methods S1
Preparation and spectroscopic data of lead compound 4 S1
Preparation and spectroscopic data of pyranopyrazole library compounds 10a-10ah S9

S2: Model set of compounds for development of binding mode model
S3: Training set of compounds for validation of binding mode model

General Methods

All reagents used were commercially available. All solvents were of HPLC grade. Analytical LC-MS analysis was performed on a Waters AQUITY UPLC system equipped with PDA and SQD MS detector; column: AQUITY UPLC BEH C18 1.7µm, 2.1 x 50mm; column temp: 65 °C; solvent A: 0.1% formic acid (aq); solvent B: 0.1% formic acid (acetonitrile); gradient: 5% B to 100% B in 2.4 min, hold for 0.1 min, total run-time ca. 2.6 min. ^{1}H and ^{13}C NMR 300 MHz spectra were recorded on a Varian Mercury 300 BB spectrometer at room temperature. All NMR spectra were recorded using $CDCl_3$ or DMSO-d_6 as solvents.

Preparation and spectroscopic data of lead compound 4

1,5-bis(chloromethyl)-2,4-dimethylbenzene (12)

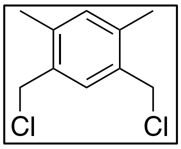

^{1}H NMR (300 MHz, $CDCl_3$): δ = 7.26 (s, 1H, Ar**H**), 7.06 (s, 1H, Ar**H**), 4.58 (s, 4H, C**H**$_2$Cl), 2.40 (s, 6H, 2 × ArC**H**$_3$).
^{13}C NMR (75 MHz, $CDCl_3$): δ = 138.09, 133.61, 133.38, 131.33, 44.50, 18.54.

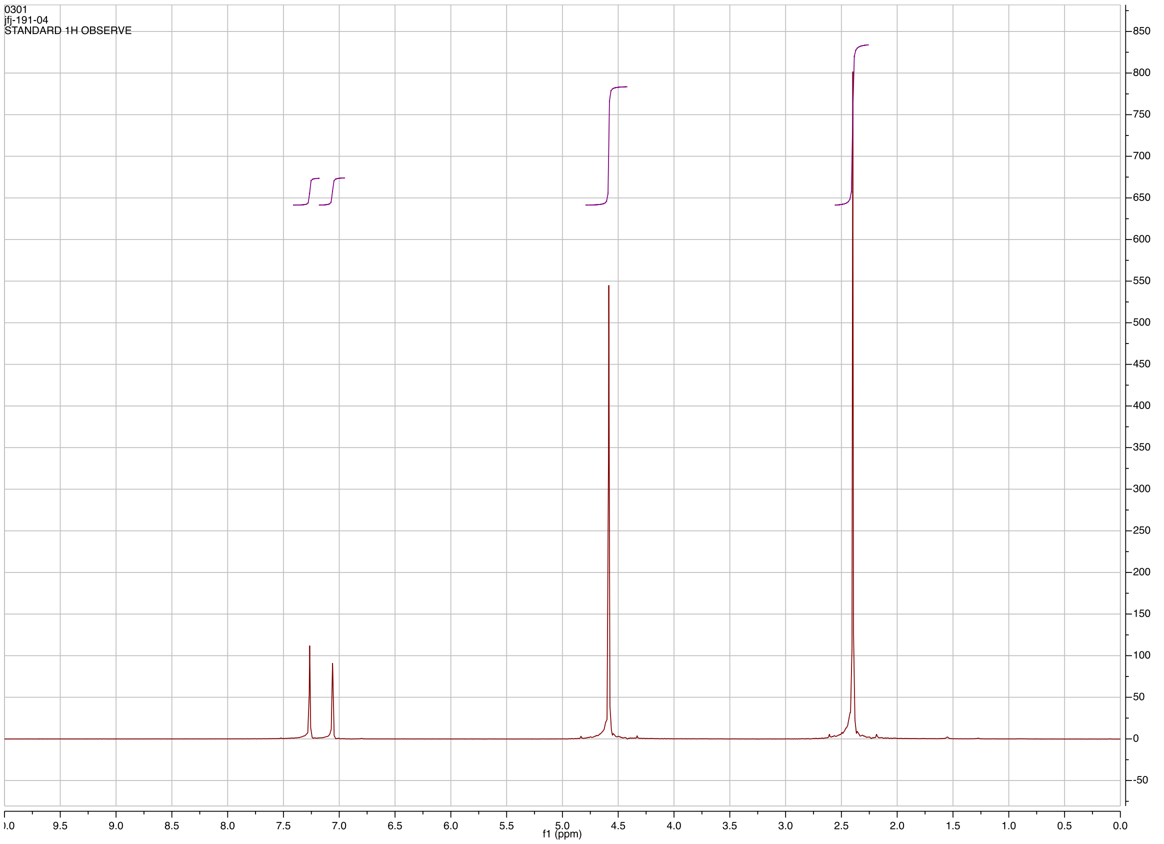
0301
jfj-191-04
STANDARD 1H OBSERVE
f1 (ppm)

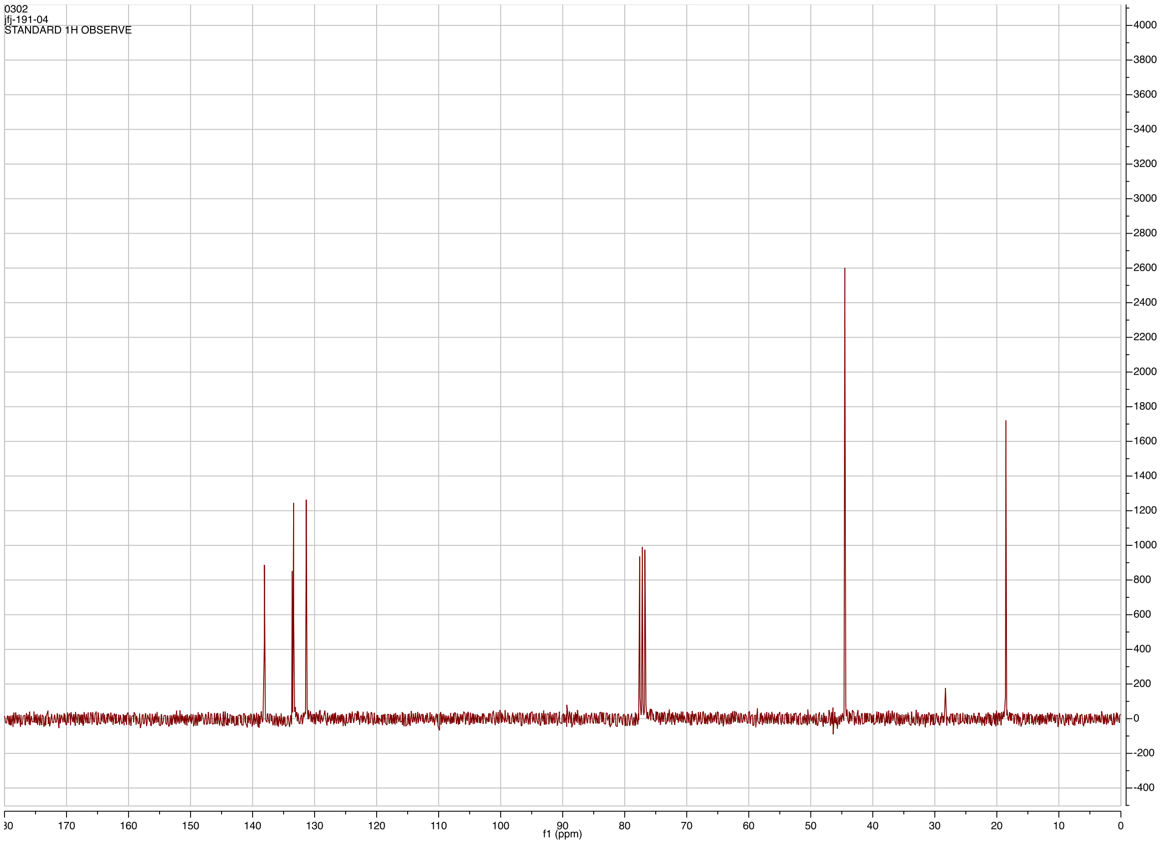
0302
jfj-191-04
STANDARD 1H OBSERVE
f1 (ppm)

4,6-dimethylisophthalaldehyde (13)

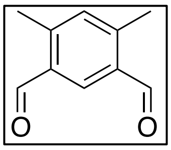

^{1}H NMR (300 MHz, $CDCl_3$): δ = 10.25 (d, J = 0.6 Hz, 2H, 2 × C**H**O), 8.22 (s, 1H, Ar**H**), 7.18 (s, 1H, Ar**H**), 2.70 (d, J = 0.9 Hz, 6H, 2 × ArC**H**$_3$).
^{13}C NMR (75 MHz, $CDCl_3$): δ = 191.71, 146.38, 136.94, 135.82, 132.78, 20.18.

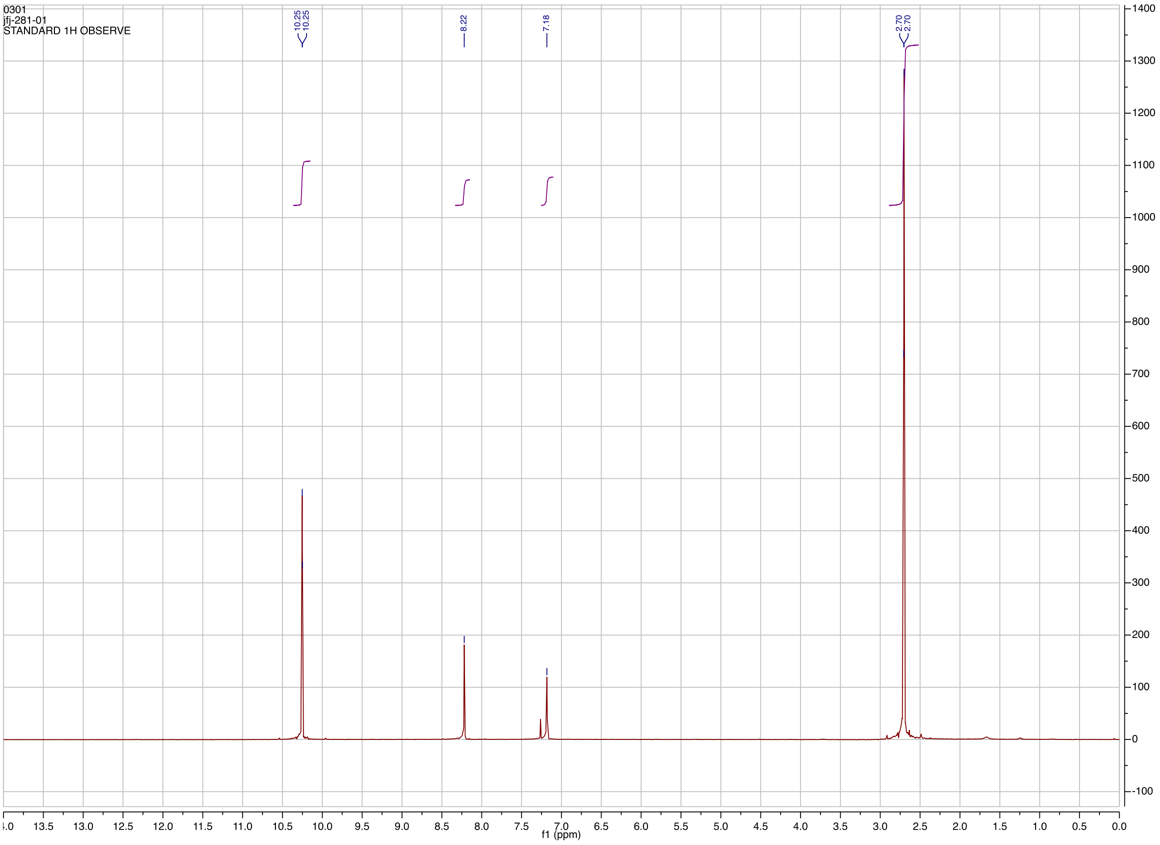

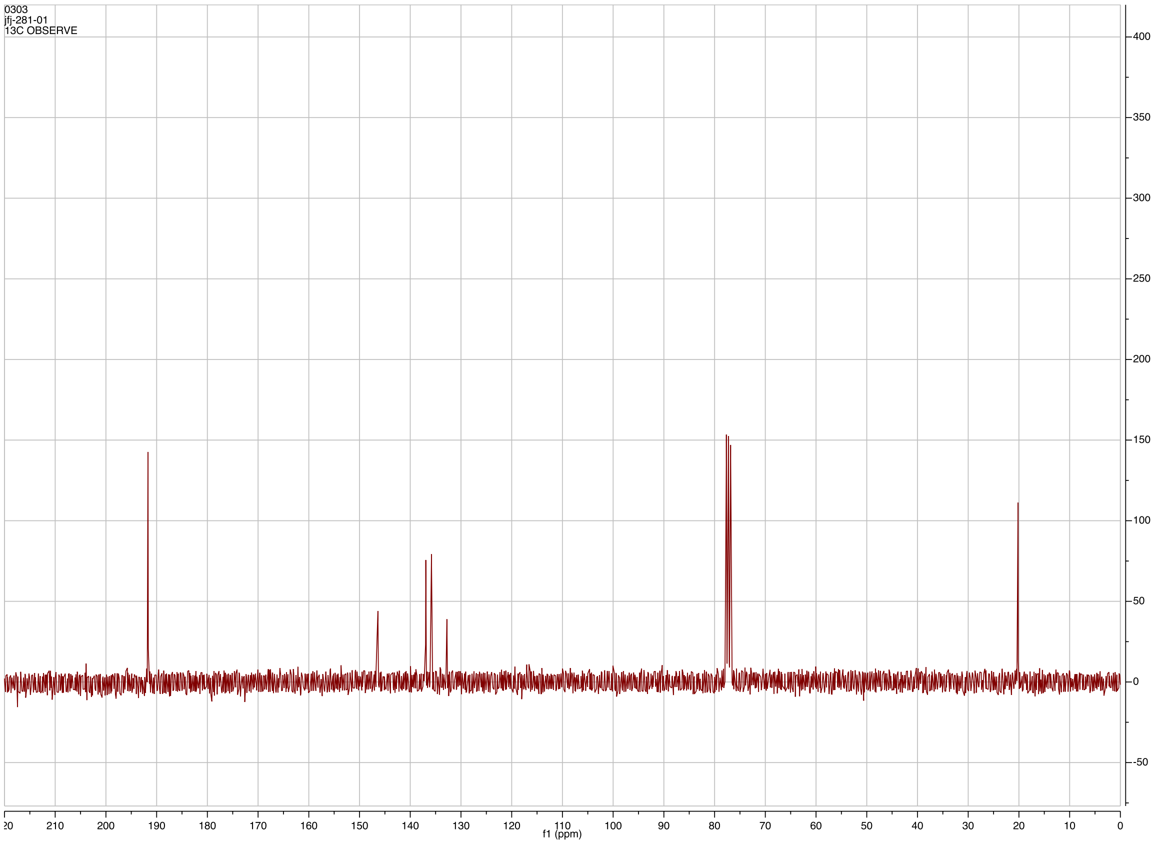

5-(bromomethyl)-2,4-dimethylbenzaldehyde (14)

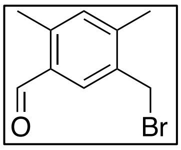

^{1}H NMR (300 MHz, $CDCl_3$): δ = 10.20 (s, 1H, C**H**O), 7.74 (s, 1H, Ar**H**), 7.10 (s, 1H, Ar**H**), 4.53 (s, 2H, C**H**$_2$Br), 2.63 (s, 3H, ArC**H**$_3$) , 2.45 (s, 3H, ArC**H**$_3$).
^{13}C NMR (75 MHz, $CDCl_3$): δ = 191.82, 143.80, 141.37, 134.54, 134.21, 133.46, 132.64, 31.20, 19.27, 19.13.

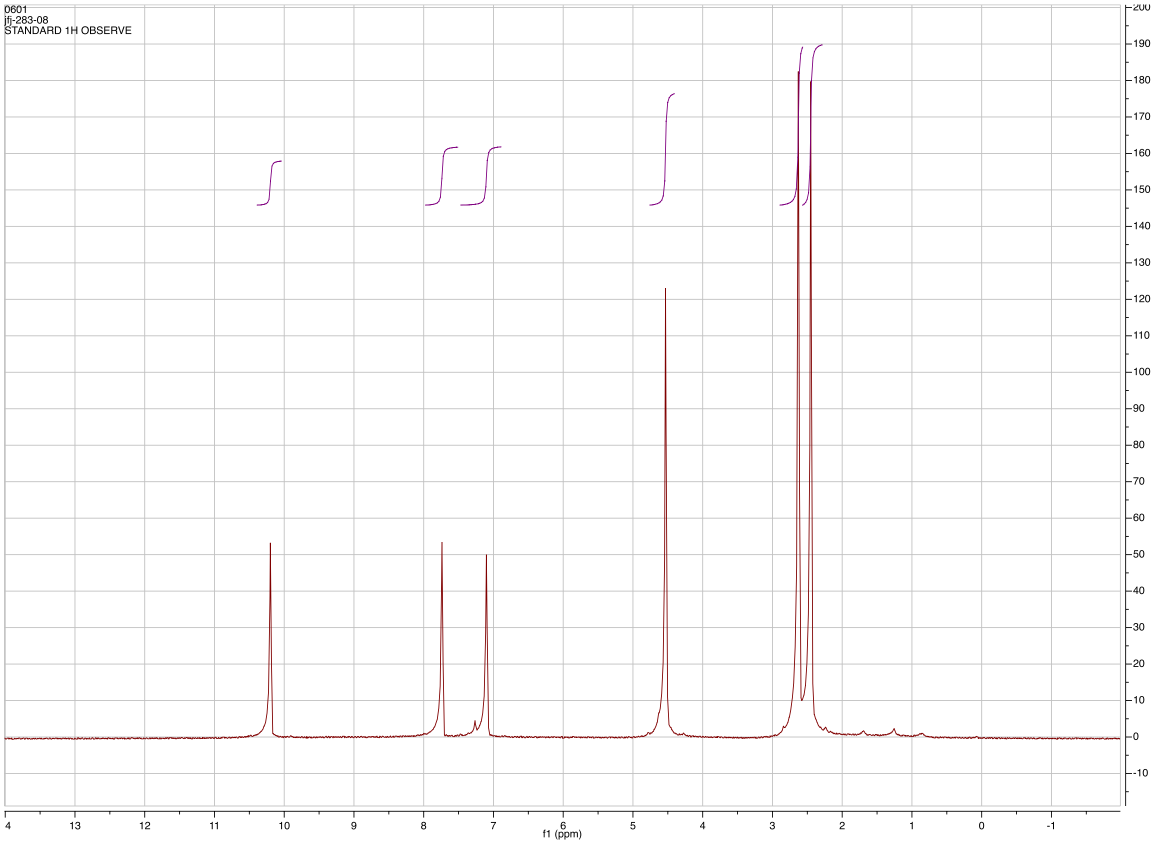
0601
jfj-283-08
STANDARD 1H OBSERVE
f1 (ppm)

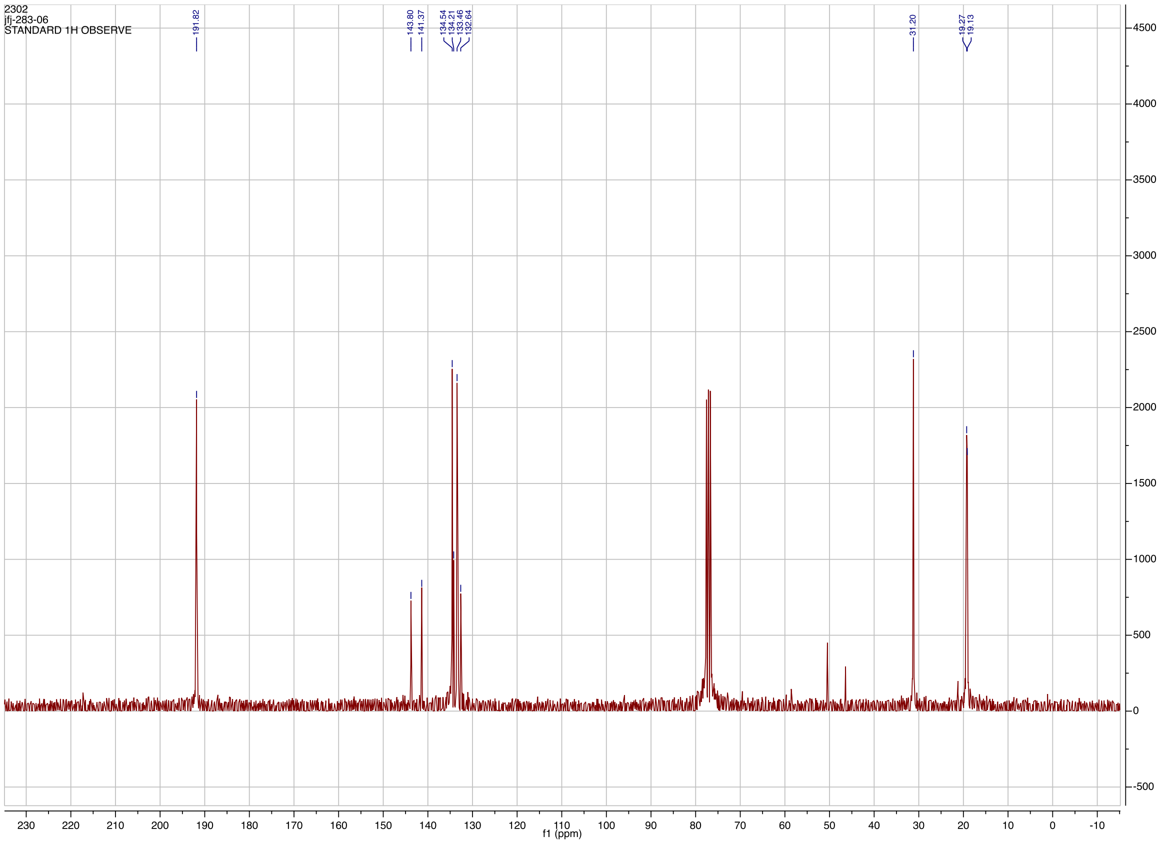
2302
jfj-283-06
STANDARD 1H OBSERVE
191.82
143.80
141.37
134.54
134.21
133.46
132.64
31.20
19.27
19.13
f1 (ppm)

5-((4-chloro-3-methylphenoxy)methyl)-2,4-dimethylbenzaldehyde (15)

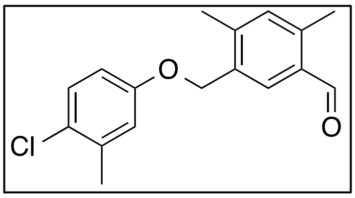

^{1}H NMR (300 MHz, $CDCl_3$): δ = 10.22 (d, J = 0.9 Hz, 1H, C**H**O), 7.84 (s, 1H, Ar**H**), 7.20-7.07 (m, 2H, Ar**H**), 6.83 (dd, J = 8.0, 1.1 Hz, 1H, Ar**H**), 5.02 (s, 2H, OC**H**$_2$Ar) 2.65 (s, 3H, ArC**H**$_3$) , 2.40 (s, 3H, ArC**H**$_3$), 2.22 (d, J = 0.8 Hz, 3H, ArC**H**$_3$).
^{13}C NMR (75 MHz, $CDCl_3$): δ = 192.29, 155.38, 143.27, 140.71, 134.17, 133.27, 132.31, 132.26, 130.71, 129.05, 126.49, 125.59, 112.35, 68.23, 19.31, 19.23, 16.38.

5-Methyl-1H-pyrazol-5(4H)-one (17)

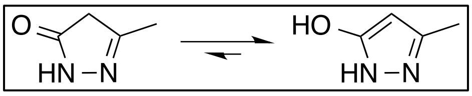

^{1}H NMR (300 MHz, $CDCl_3$): δ = 10.40 (bs, 2H, N**H** + O**H** (tautomeric enol form)), 5.21 (s, 1H, C**H**=COH (tautomeric enol form)), 2.08 (s, 3H, ArC**H**$_3$).
^{13}C NMR (75 MHz, $CDCl_3$): δ = 161.22, 139.54, 89.03 (tautomeric enol form), 49.37 (tautomeric keto form), 11.28.

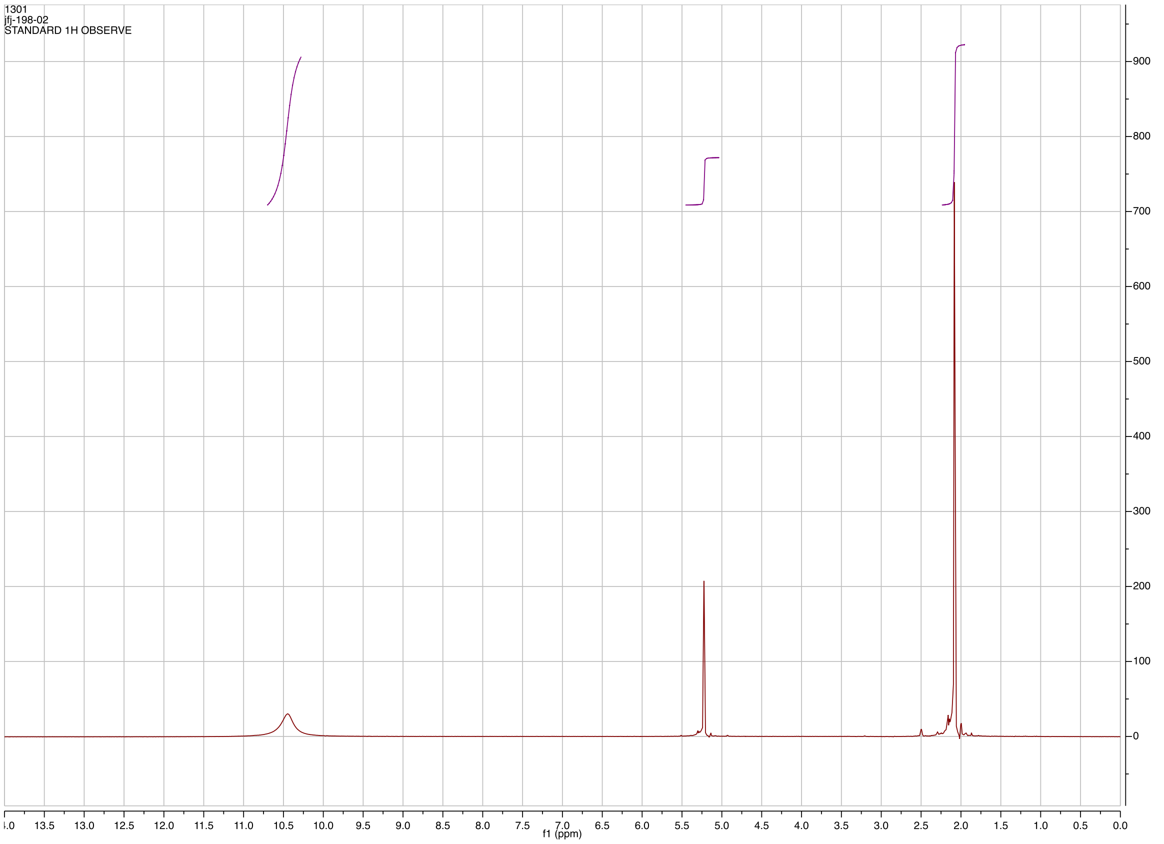

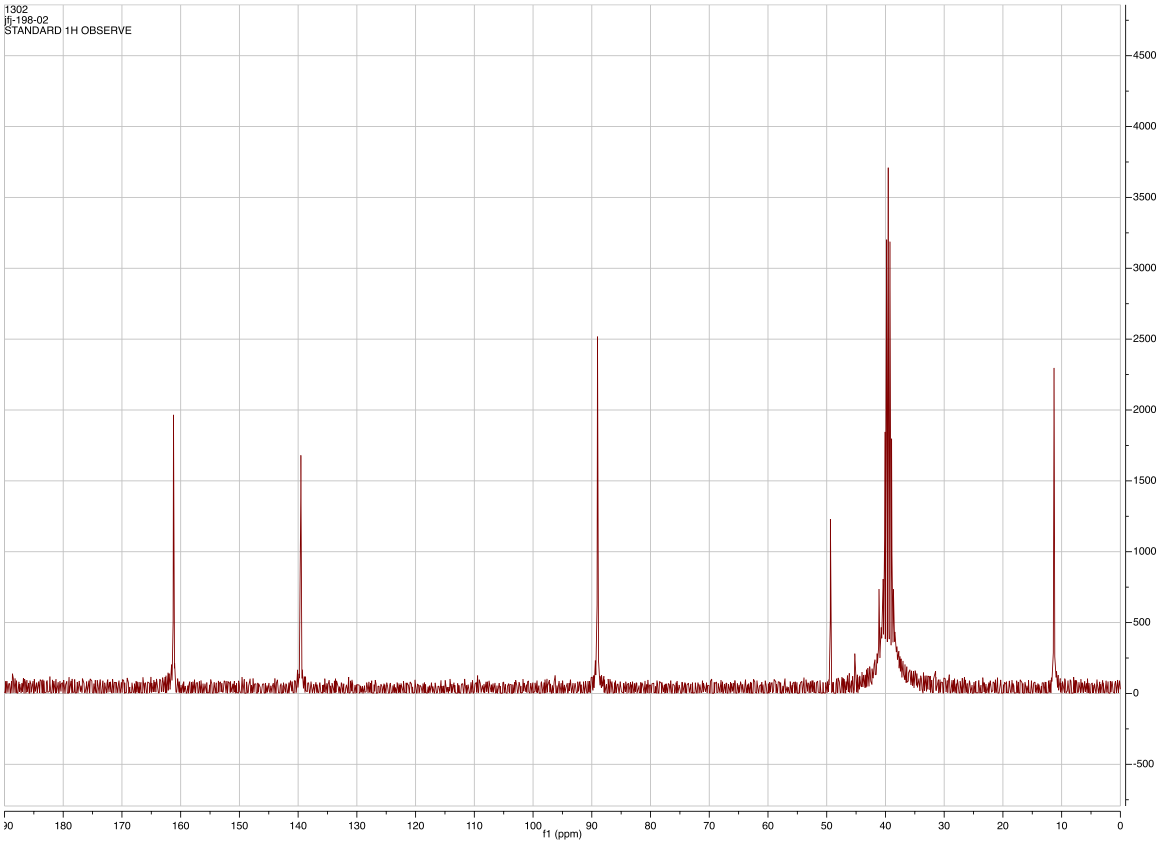

6-amino-4-(5-((4-chloro-3-methylphenoxy)methyl)-2,4-dimethylphenyl)-3-methyl-1,4-dihydropyrano[2,3-*c*]pyrazole-5-carbonitrile (4)

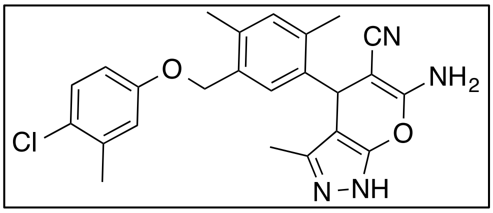

^{1}H NMR (300 MHz, DMSO-d_6): δ = 12.06 (s, 1H, ArN**H**), 7.21-7.18 (m, 1H, Ar**H**), 7.14 (d, J = 8.8 Hz, 1H, Ar**H**), 7.06 (s, 1H, Ar**H**), 7.03-6.96 (m, 2H, Ar**H**), 6.82 (s, 2H, ArN**H$_2$**), 4.99 (s, 2H, ArC**H$_2$**OAr), 4.80 (s, 1H, C**H**Ar), 2.28 (s, 3H, ArC**H$_3$**), 2.24 (s, 3H ArC**H$_3$**), 2.06 (s, 3H, ArC**H$_3$**), 1.63 (s, 3H ArC**H$_3$**);

^{13}C NMR (75 MHz, DMSO-d_6): δ = 160.76, 155.15, 155.09, 139.44, 135.27, 134.38, 134.26, 132.85, 132.19, 129.91, 128.69, 128.40, 126.30, 123.80, 120.88, 113.32, 97.64, 67.80, 56.99, 49.28, 18.53, 17.94, 15.78, 9.57.

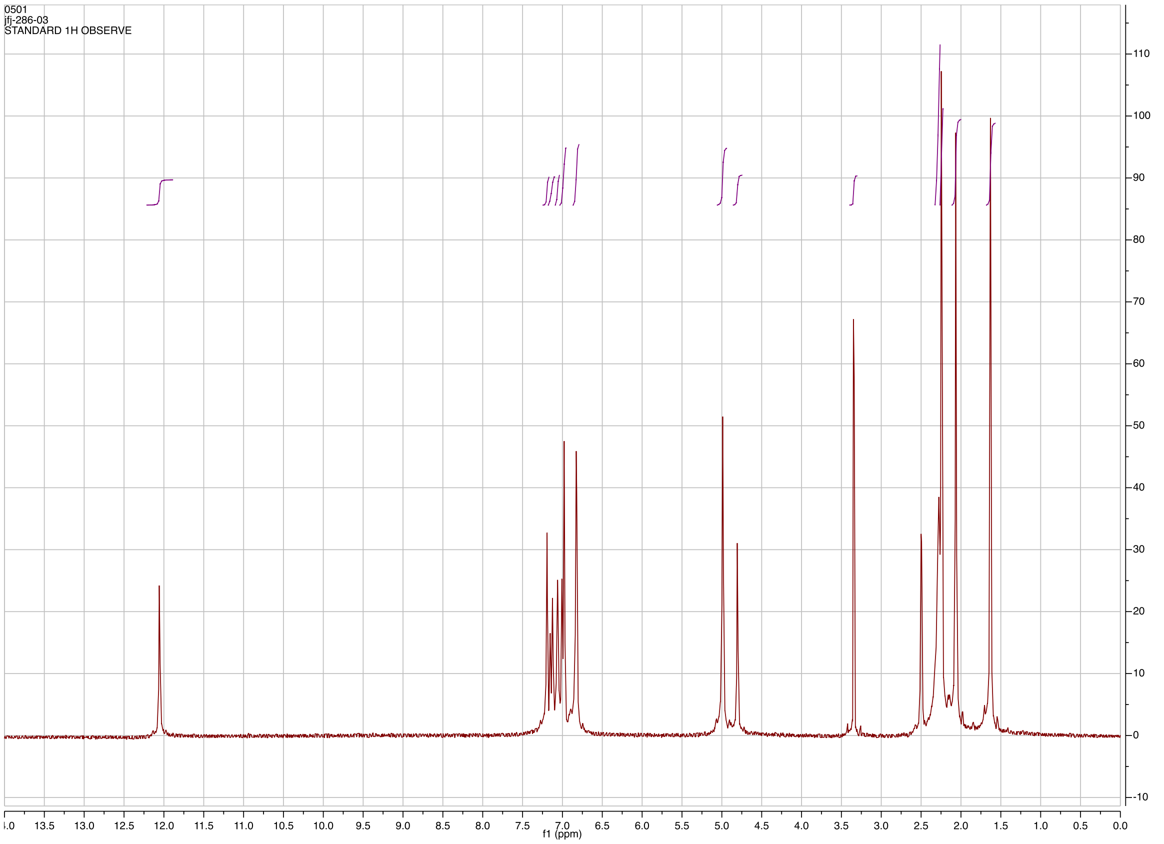
0501
jfj-286-03
STANDARD 1H OBSERVE
f1 (ppm)

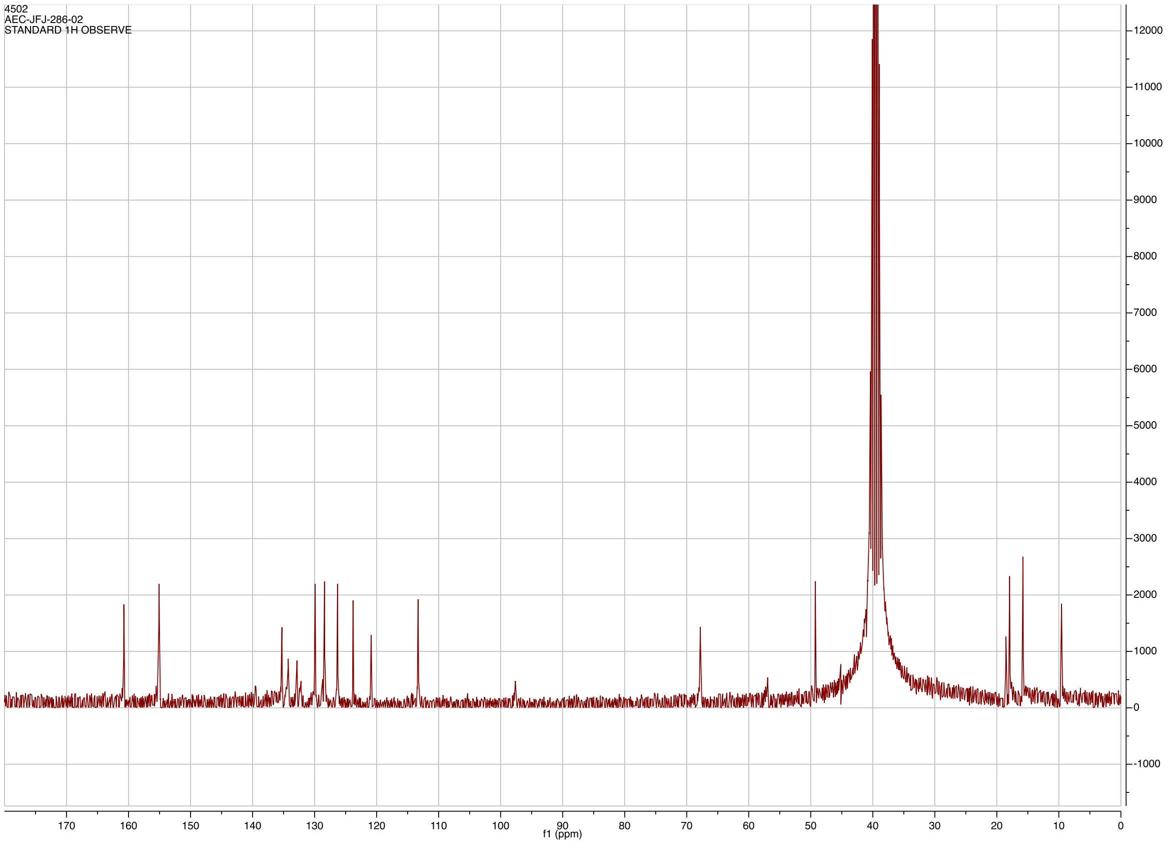
4502
AEC-JFJ-286-02
STANDARD 1H OBSERVE
f1 (ppm)

<u>Preparation and spectroscopic data of pyranopyrazole library compounds 10a-10ah</u>

6-Amino-3-benzyl-4-(2,5-dimethylphenyl)-1,4-dihydropyrano[2,3-*c*]pyrazole-5-carbonitrile (10a)

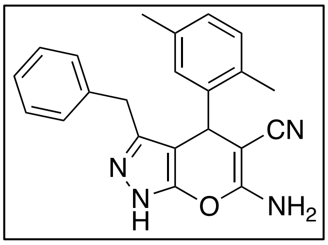

UPLC: Rt = 1.82, 1.88 (2,4-dihydro tautomer);
UPLC-MS (ESI) calculated for $C_{22}H_{21}N_4O$ $[M + H]^-$: *m/z* = 357.2, found *m/z* = 357.2;
^{1}H NMR (300 MHz, DMSO-d_6): δ = 12.33 (s, 1H, ArN**H**), 7.13 (s, 3H, 3 × ArH), 7.04 – 6.79 (m, 4H, 4 × Ar**H**), 6.80 – 6.63 (m, 3H, Ar**H** + ArN**H**$_2$), 4.70 (s, 1H, C**H**Ar), 3.56 (d, *J* = 15.6 Hz, 1H, ArC**H**H), 3.27 (d, *J* = 15.7 Hz, 1H, ArC**H**H), 2.15 (s, 3H, ArC**H**$_3$), 2.11 (s, 3H, ArC**H**$_3$).
^{13}C NMR (75 MHz, DMSO-d_6): δ = 160.53, 155.23, 138.08, 137.34, 135.14, 132.02, 130.52, 129.50, 129.43, 128.31, 127.98, 127.43, 126.28, 120.69, 97.59, 56.90, 49.38, 30.36, 20.68, 18.42;
IR (neat): 1486, 1597, 1632, 2194, 3088, 3243, 3473.

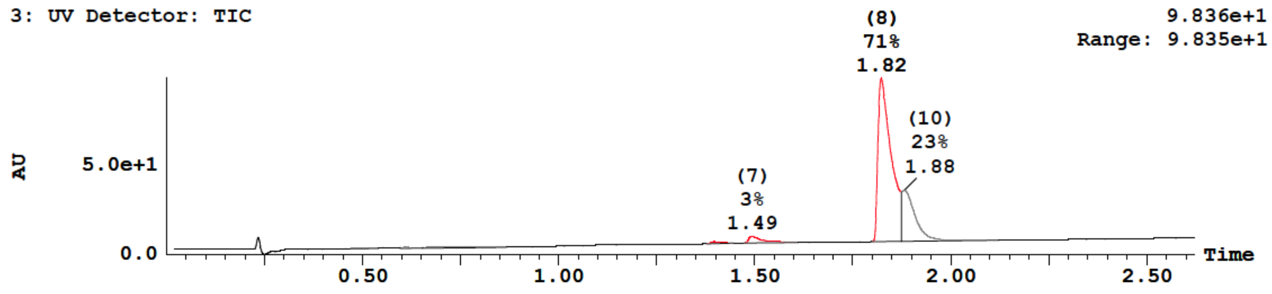

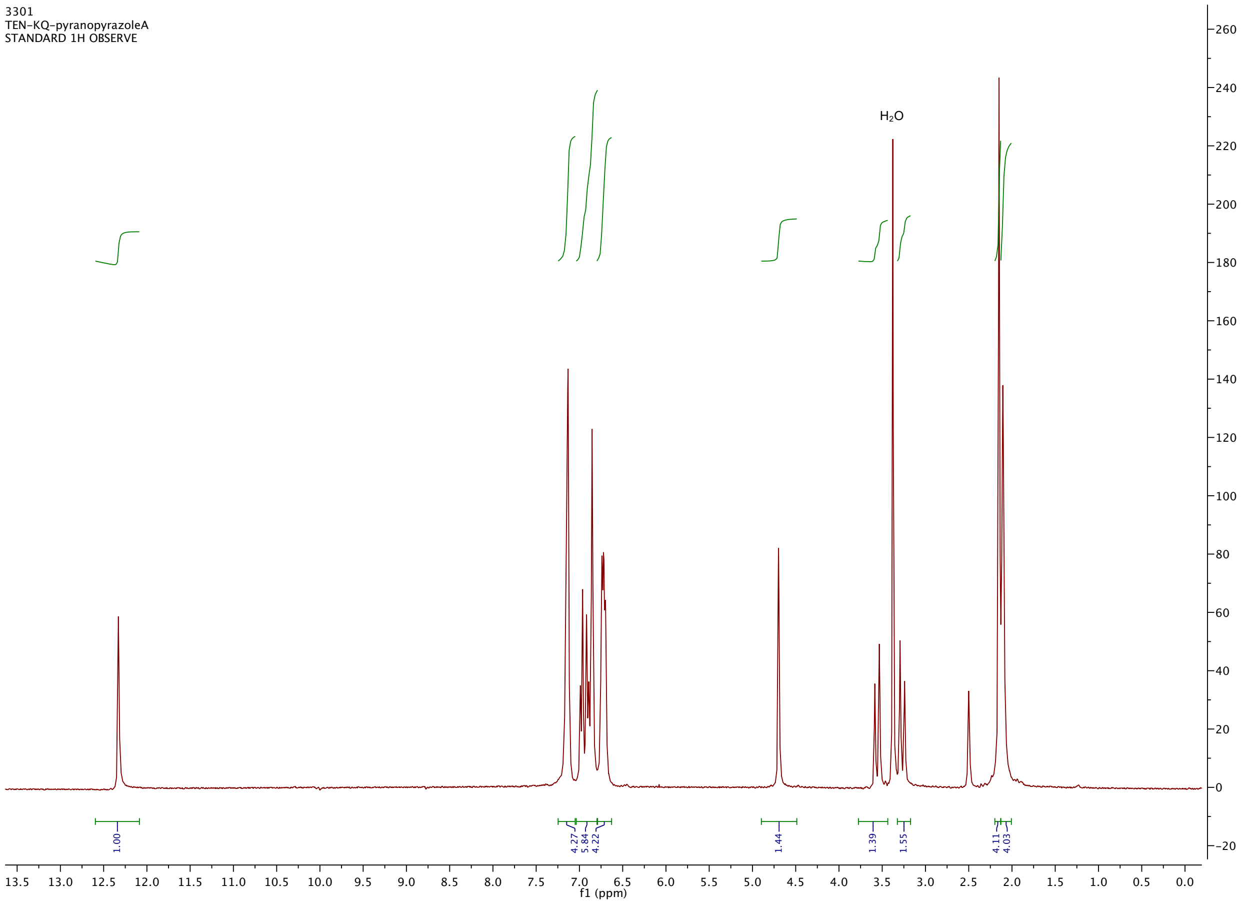
3301
TEN-KQ-pyranopyrazoleA
STANDARD 1H OBSERVE
H$_2$O
f1 (ppm)

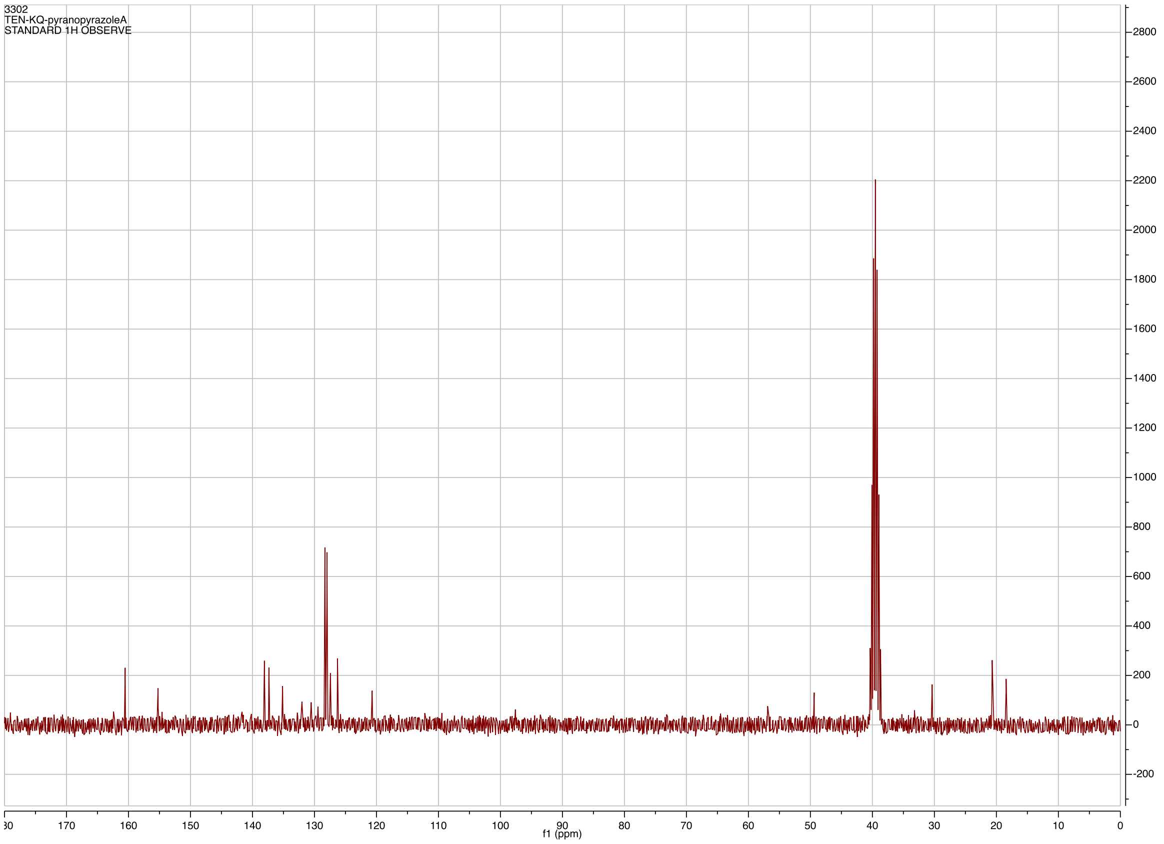
3302
TEN-KQ-pyranopyrazoleA
STANDARD 1H OBSERVE
f1 (ppm)

6-amino-3-benzyl-4-(*o*-tolyl)-1,4-dihydropyrano[2,3-*c*]pyrazole-5-carbonitrile (10b)

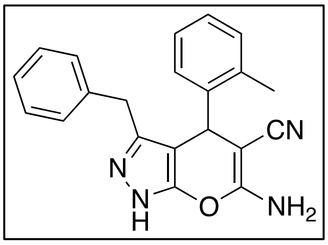

UPLC: Rt = 1.72, 1.78 (2,4-dihydro tautomer).
UPLC-MS (ESI) calculated for $C_{22}H_{21}N_4O$ [M + H]$^-$: *m/z* = 343.2, found *m/z* = 343.2;
^{1}H NMR (300 MHz, DMSO-d_6): δ = 12.34 (s, 1H, ArN**H**), 7.93-6.15 (m, 11H, 9 × ArH + ArNH$_2$), 4.74 (s, 1H, C**H**Ar), 3.58 (d, *J* = 16.1 Hz, 1H, ArC**H**H), 3.24 (d, *J* = 16.2 Hz, 1H, ArCH**H**), 2.12 (s, 3H, ArC**H**$_3$);
^{13}C NMR (75 MHz, DMSO-d_6): δ = 160.59, 155.23, 141.95, 138.07, 137.34, 135.12, 130.62, 129.07, 128.38, 127.99, 126.73, 126.37, 126.31, 120.66, 97.54, 56.76, 56.09, 30.35, 18.77.

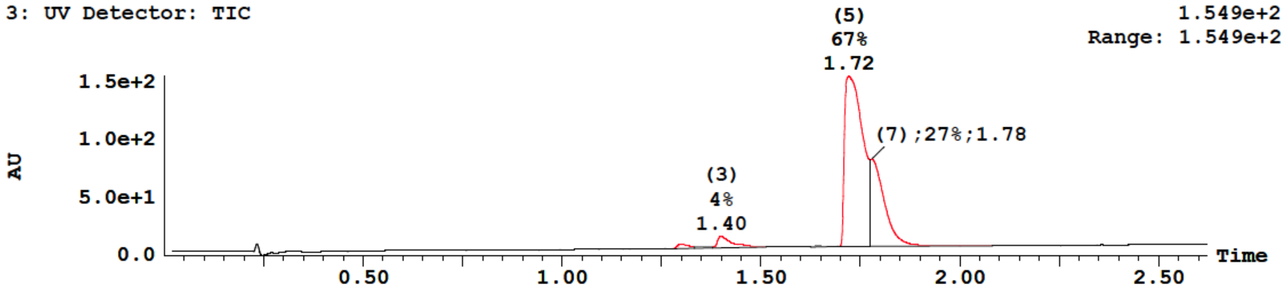

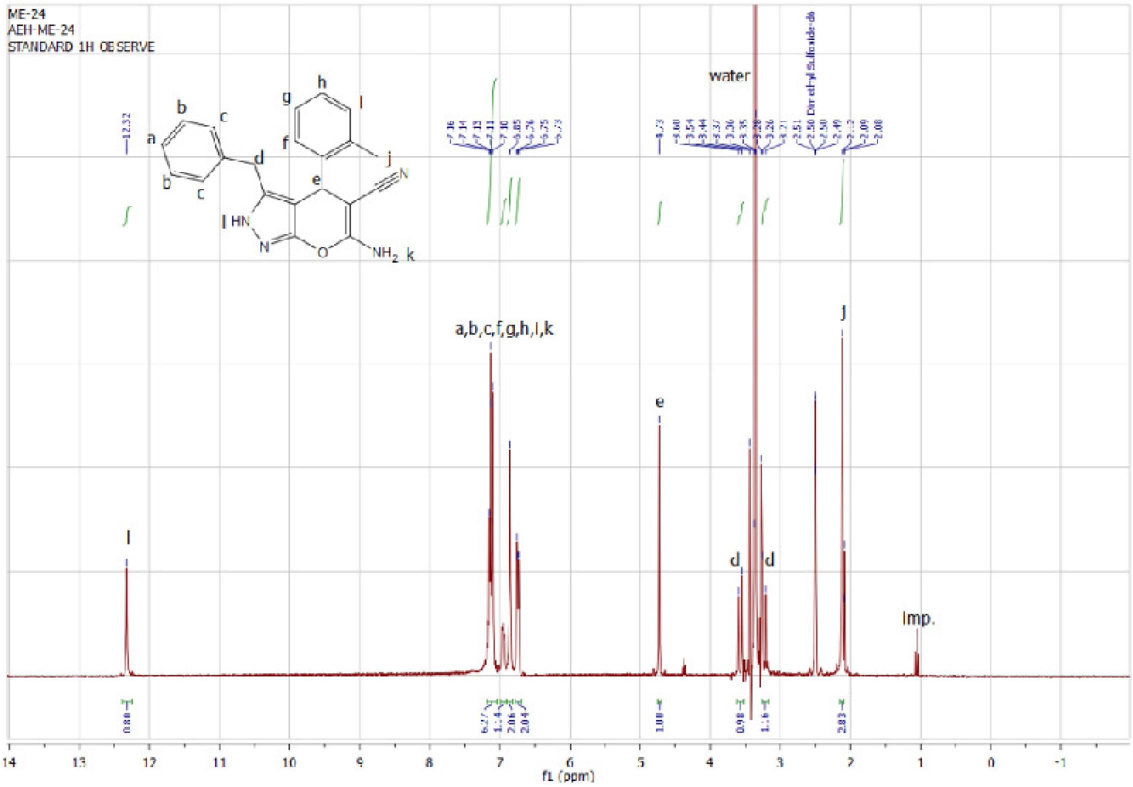

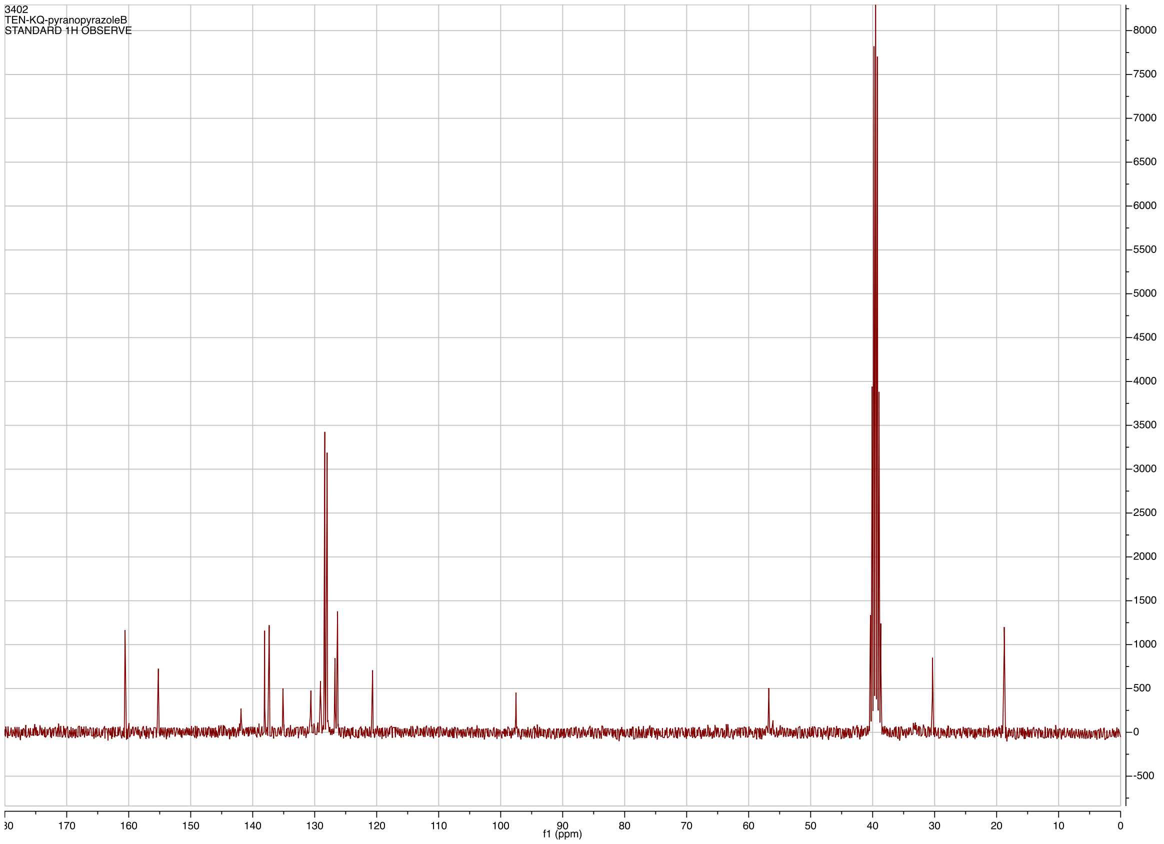

3402
TEN-KQ-pyranopyrazoleB
STANDARD 1H OBSERVE
f1 (ppm)

6-amino-3-benzyl-4-(2,4-dimethylphenyl)-1,4-dihydropyrano[2,3-*c*]pyrazole-5-carbonitrile (10c)

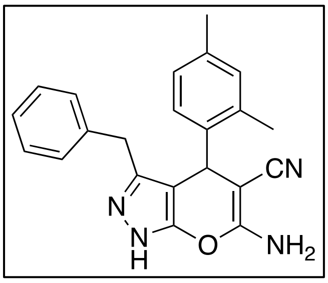

UPLC: Rt = 1.84, 1.89 (2,4-dihydro tautomer);
UPLC-MS (ESI) calculated for $C_{22}H_{21}N_4O$ [M + H]$^-$: *m/z* = 357.2, found *m/z* = 357.2;
^{1}H NMR (300 MHz, DMSO-d_6): δ = 12.33 (s, 1H, ArN**H**), 7.23-7.10 (m, 3H, Ar**H**), 7.01-6.81 (m, 4H, Ar**H**), 6.78-6.65 (m, 3H, Ar**H** + ArN**H$_2$**), 4.70 (s, 1H, C**H**Ar), 3.56 (d, *J* = 15.6 Hz, 1H, ArC**H**H), 3.27 (d, *J* = 15.7 Hz, 1H, ArCH**H**), 2.15 (s, 3H, ArC**H$_3$**), 2.11 (s, 3H, ArC**H$_3$**);
^{13}C NMR (75 MHz, DMSO-d_6): δ = 160.49, 155.20, 138.94, 138.05, 137.37, 135.64, 134.87, 131.26, 129.05, 128.33, 127.99, 126.95, 126.24, 120.67, 97.63, 56.94, 50.43, 30.33, 20.58, 18.69.

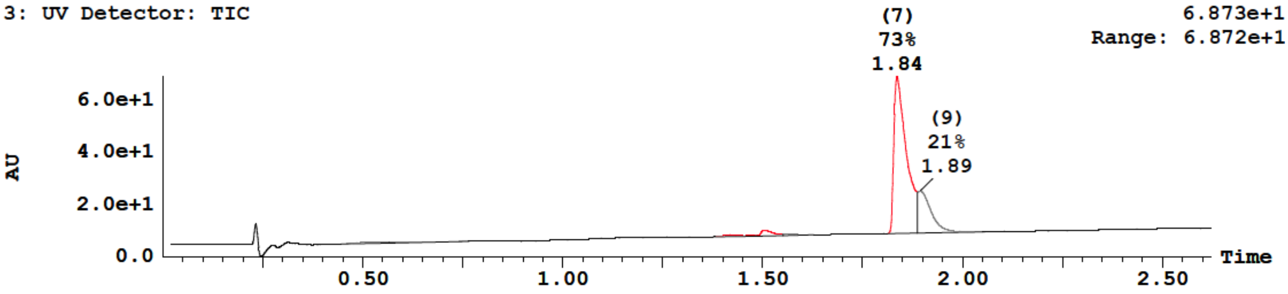

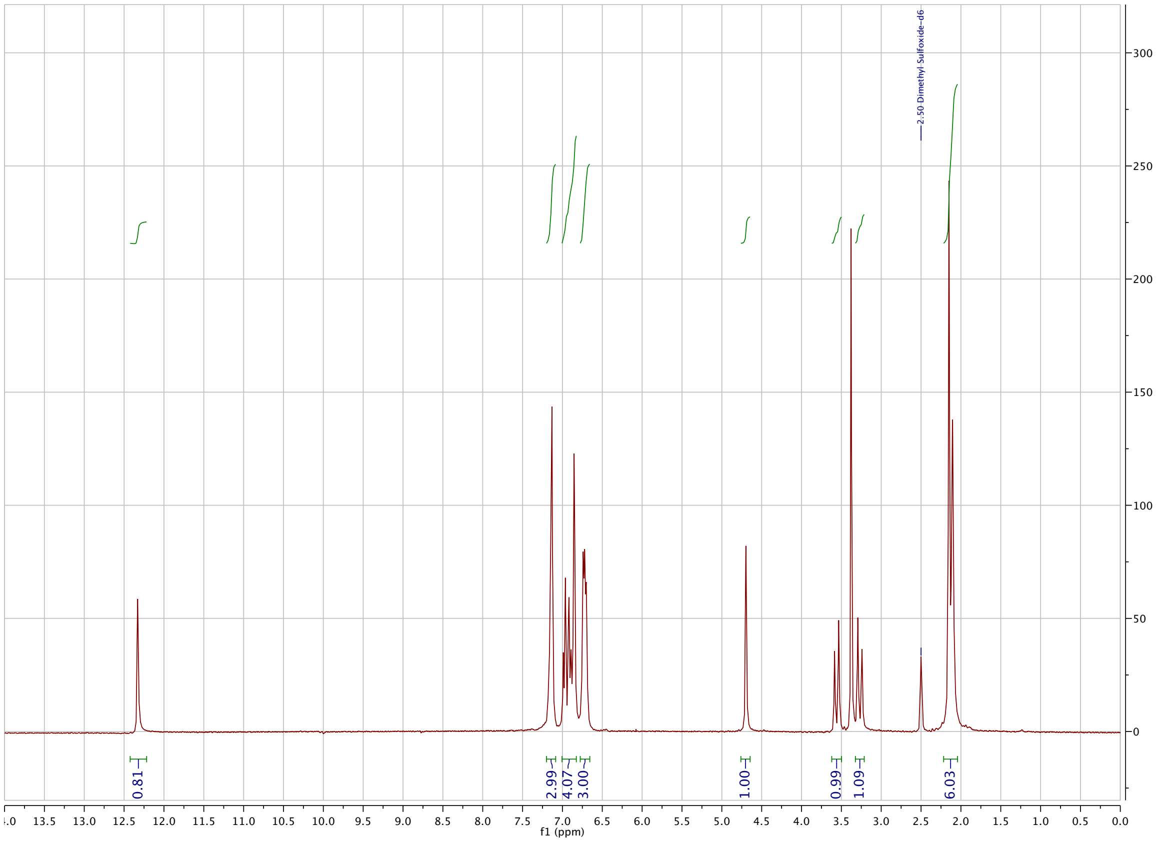

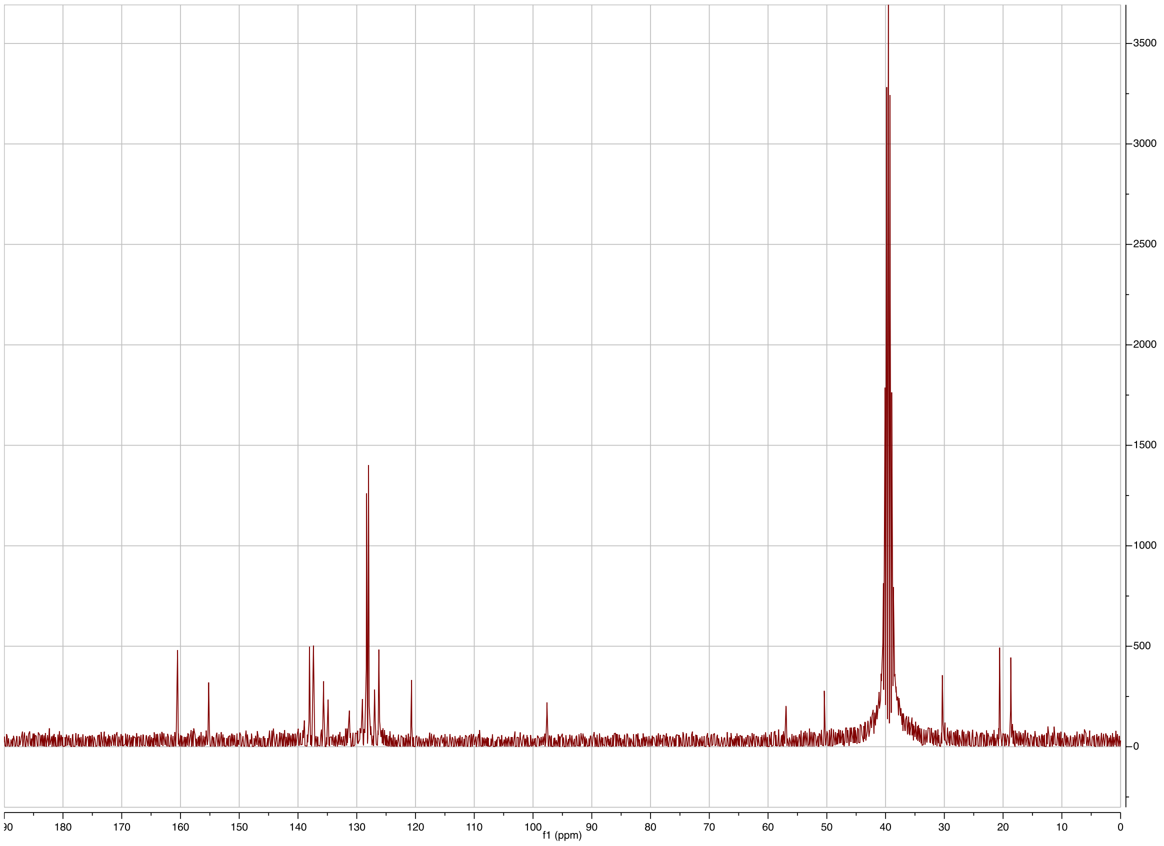
3500
3000
2500
2000
1500
1000
500
0
180
170
160
150
140
130
120
110
100
90
80
70
60
50
40
30
20
10
0
f1 (ppm)

6-amino-3-benzyl-4-(4-methoxy-2,3-dimethylphenyl)-1,4-dihydropyrano[2,3-*c*]pyrazole-5-carbonitrile (10d)

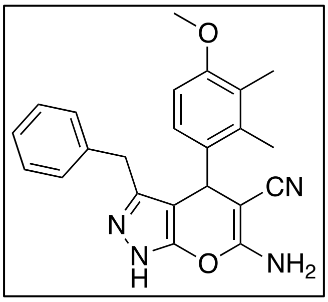

UPLC: Rt = 1.81, 1.87 (2,4-dihydro tautomer);
UPLC-MS (ESI) calculated for $C_{23}H_{23}N_4O_2$ $[M + H]^-$: *m/z* =387.2, found *m/z* = 387.2;
^{1}H NMR (300 MHz, DMSO-d_6): δ 12.30 (s, 1H, ArN**H**), 7.18-7.08 (m, 3H, Ar**H**), 6.86-6.69 (m, 6H, 4 × Ar**H** + ArN**H**$_2$), 4.74 (s, 1H, C**H**Ar), 3.73 (s, 3H, ArOC**H**$_3$), 3.55 (d, *J* = 15.7 Hz, 1H, ArC**H**H), 3.26 (d, *J* = 15.7 Hz, 1H, ArC**H**H), 2.03 (s, 6H, 2 × ArC**H**$_3$);
^{13}C NMR (101 MHz, DMSO-d_6): δ = 160.3, 155.7, 155.1, 138.0, 137.4, 137.3, 135.0, 128.1, 127.9, 126.1, 120.7, 108.2, 98.3, 58.0, 55.3, 30.3, 14.7, 11.9.

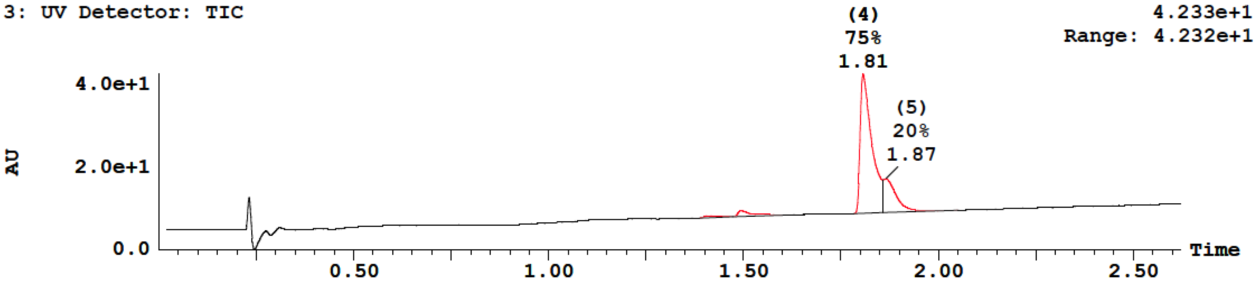

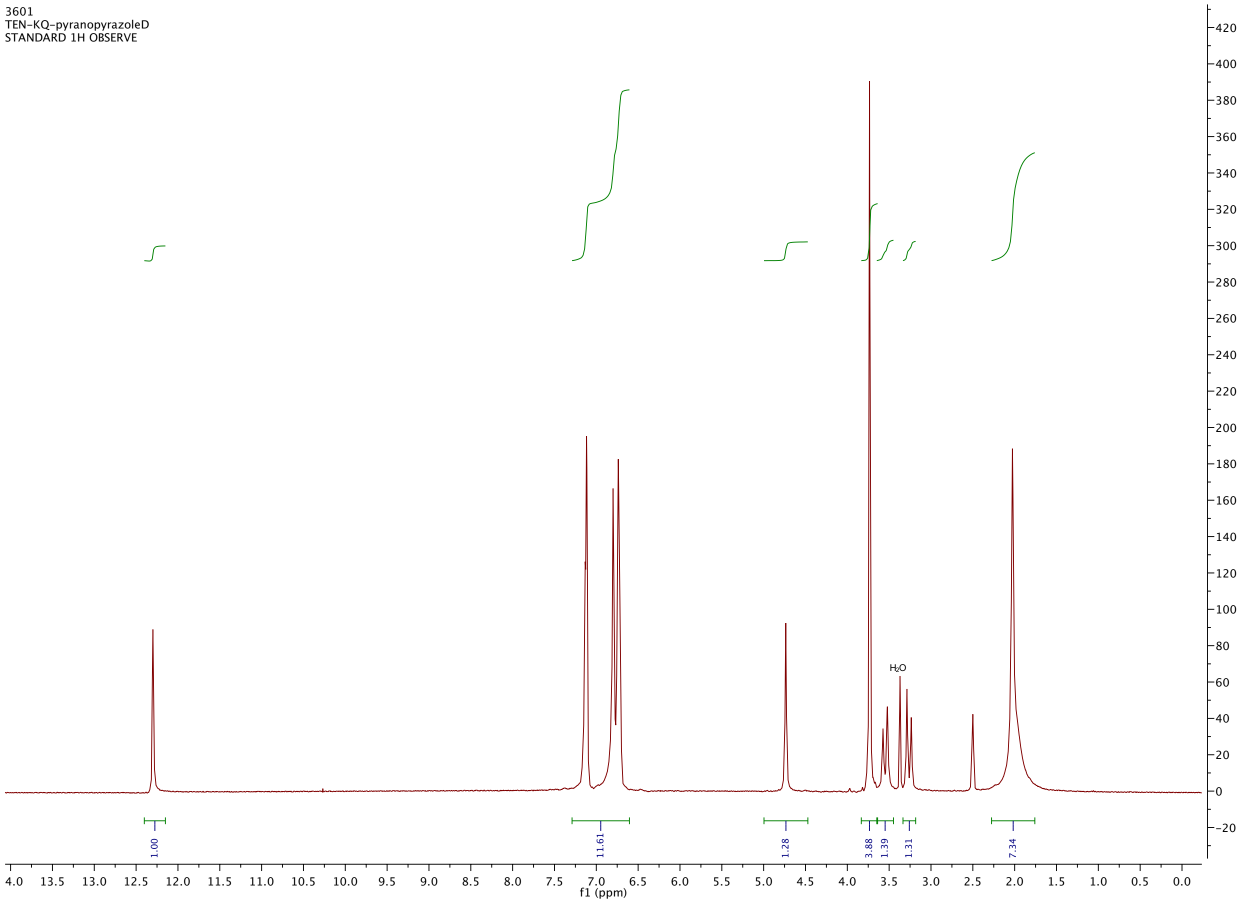

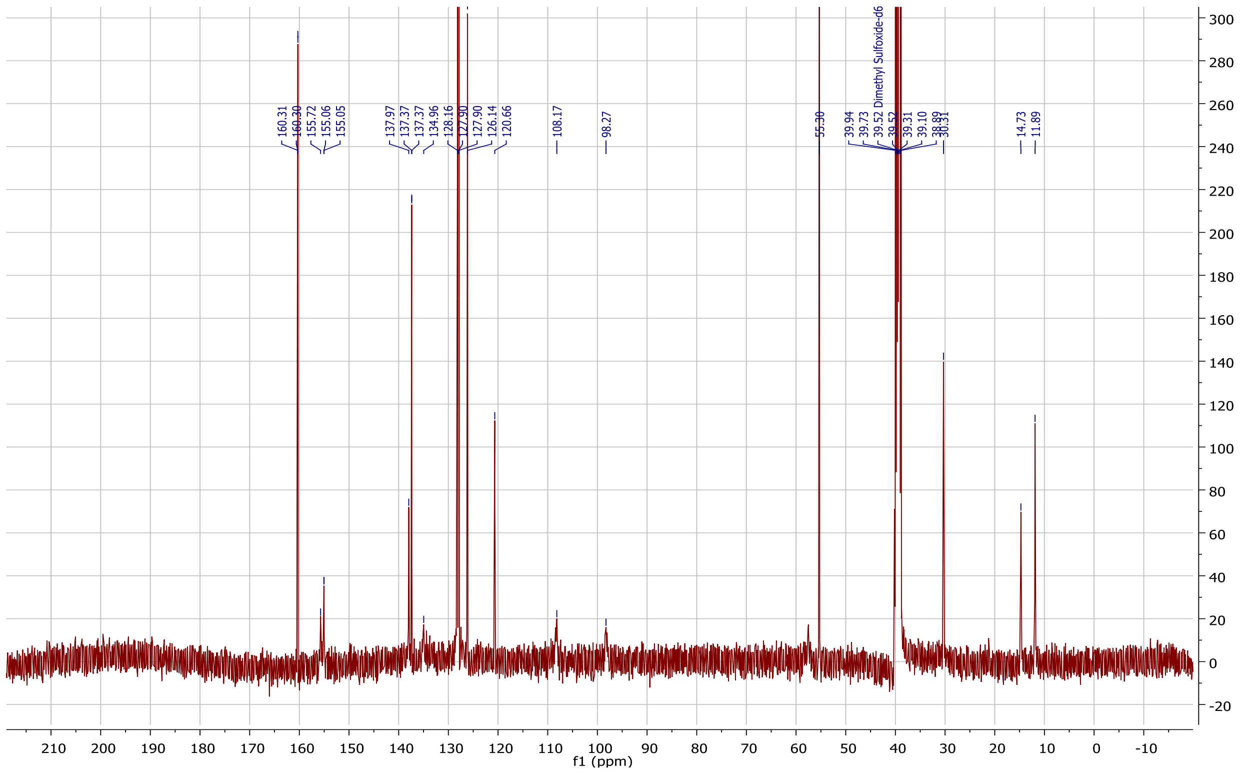
160.31
160.30
155.72
155.06
155.05
137.97
137.37
137.37
134.96
128.16
127.90
127.90
126.14
120.66
108.17
98.27
55.30
39.94
39.73
39.52 Dimethyl Sulfoxide-d6
39.52
39.31
39.10
38.89
30.31
14.73
11.89
f1 (ppm)

6-amino-4-(4-chlorophenyl)-3-phenyl-1,4-dihydropyrano[2,3-*c*]pyrazole-5-carbonitrile (10e)

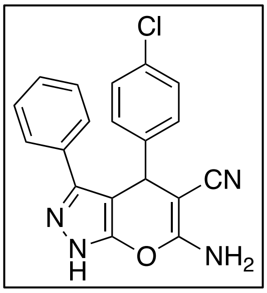

UPLC: Rt = 1.84, 1.89 (2,4-dihydro tautomer);
UPLC-MS (ESI) calculated for $C_{19}H_{14}ClN_4O_2$ $[M + H]^-$: *m/z* = 349.1 (^{35}Cl), 351.1 (^{37}Cl), found *m/z* = 349.1 (100 %), 351.1 (30 %);
^{1}H NMR (400 MHz, DMSO-d_6): δ = 12.93 (s, 1H, ArN**H**), 7.50-7.43 (m, 2H, Ar**H**), 7.37-7.23 (m, 5H, 5 × Ar**H**), 7.17-7.09 (m, 2H, 2 × Ar**H**), 6.98 (s, 2H, ArN**H**$_2$), 5.06 (s, 1H, C**H**Ar);
^{13}C NMR (100 MHz, DMSO-d_6): δ = 160.56, 156.35, 144.04, 138.47, 131.54, 129.68, 129.07, 128.93, 128.87, 128.74, 126.69, 120.92, 97.40, 58.24, 36.48.

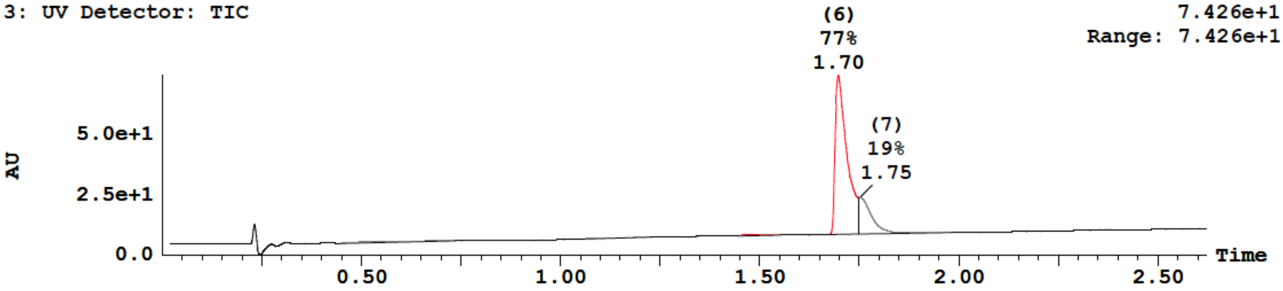

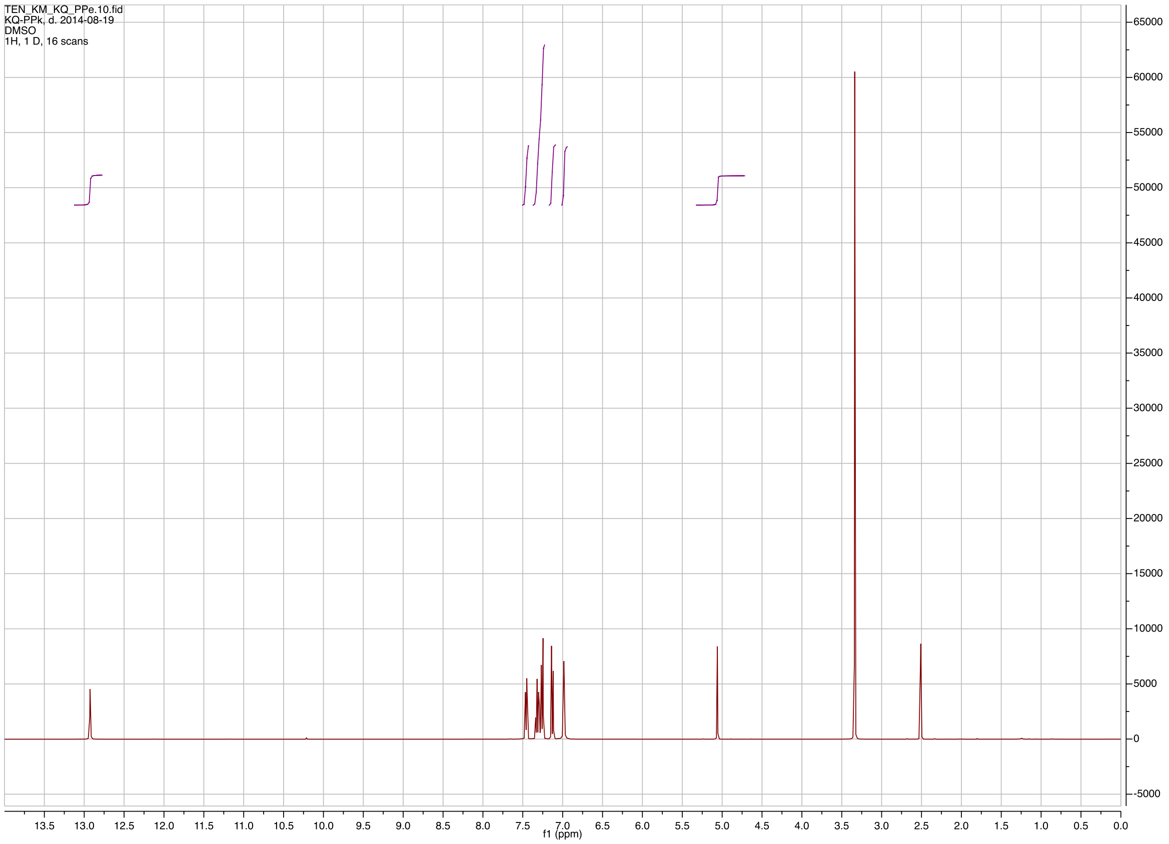

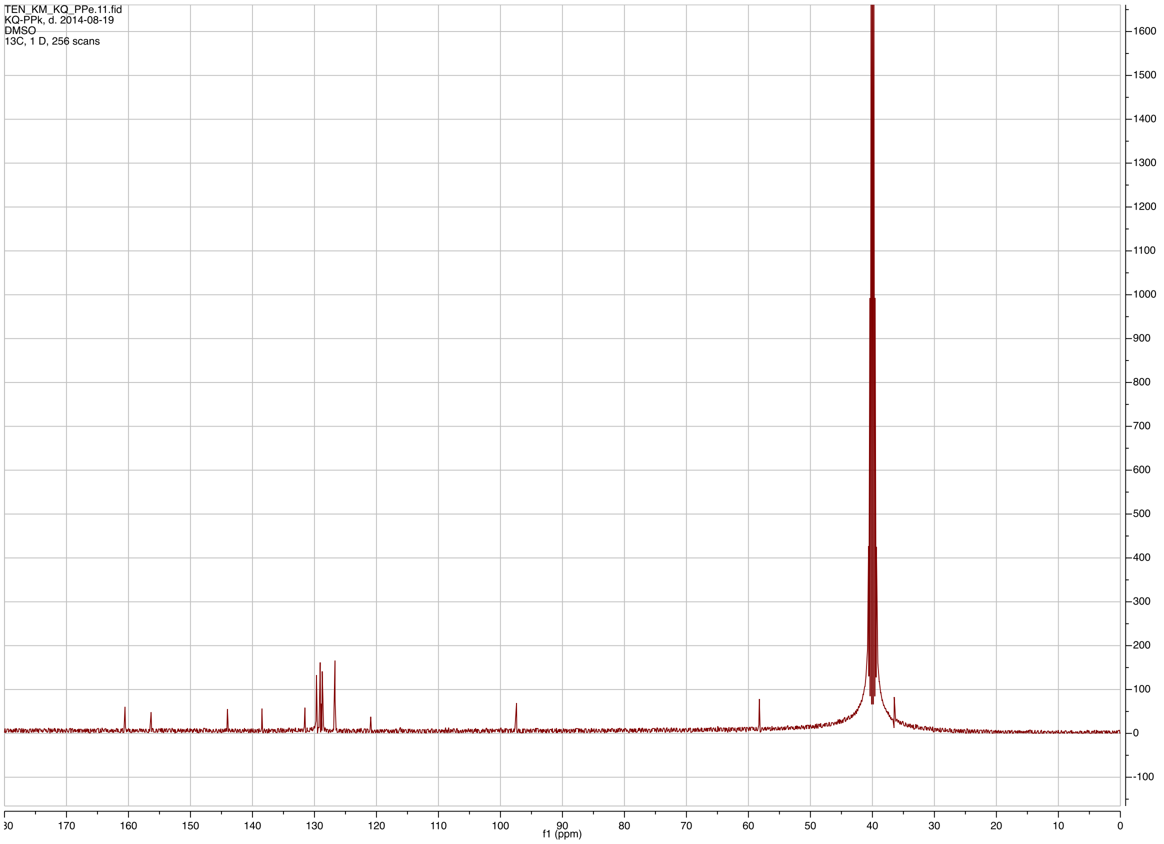

TEN_KM_KQ_PPe.11.fid
KQ-PPk, d. 2014-08-19
DMSO
13C, 1 D, 256 scans
f1 (ppm)

6-amino-4-(2,4-dichlorophenyl)-3-phenyl-1,4-dihydropyrano[2,3-*c*]pyrazole-5-carbonitrile (10f)

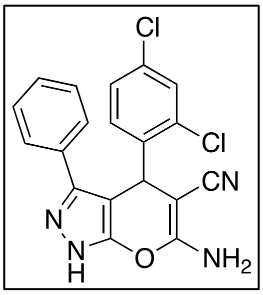

UPLC-UV: Rt = 1.80, 1.86 (2,4-dihydro tautomer);
UPLC-MS (ESI) calculated for $C_{19}H_{12}Cl_2N_4O$ [M + H]$^-$: *m/z* = 383.1 (2 x ^{35}Cl), 385.0 (1 x ^{37}Cl, 1 x ^{35}Cl,), found *m/z* = 383.1 (100 %), 385.1 (65 %);
^{1}H NMR (400 MHz, DMSO-d_6): δ = 12.92 (s, 1H, ArN**H**), 7.50-7.00 (m, 10H, 8 × Ar**H** + 2 × ArN**H**$_2$), 5.45 (s, 1H, C**H**Ar);
^{13}C NMR (100 MHz, DMSO-d_6): δ = 160.58, 156.10, 140.22, 138.05, 132.97, 132.08, 131.98, 128.58 (two overlapping signals), 128.38, 127.80, 126.13 (two overlapping signals), 120.02, 96.14, 55.96, 49.26.

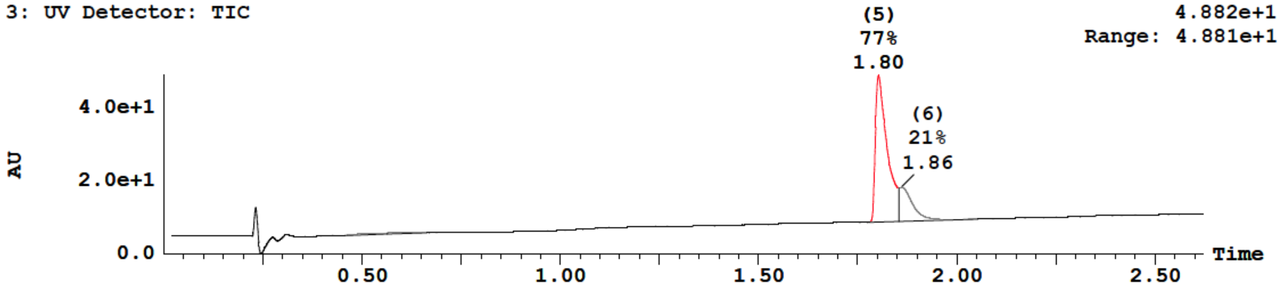

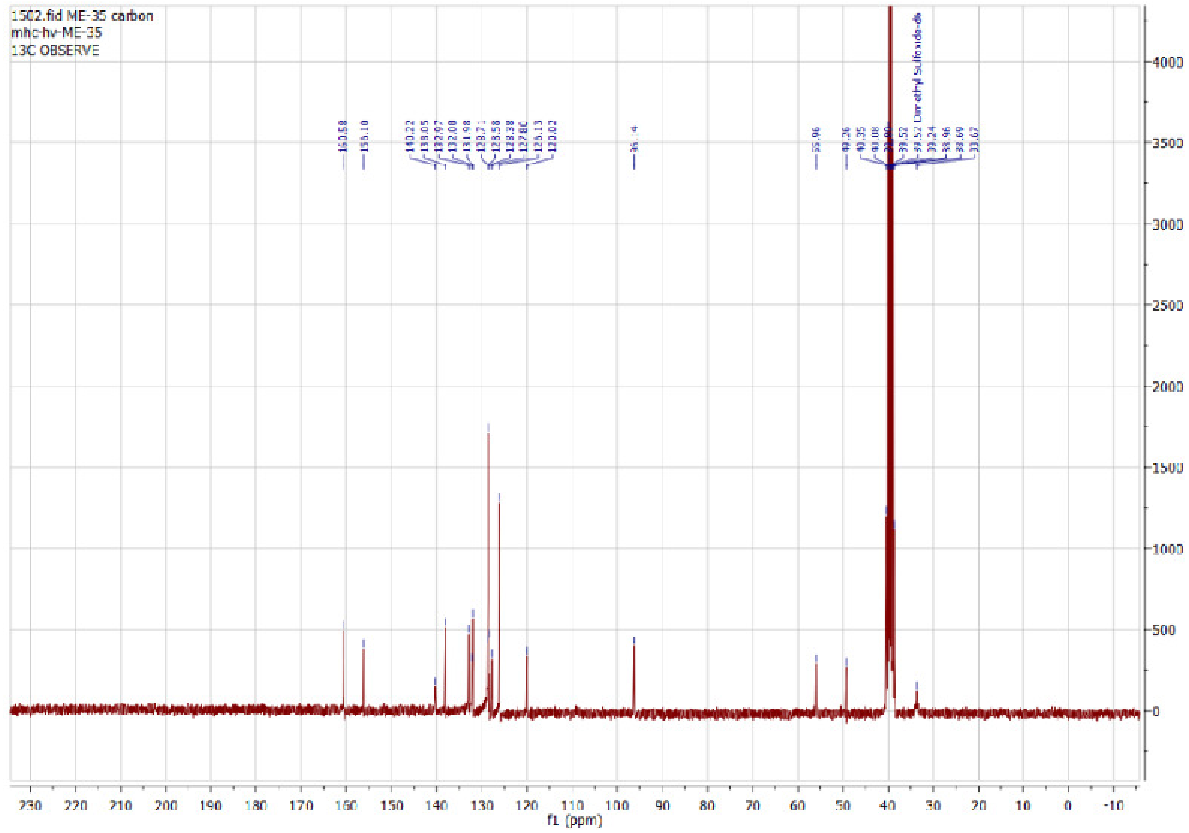

6-amino-4-(2,5-dimethylphenyl)-3-phenyl-1,4-dihydropyrano[2,3-*c*]pyrazole-5-carbonitrile (10g)

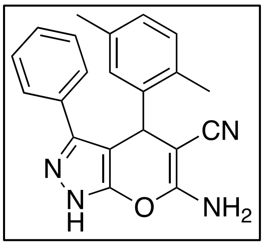

UPLC-UV: Rt = 1.73, 1.79 (2,4-dihydro tautomer). UPLC-MS (ESI) calculated for $C_{21}H_{19}N_4O$ [M + H]$^-$: *m/z* = 343.2, found *m/z* = 343.2.

^{1}H NMR (300 MHz, DMSO-*d*$_6$): δ = 12.83 (s, 1H, ArN**H**), 7.34 – 7.21 (m, 5H, Ar**H**), 7.00 – 6.88 (m, 3H, 2 × Ar**H** + 1 × ArN**H**H), 6.82 (dd, *J* = 7.8, 1.8 Hz, 1H, Ar**H**), 6.71 (s, 1H, ArN**H**H), 5.14 (s, 1H, C**H**Ar), 2.41 – 2.22 (s, 3H, ArC**H**$_3$), 2.17 – 1.96 (s, 3H, ArC**H**$_3$).

^{13}C NMR (75 MHz, DMSO-*d*$_6$): δ = 160.12, 156.32, 142.61, 137.81, 135.11, 131.54, 130.39, 128.97, 128.82, 128.52, 128.37, 127.34, 126.16, 120.70, 97.78, 57.71, 33.11, 20.69, 18.56.

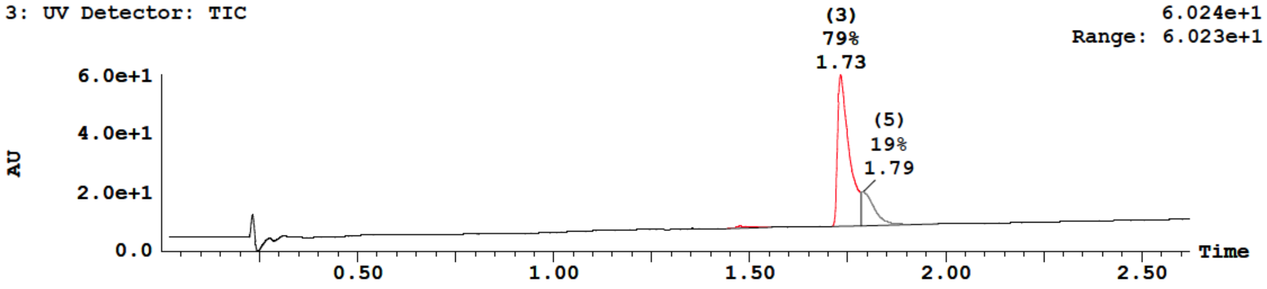

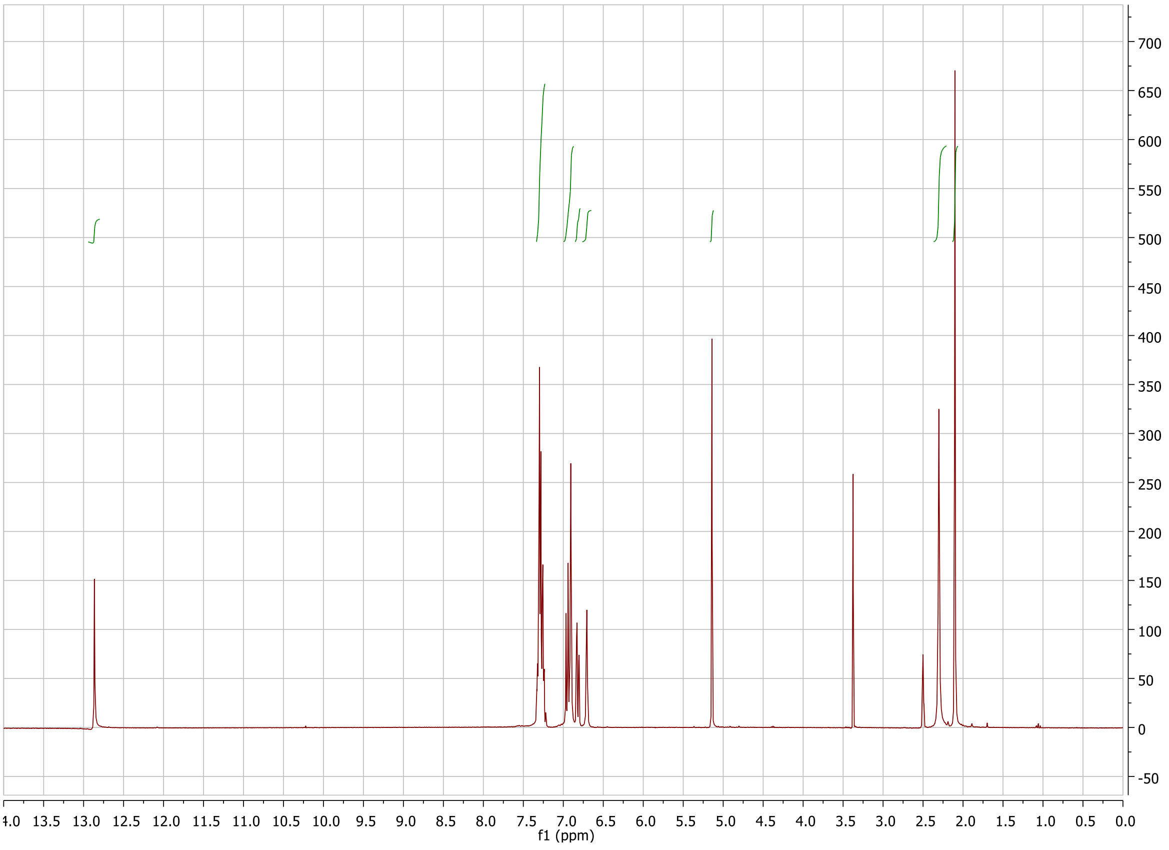

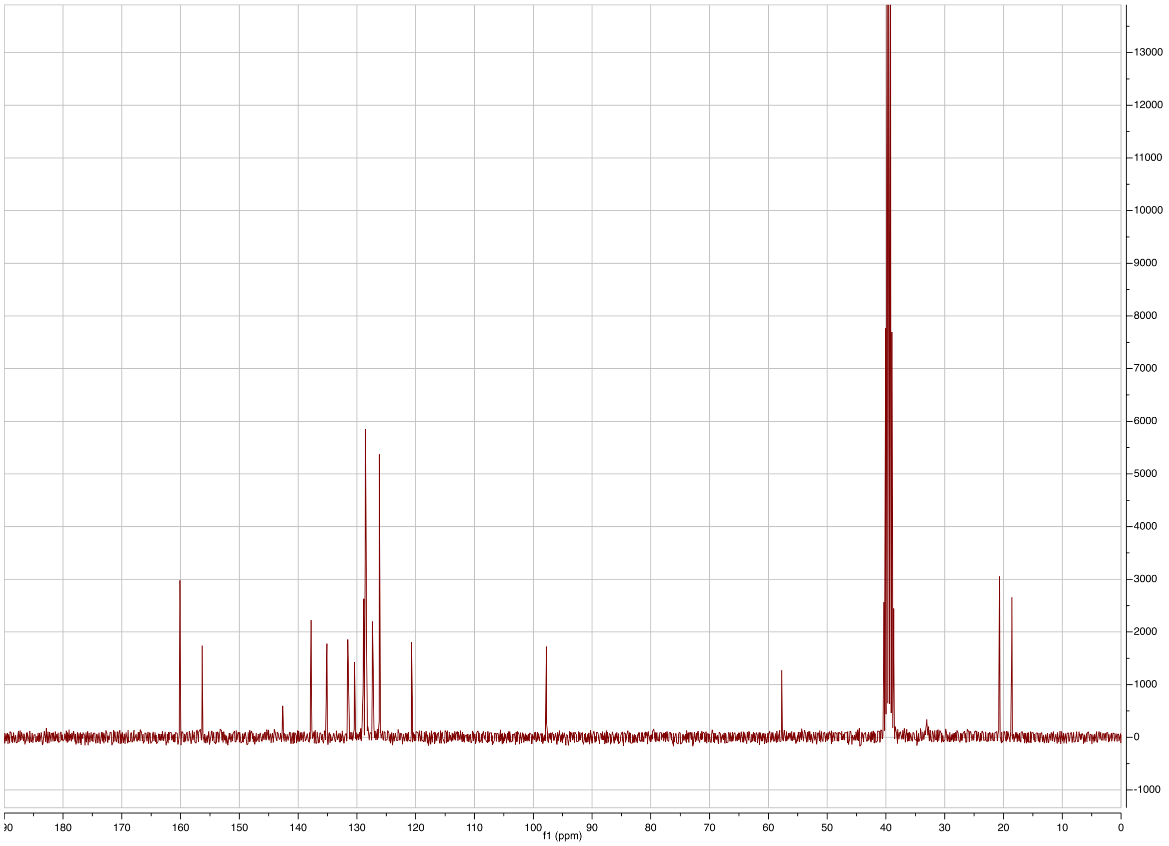
f1 (ppm)

6-amino-3,4-diphenyl-1,4-dihydropyrano[2,3-*c*]pyrazole-5-carbonitrile (10h)

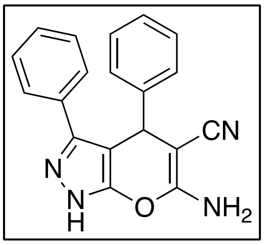

UPLC-UV: Rt = 1.56;
UPLC-MS (ESI) calculated for $C_{19}H_{15}N_4O$ $[M + H]^-$: *m/z* = 315.1, found *m/z* = 315.1;
^{1}H NMR (300 MHz, DMSO-d_6): δ = 12.84 (s, 1H, ArN**H**), 7.44 (d, *J* = 7.1 Hz, 2H, Ar**H**), 7.33 – 7.24 (m, 3H, Ar**H**), 7.22 – 7.17 (m, 2H, Ar**H**), 7.13 – 7.07 (m, 3H, Ar**H**), 6.93 (s, 2H + ArN**H**$_2$), 4.97 (s, 1H, C**H**Ar);
^{13}C NMR (75 MHz, DMSO-d_6): δ = 160.05, 156.03, 144.69, 137.87, 128.63, 128.57, 128.34, 128.31, 127.31, 126.63, 126.20, 120.63, 97.42, 58.26, 36.74.

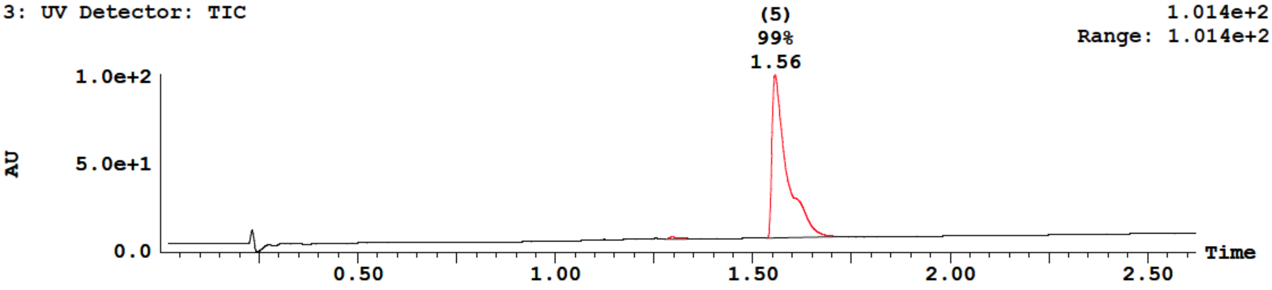

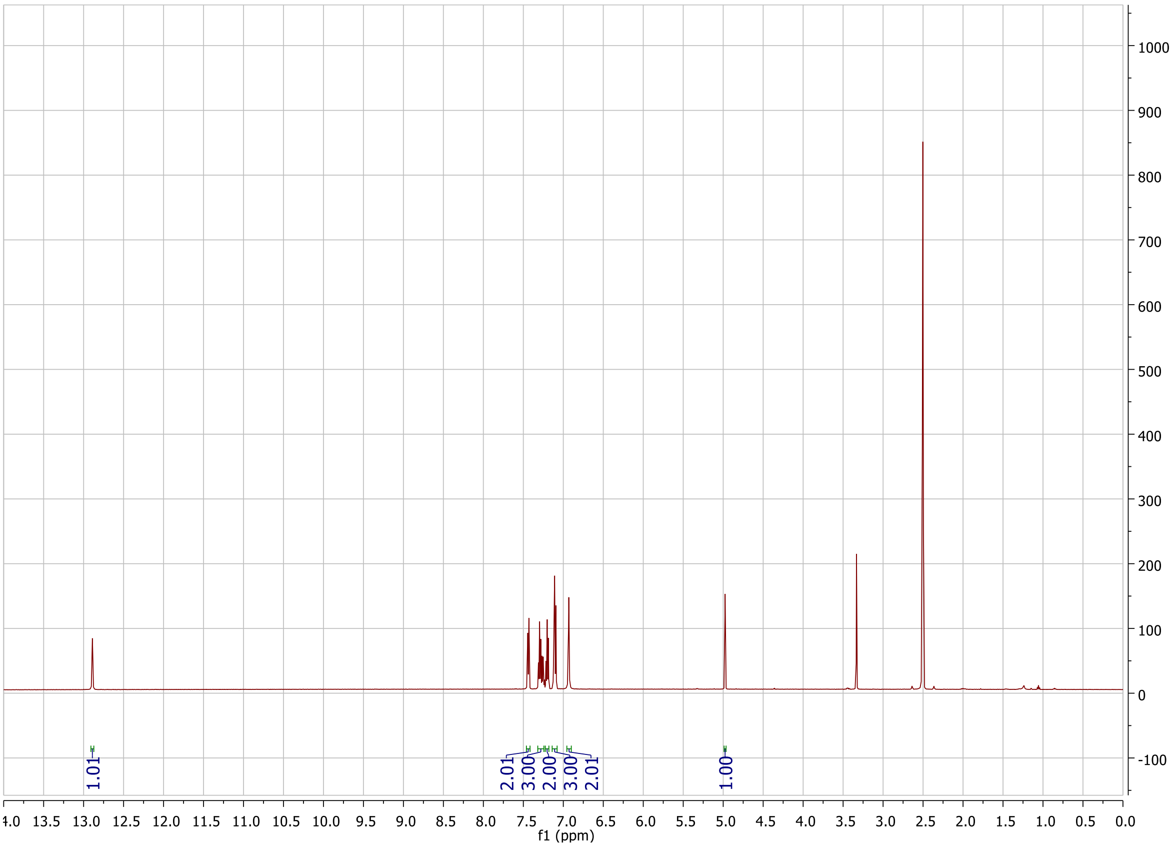

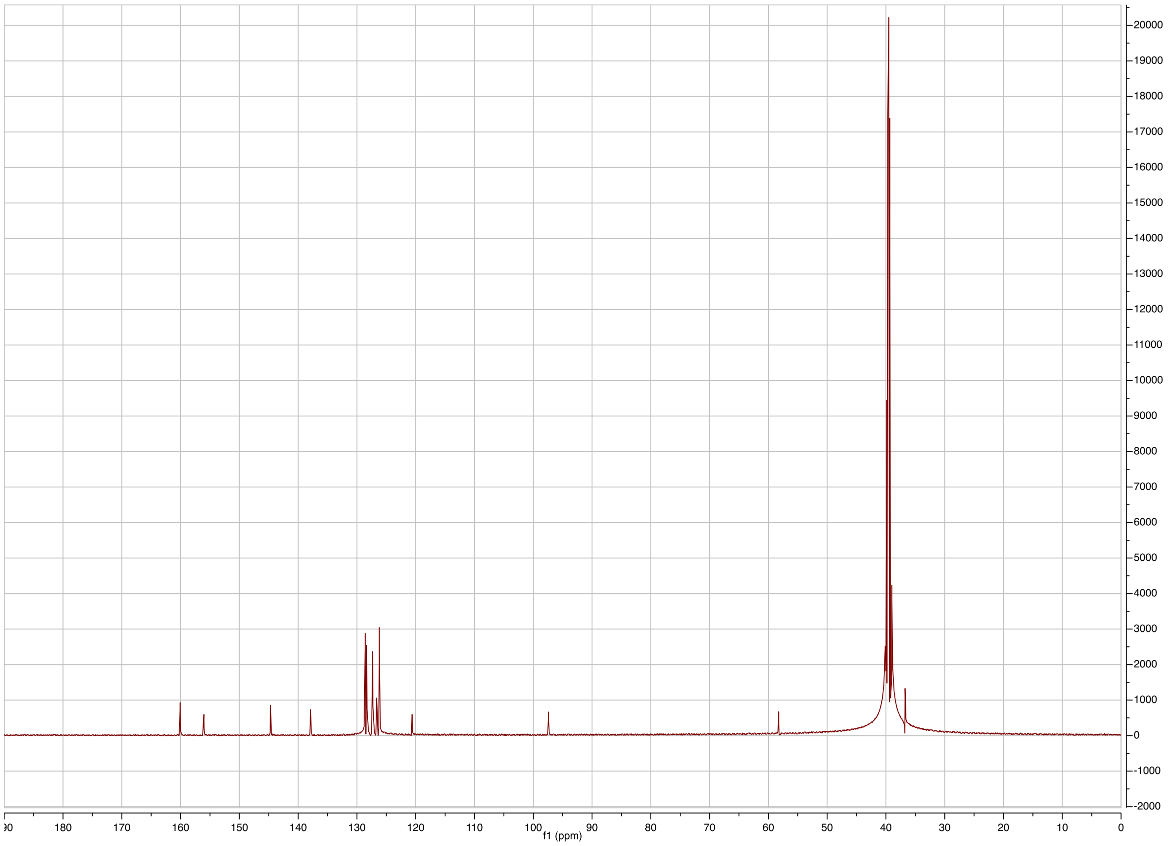
f1 (ppm)

6-amino-4-(2,4-dimethylphenyl)-3-phenyl-1,4-dihydropyrano[2,3-*c*]pyrazole-5-carbonitrile (10i)

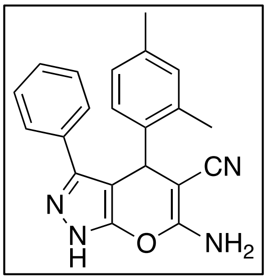

UPLC-UV: Rt = 1.74, 1.80 (2,4-dihydro tautomer);
UPLC-MS (ESI) calculated for $C_{21}H_{19}N_4O$ $[M + H]^{-}$: *m/z* = 343.2, found *m/z* = 343.2;
^{1}H NMR (400 MHz, DMSO-d_6): δ = 12.83 (s, 1H, ArN**H**), 7.64 – 7.09 (m, 5H, Ar**H**), 6.89 – 6.81 (m, 4H, 2 × Ar**H** + 2 × ArN**H**$_2$), 6.77 (d, *J* = 7.9, 1H, Ar**H**), 5.15 (s, 1H, C**H**Ar), 2.30 (s, 3H, ArC**H**$_3$), 2.15 (s, 3H, ArC**H**$_3$);
^{13}C NMR (100 MHz, DMSO-d_6): δ = 159.98, 156.26, 137.70, 135.27, 134.30, 130.99, 128.79, 128.51 (two overlapping signals), 128.27, 127.01, 126.07 (two overlapping signals), 120.63, 97.78, 57.81, 20.46 (two overlapping signals), 18.81.

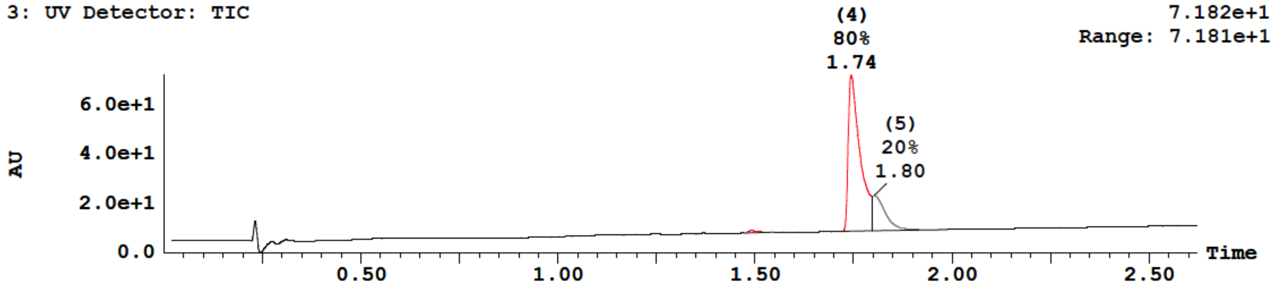

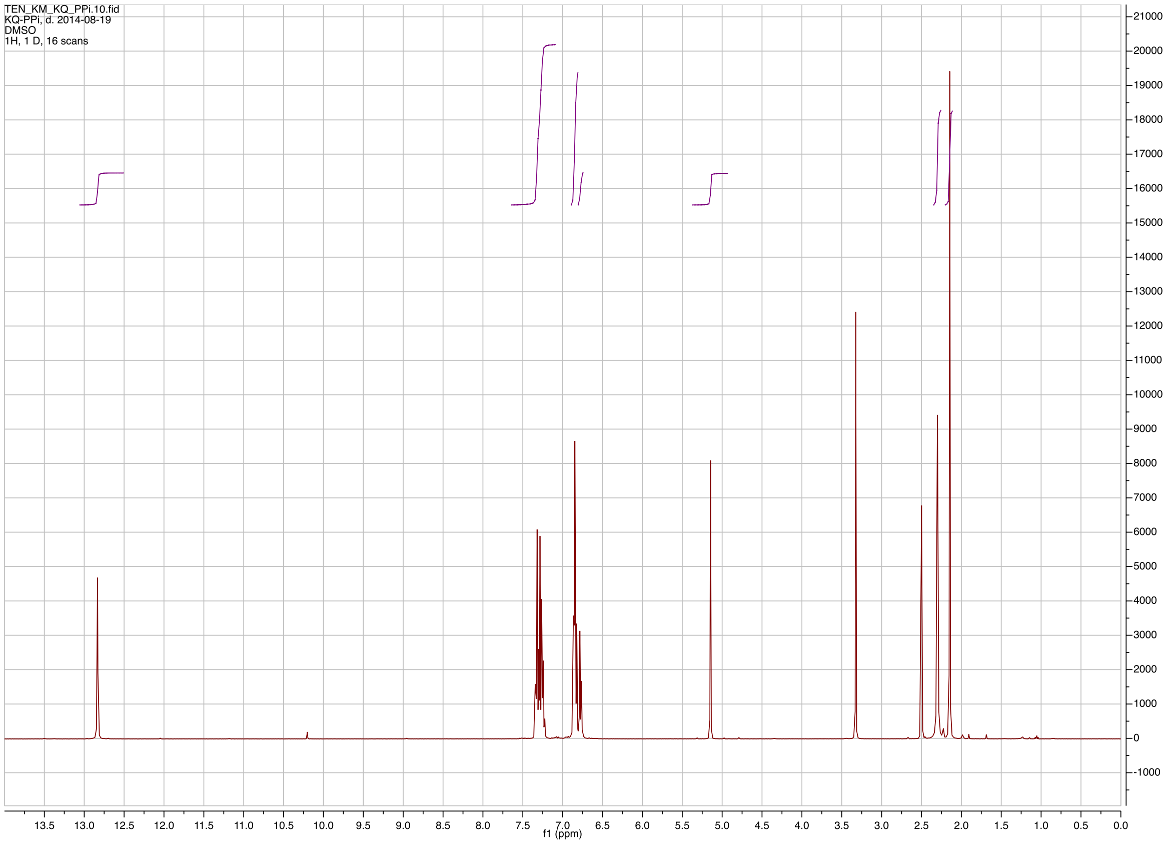

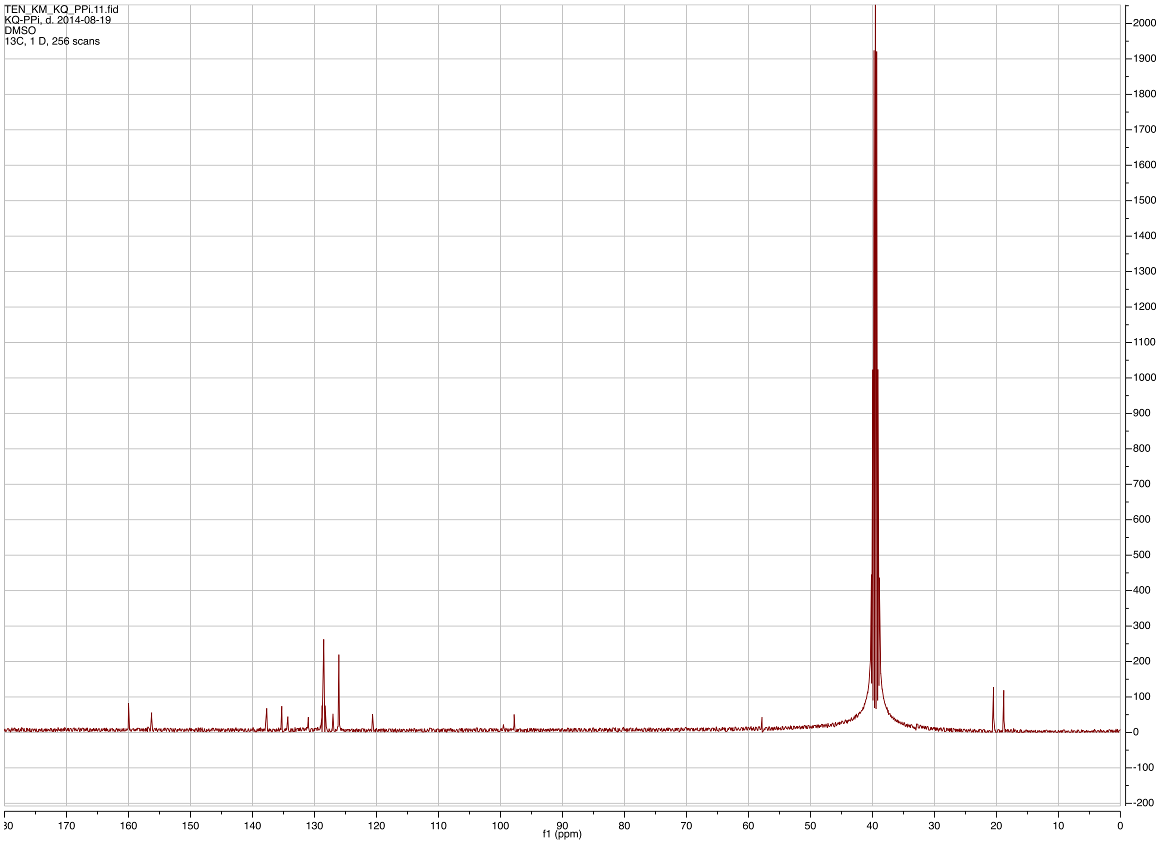

TEN_KM_KQ_PPi.11.fid
KQ-PPi, d. 2014-08-19
DMSO
13C, 1 D, 256 scans
f1 (ppm)

6-amino-4-(4-methoxy-2,3-dimethylphenyl)-3-phenyl-1,4-dihydropyrano[2,3-*c*]pyrazole-5-carbonitrile (10j)

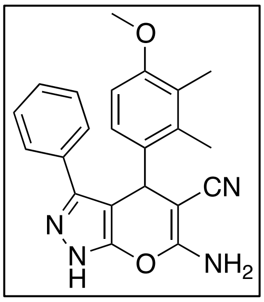

UPLC-UV: Rt = 1.73, 1.79 (2,4-dihydro tautomer);
UPLC-MS (ESI) calculated for $C_{22}H_{21}N_4O_2$ $[M + H]^-$: *m/z* = 373.2, found *m/z* = 373.2;
^{1}H NMR (300 MHz, DMSO-*d*$_6$): δ = 12.84 (s, 1H, ArN**H**), 7.94 – 7.01 (m, 5H, Ar**H**), 6.78 – 6.72 (m, 4H, 2 × Ar**H** + ArN**H$_2$**), 5.23 (s, 1H, C**H**Ar), 3.65 (s, 3H, ArOC**H$_3$**), 2.26 (s, 3H, ArC**H$_3$**), 2.05 (s, 3H, ArC**H$_3$**);
^{13}C NMR (75 MHz, DMSO-*d*$_6$): δ = 159.87, 156.29, 155.25, 137.56, 134.50, 128.84, 128.51, 128.26, 126.74, 126.10, 123.54, 120.73, 109.81, 108.46, 98.46, 58.54, 55.17, 15.06, 12.06 (two overlapping signals).

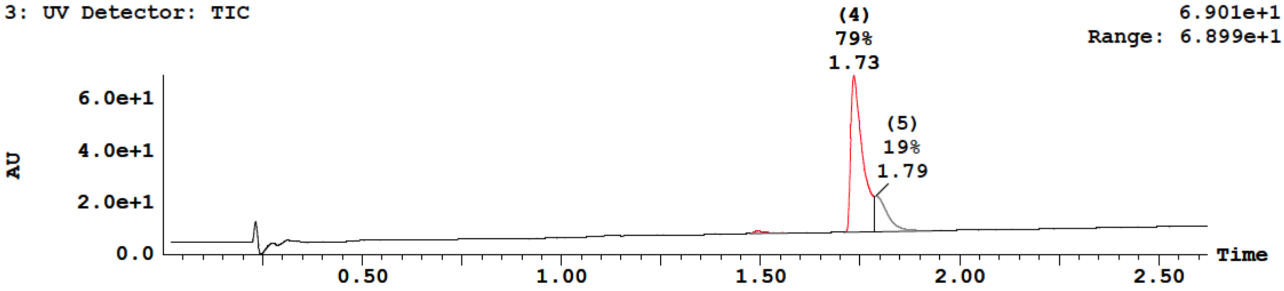

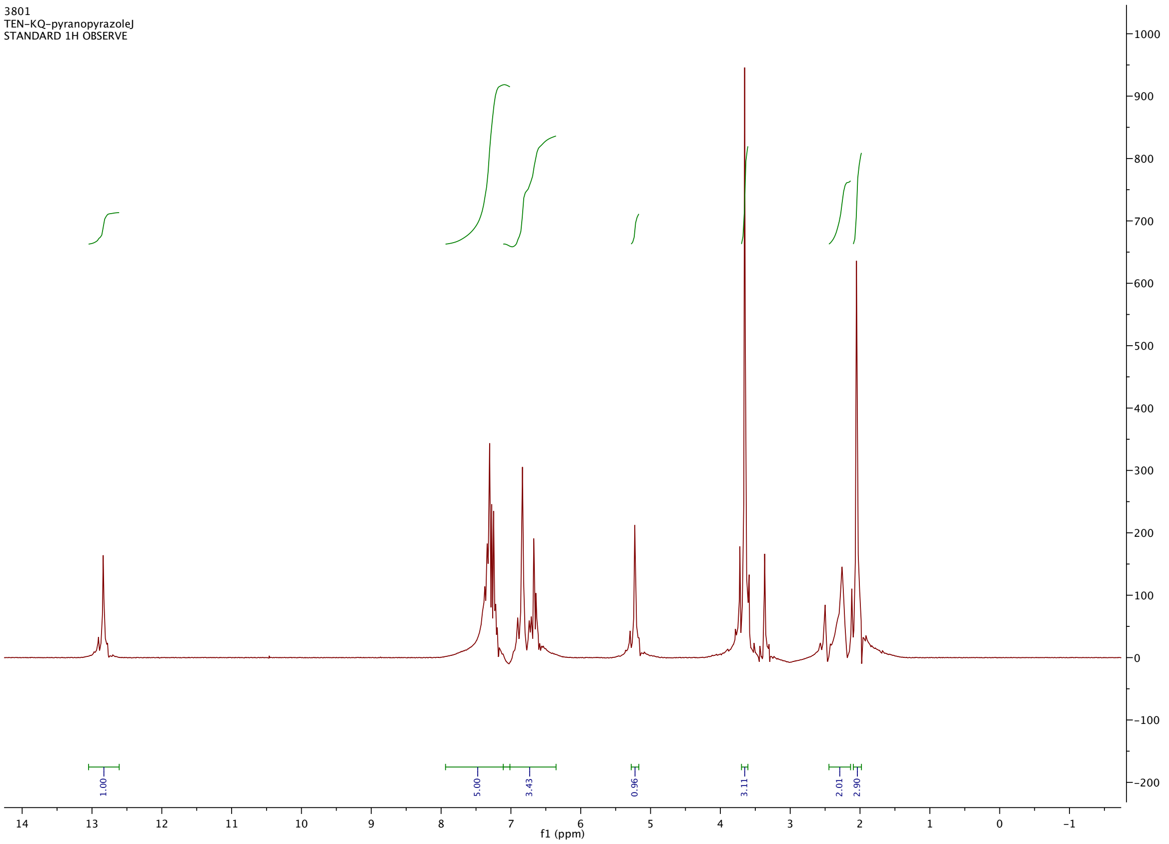

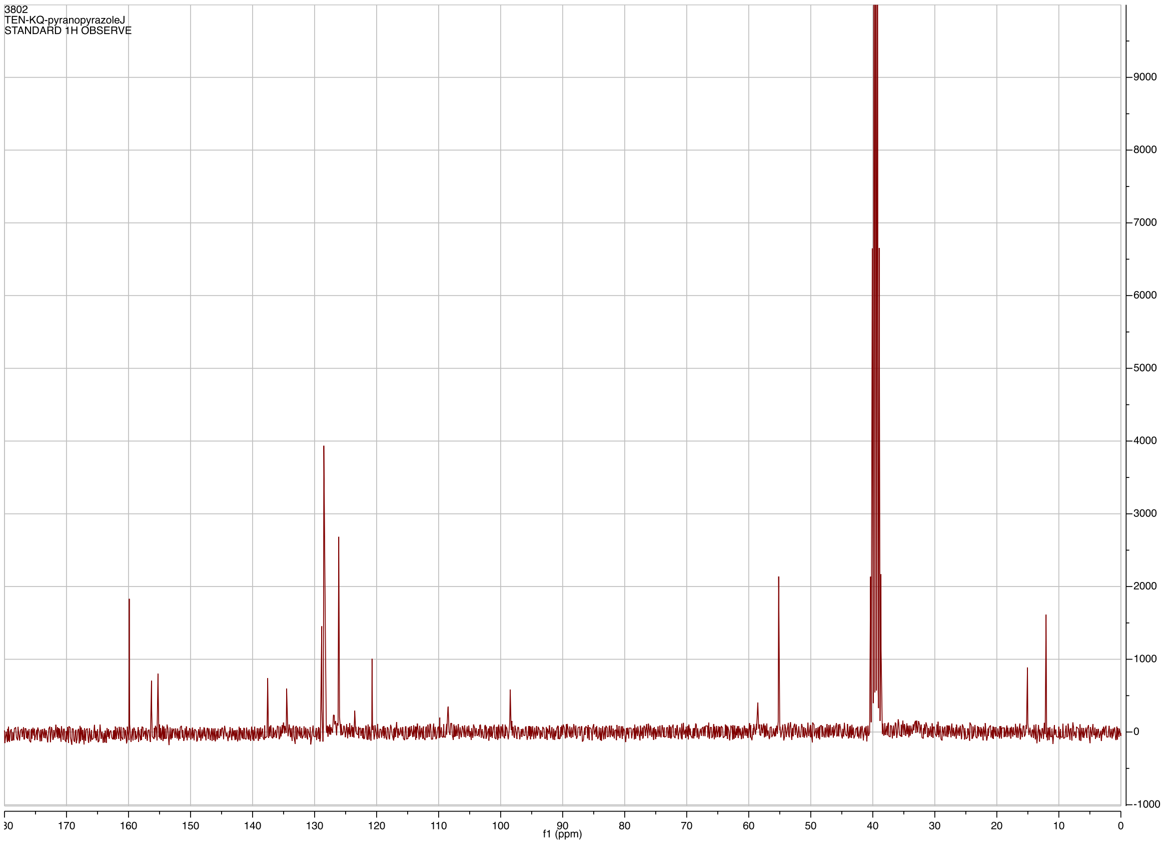
3802
TEN-KQ-pyranopyrazoleJ
STANDARD 1H OBSERVE
f1 (ppm)

6-amino-4-(4-fluorophenyl)-3-phenyl-1,4-dihydropyrano[2,3-*c*]pyrazole-5-carbonitrile (10k)

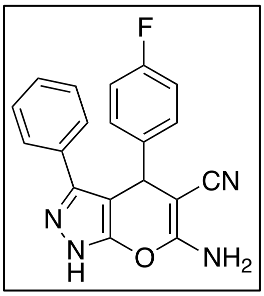

UPLC-UV: Rt = 1.58;
UPLC-MS (ESI) calculated for $C_{19}H_{14}FN_4O$ $[M + H]^-$: *m/z* = 333.1, found *m/z* = 333.1;
^{1}H NMR (400 MHz, DMSO-d_6): δ = 12.90 (s, 1H, ArN**H**), 7.48 – 7.39 (m, 2H, 2 × Ar**H**), 7.34 – 7.22 (m, 3H, 3 × Ar**H**), 7.17 – 7.09 (m, 2H, 2 × Ar**H**), 7.06 – 6.97 (m, 2H, 2 × Ar**H**), 6.94 (s, 2H, ArN**H₂**), 5.04 (s, 1H, C**H**Ar);
^{13}C NMR (100 MHz, DMSO-d_6): δ = 160.77 (d, *J* = 241 Hz), 160.02, 155.88, 140.80 (d, *J* = 3 Hz), 137.98, 129.20 (d, *J* = 3 Hz), 128.55, 128.52, 128.35, 126.25, 120.52, 115.02 (d, *J* = 21 Hz), 97.27, 58.09, 35.91.

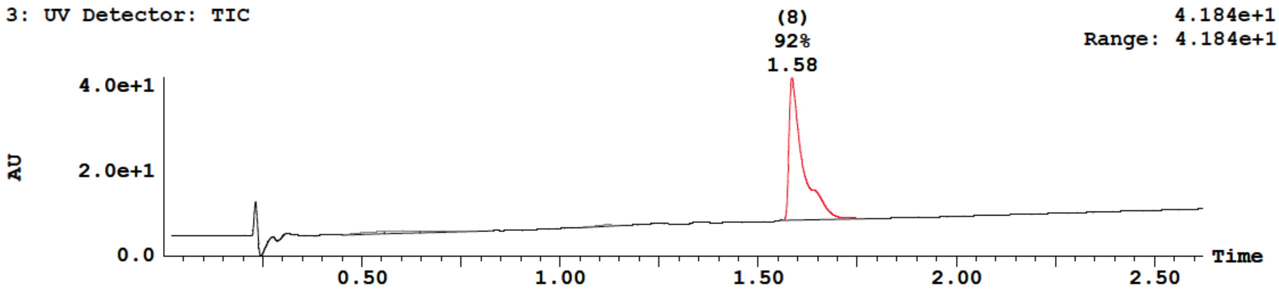

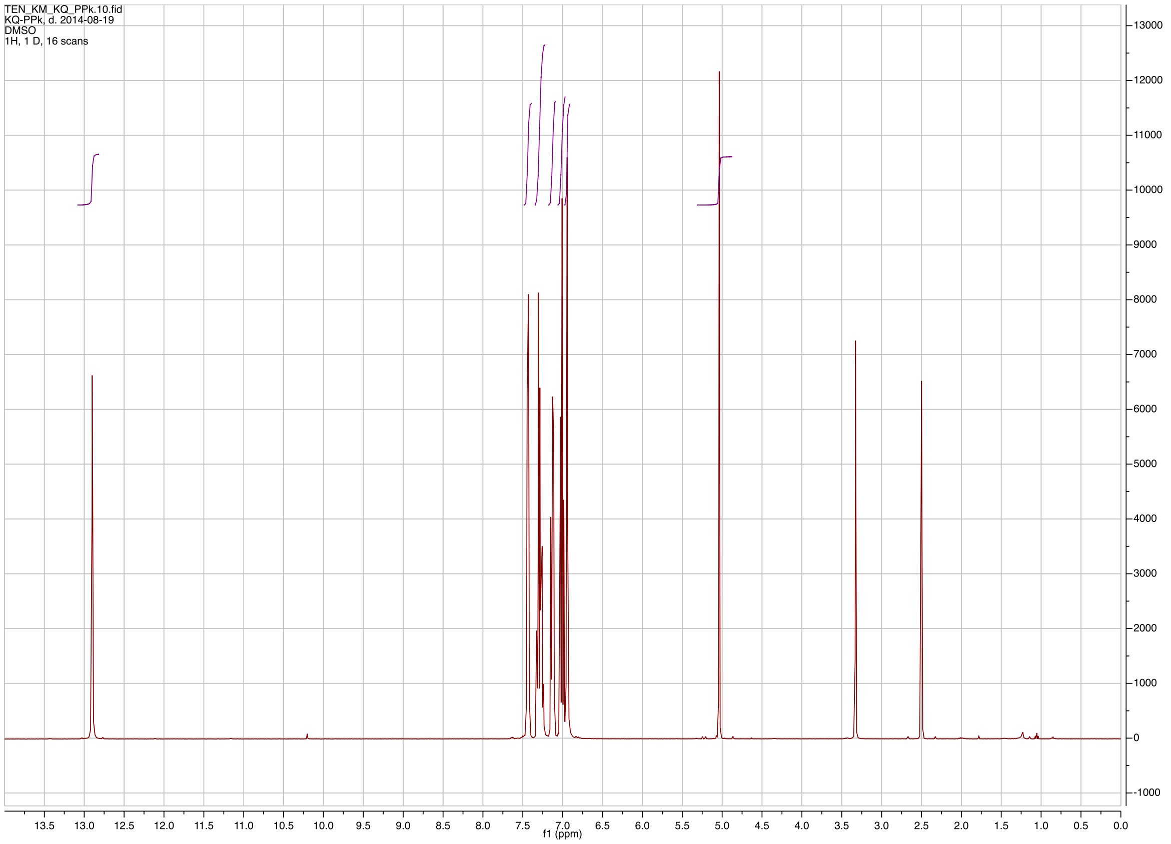

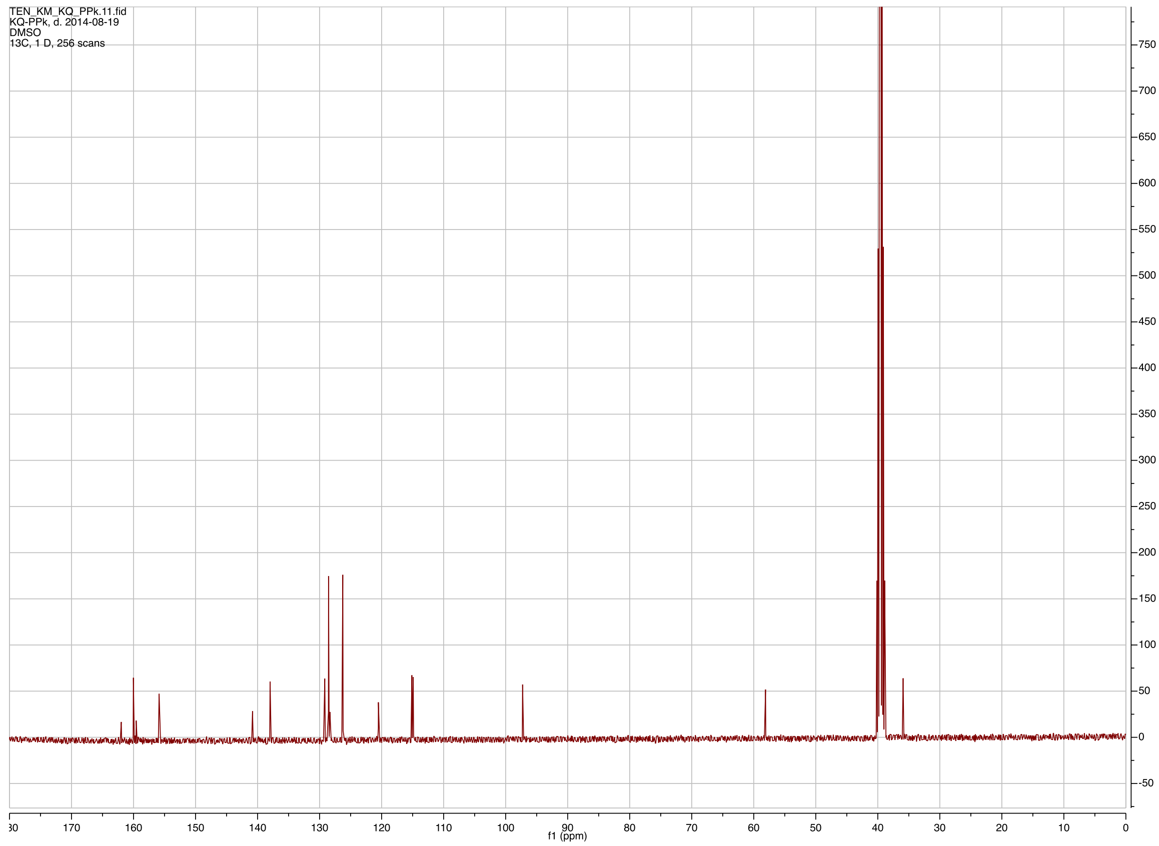
TEN_KM_KQ_PPk.11.fid
KQ-PPk, d. 2014-08-19
DMSO
13C, 1 D, 256 scans
f1 (ppm)

6-amino-3-phenyl-4-(*o*-tolyl)-1,4-dihydropyrano[2,3-*c*]pyrazole-5-carbonitrile (10l)

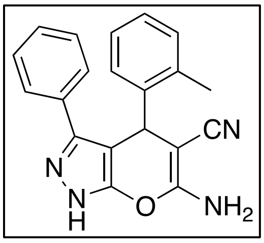

UPLC-UV: Rt = 1.64, 1.69 (2,4-dihydro tautomer);
UPLC-MS (ESI) calculated for $C_{20}H_{17}N_4O$ $[M + H]^-$: *m/z* = 329.1, found *m/z* = 329.1;
^{1}H NMR (400 MHz, DMSO-d_6): δ = 12.84 (s, 1H, ArN**H**), 7.33 – 7.20 (m, 6H, 5 × Ar**H**), 7.06 – 6.88 (m, 3H, 3 × Ar**H**), 6.91 (d, *J* = 2.2 Hz, 1H, Ar**H**), 6.88 (s, 2H, ArN**H$_2$**), 5.19 (s, 1H, C**H**Ar), 2.33 (s, 3H, ArC**H$_3$**);
^{13}C NMR (100 MHz, DMSO-d_6): δ = 160.09, 156.23, 142.67, 137.84, 134.59, 130.36, 128.75, 128.59, 128.46, 128.30, 126.46, 126.31, 126.14, 120.59, 97.70, 57.55, 33.20, 18.88.

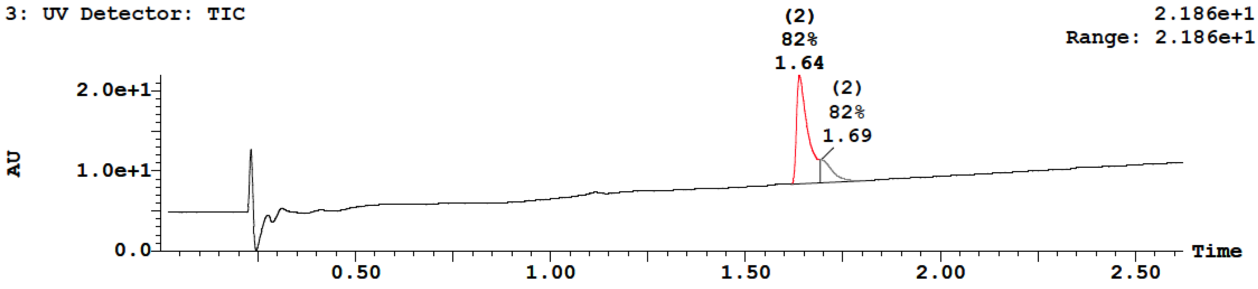

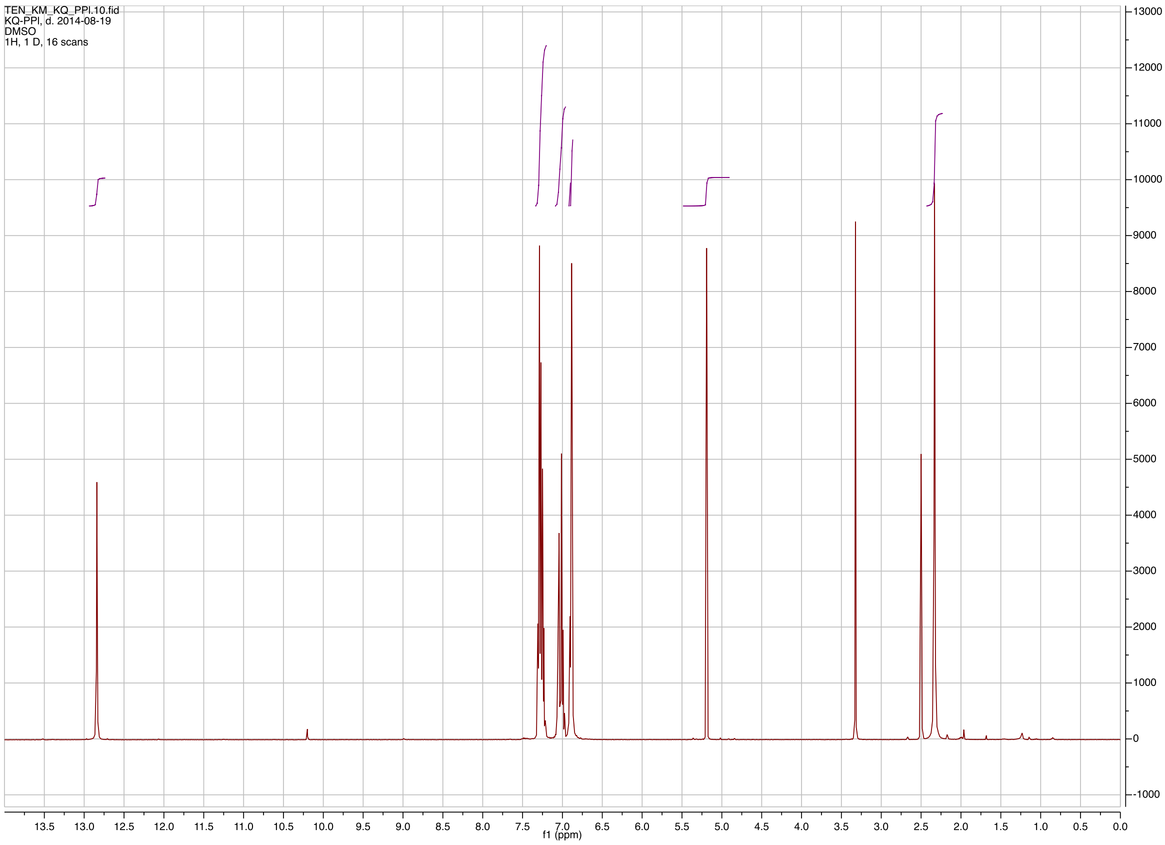

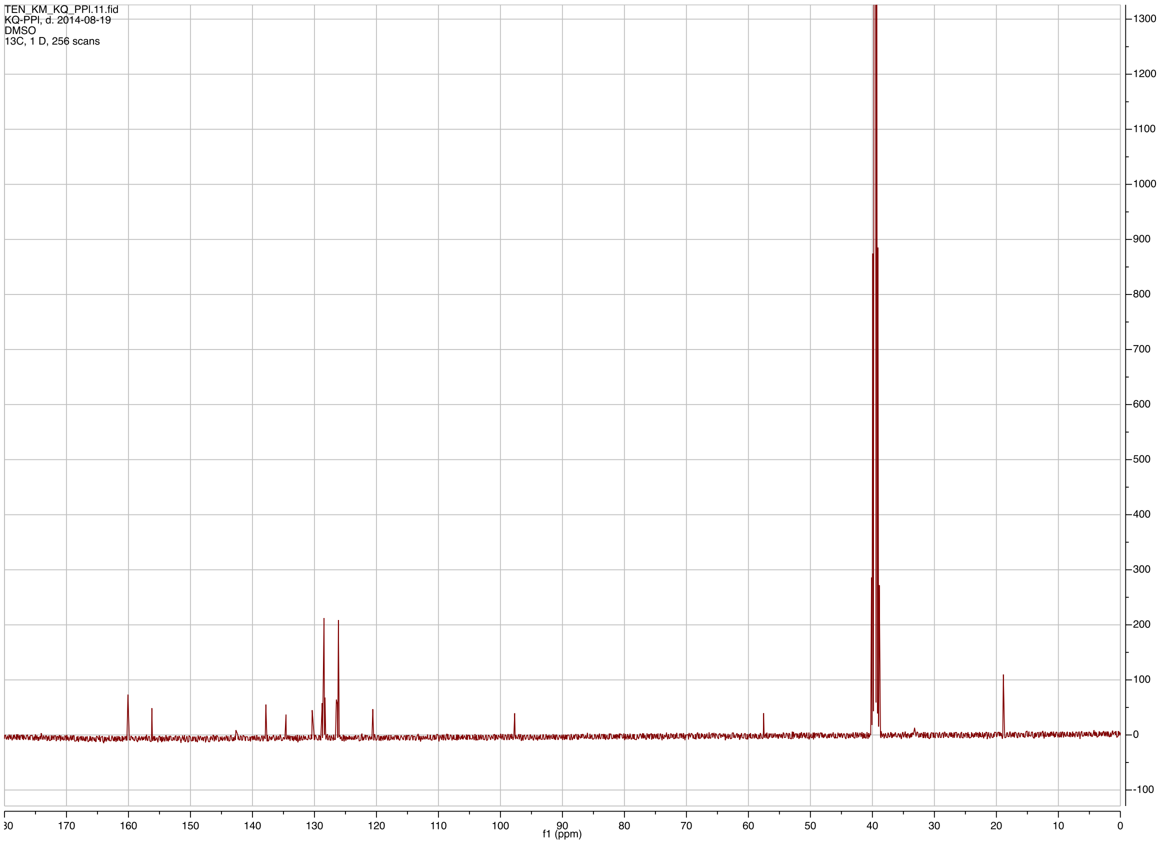
TEN_KM_KQ_PPI.11.fid
KQ-PPI, d. 2014-08-19
DMSO
13C, 1 D, 256 scans
f1 (ppm)

6-amino-4-(2,4-dimethylphenyl)-3-propyl-1,4-dihydropyrano[2,3-*c*]pyrazole-5-carbonitrile (10m)

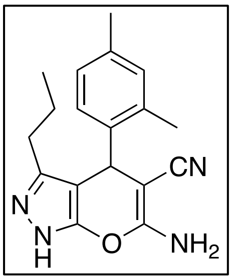

UPLC: Rt = 1.71, 1.77 (2,4-dihydro tautomer);
UPLC-MS (ESI) calculated for $C_{18}H_{21}N_4O$ $[M + H]^-$: *m/z* = 309.2, found *m/z* = 309.2;
^{1}H NMR (300 MHz, DMSO-d_6): δ = 12.07 (s, 1H, ArN**H**), 6.96 – 6.85 (m, 3H, Ar**H**), 6.80 (s, 2H, ArN**H**$_2$), 4.83 (s, 1H, C**H**Ar), 2.20 (s, 6H, 2 × ArC**H**$_3$), 2.17 – 1.88 (m, 2H, C**H**$_2$), 1.29 – 0.91 (m, 2H, C**H**$_2$), 0.57 (t, *J* = 7.3 Hz, 3H, C**H**$_3$);
^{13}C NMR (75 MHz, DMSO-d_6): δ = 160.59, 155.00, 139.54, 139.20, 135.51, 134.73, 131.21, 129.03, 126.86, 120.78, 97.20, 56.94, 26.28, 20.98, 20.57, 18.78, 13.36 (two overlapping aliphatic signals).

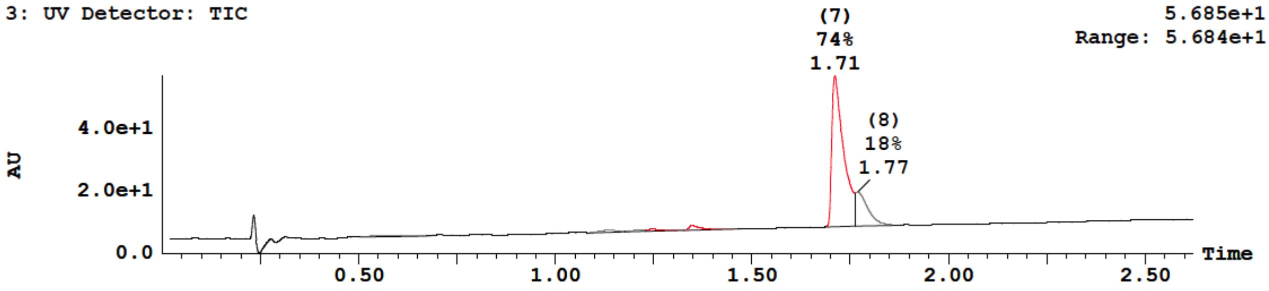

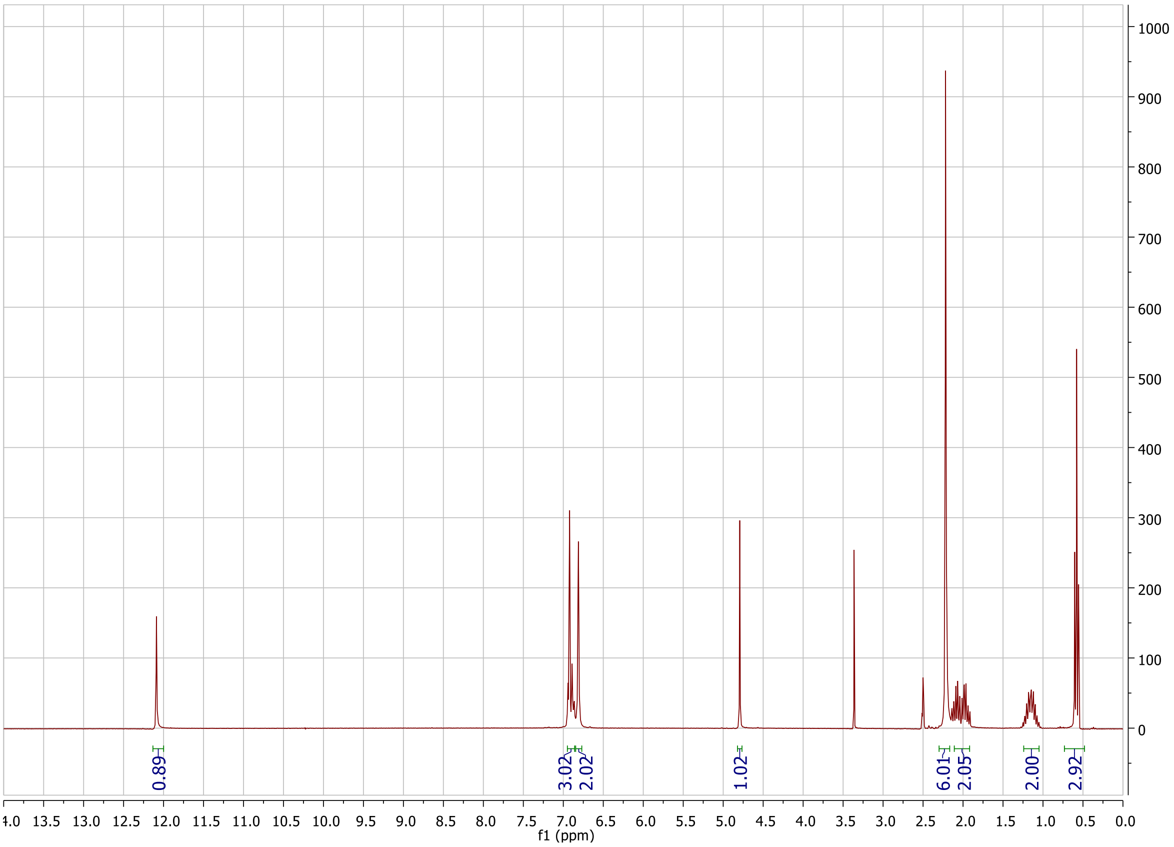

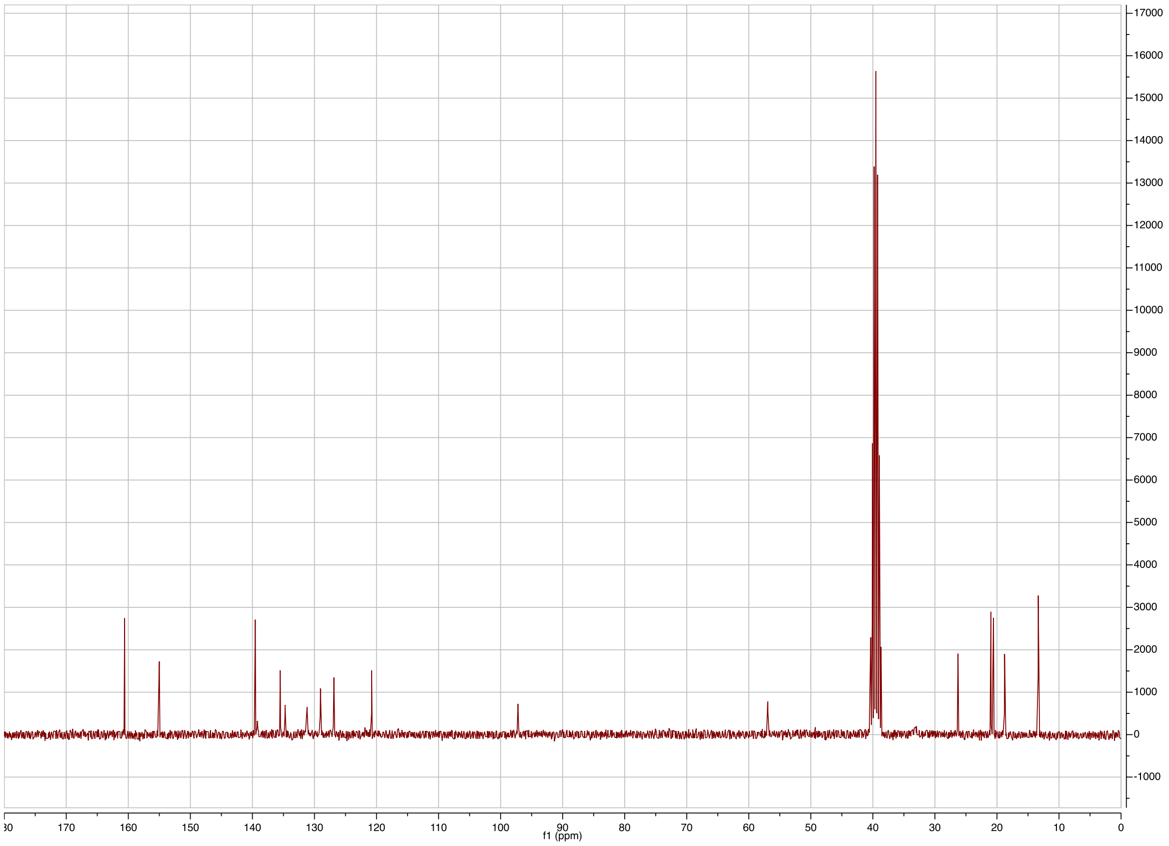
f1 (ppm)

6-amino-4-(4-chlorophenyl)-3-propyl-1,4-dihydropyrano[2,3-*c*]pyrazole-5-carbonitrile (10n)

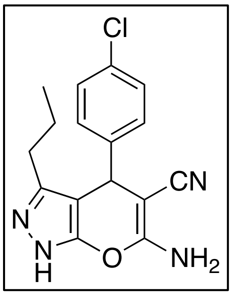

UPLC: Rt = 1.66, 1.71 (2,4-dihydro tautomer);
UPLC-MS (ESI) calculated for $C_{16}H_{16}ClN_4O$ [M + H]$^-$: *m/z* = 315.1 (^{35}Cl), 317.1 (^{37}Cl) found *m/z* = 315.1 (100 %), 317.1 (30 %);
^{1}H NMR (400 MHz, DMSO-d_6): δ = 12.15 (s, 1H, ArN**H**), 7.37 (d, J = 8.4 Hz, 2H, 2 × Ar**H**), 7.19 (d, J = 8.4 Hz, 2H, 2 × Ar**H**), 6.91 (s, 2H, ArN**H$_2$**), 4.63 (s, 1H, C**H**Ar), 2.17 (ddd, *J* = 15.0, 8.5, 6.7 Hz, 1H, ArC**H**HCH$_2$), 2.05 (ddd, *J* = 14.7, 8.5, 6.4 Hz, 1H, ArC**H**HCH$_2$), 1.33-1.06 (m, 2H, CH$_2$C**H$_2$**CH$_3$), 0.63 (t, *J* = 7.3 Hz, 3H CH$_2$C**H$_3$**);
^{13}C NMR (100 MHz, DMSO-d_6): δ = 160.75, 154.59, 143.91, 139.82, 131.23, 129.41, 128.40, 120.63, 96.90, 56.97, 35.72, 26.19, 20.95, 13.31.

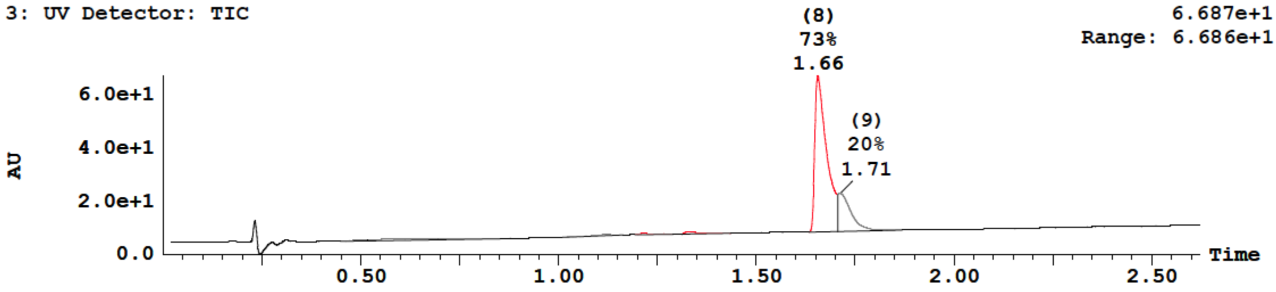

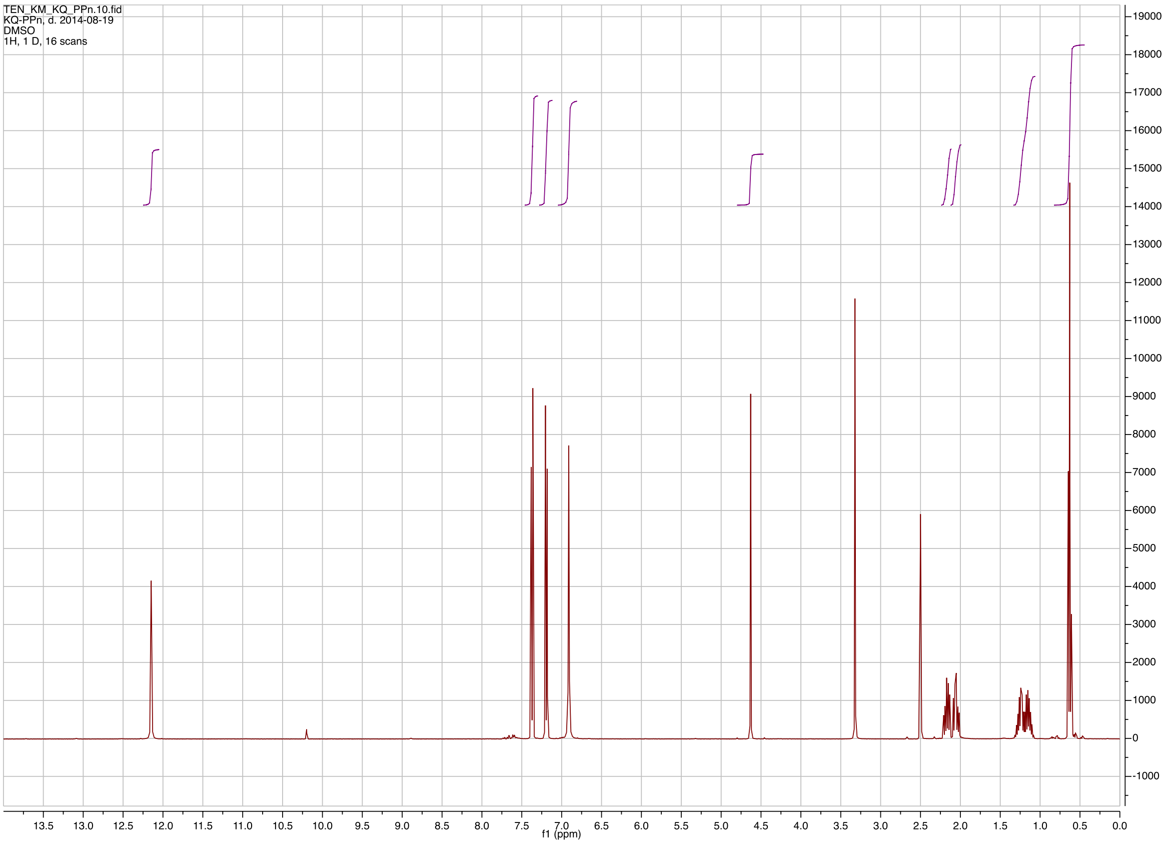

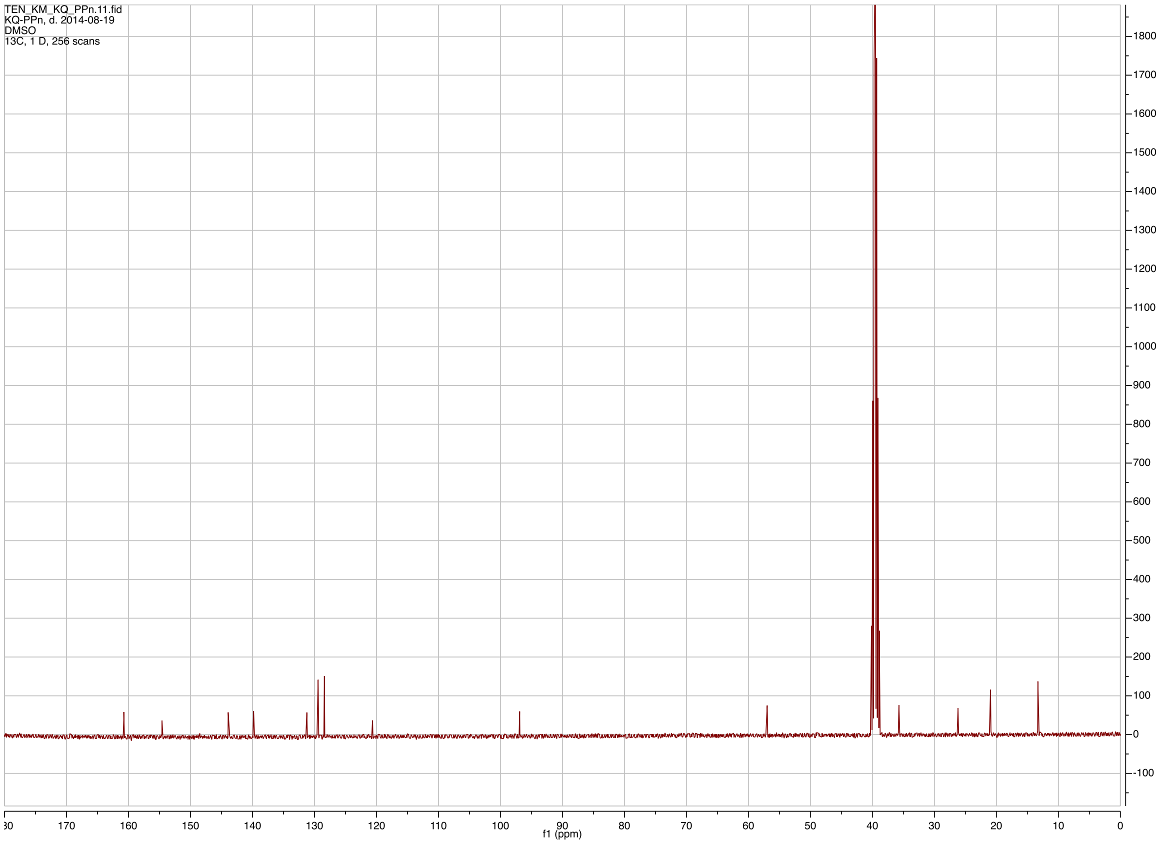

TEN_KM_KQ_PPn.11.fid
KQ-PPn, d. 2014-08-19
DMSO
13C, 1 D, 256 scans
f1 (ppm)

6-amino-4-phenyl-3-propyl-1,4-dihydropyrano[2,3-*c*]pyrazole-5-carbonitrile (10o)

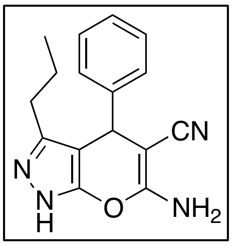

UPLC: Rt = 1.50, 1.56 (2,4-dihydro tautomer);
UPLC-MS (ESI) calculated for $C_{16}H_{17}N_4O$ $[M + H]^-$: *m/z* = 281.1, found *m/z* = 281.1;
^{1}H NMR (400 MHz, DMSO-d_6): δ = 12.10 (s, 1H, ArN**H**), 7.36-7.27 (m, 2H, 2 × Ar**H**), 7.25 – 7.19 (m, 1H, Ar**H**), 7.18 – 7.13 (m, 2H, 2 × Ar**H**), 6.85 (s, 2H, ArN**H₂**), 4.58 (s, 1H, C**H**Ar), 2.16 (ddd, *J* = 14.9, 8.6, 6.6 Hz, 1H, ArC**H**HCH$_2$), 2.04 (ddd, *J* = 14.7, 8.6, 6.4 Hz, 1H, ArC**H**HCH$_2$), 1.30-1.03 (m, 2H, CH$_2$C**H**$_2$CH$_3$), 0.61 (t, *J* = 7.3 Hz, 3H CH$_2$C**H**$_3$);
^{13}C NMR (100 MHz, DMSO-d_6): δ = 160.71, 154.63, 144.87, 139.76, 128.39, 127.52, 126.75, 120.77, 97.33, 57.39, 36.44, 26.21, 20.93, 13.31.

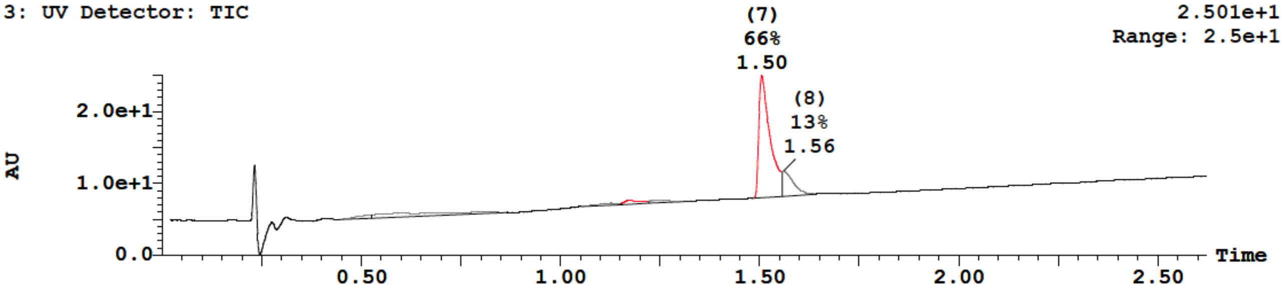

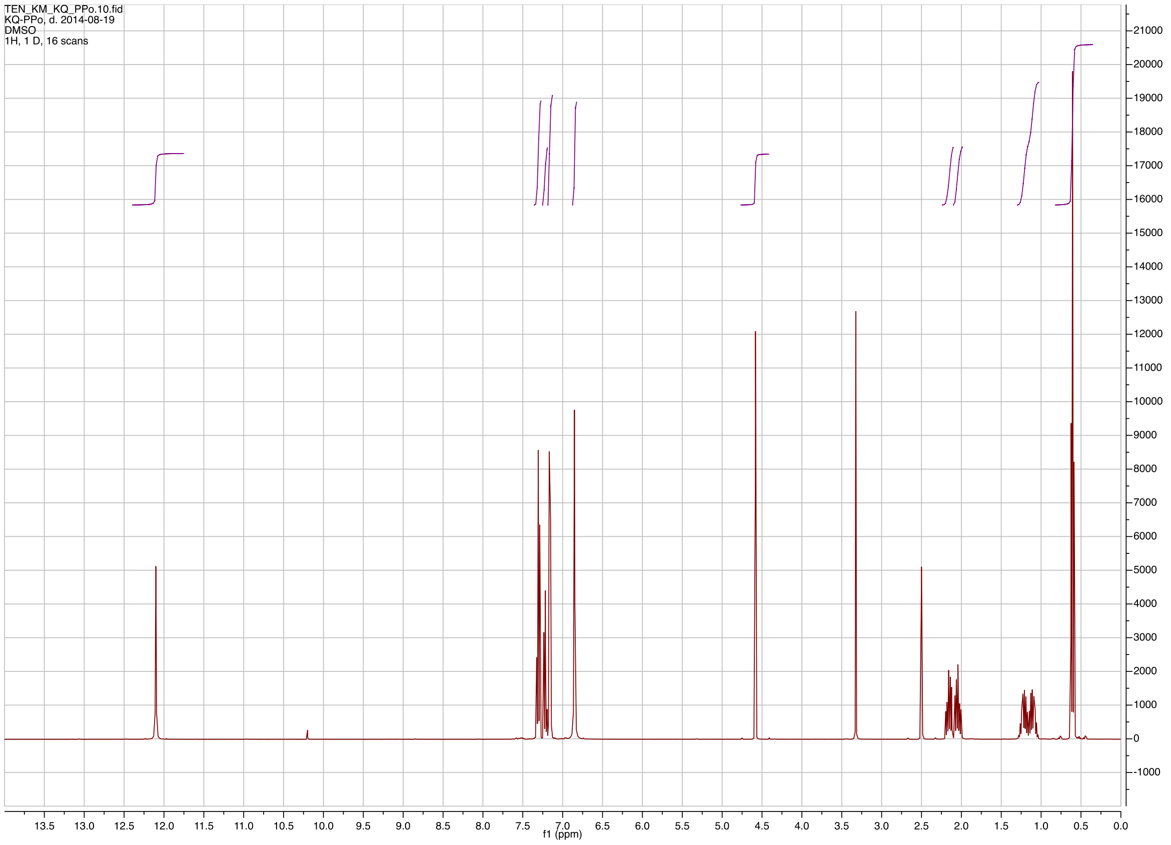

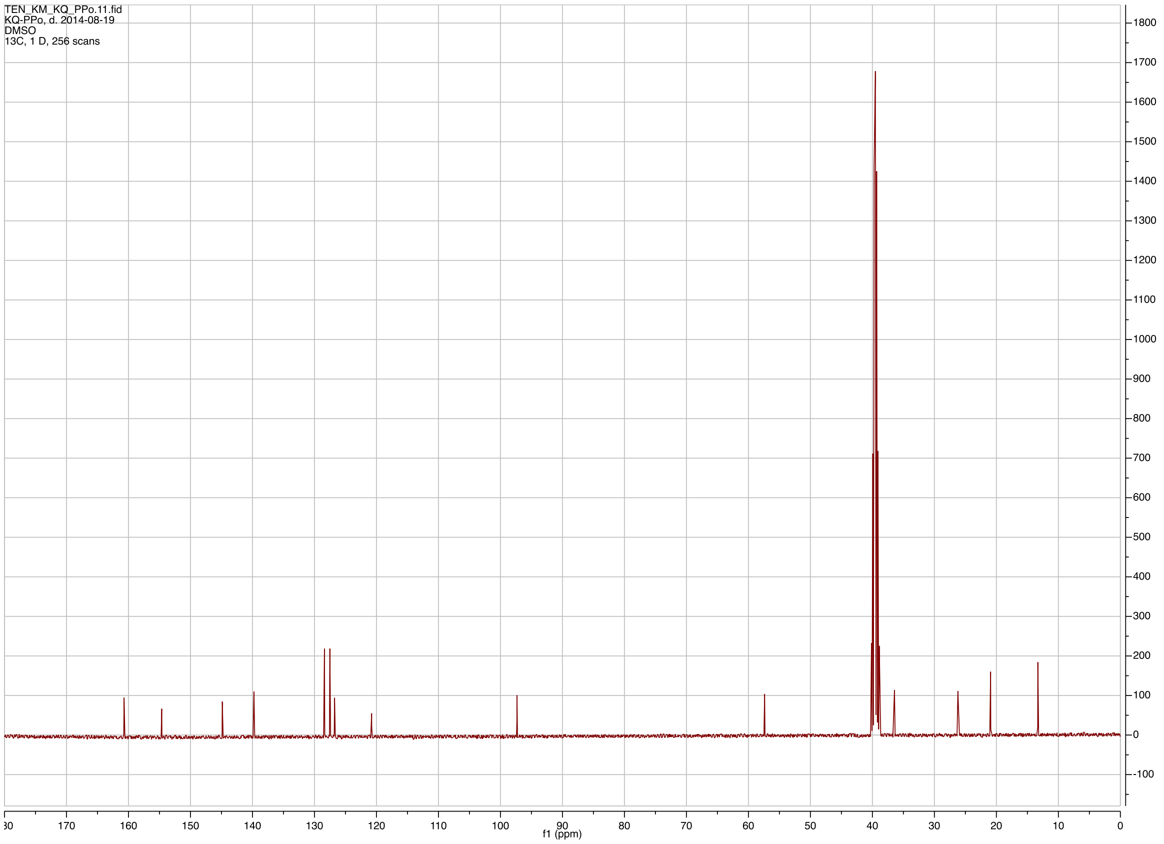
TEN_KM_KQ_PPo.11.fid
KQ-PPo, d. 2014-08-19
DMSO
13C, 1 D, 256 scans
f1 (ppm)

6-amino-4-(2,5-dimethylphenyl)-3-propyl-1,4-dihydropyrano[2,3-*c*]pyrazole-5-carbonitrile (10p)

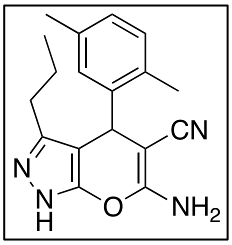

UPLC: Rt = 1.69, 1.75 (2,4-dihydro tautomer);
UPLC-MS (ESI) calculated for $C_{18}H_{21}N_4O$ $[M + H]^-$: *m/z* = 309.2, found *m/z* = 309.2;
^{1}H NMR (300 MHz, DMSO-*d*$_6$): δ = 12.09 (s, 1H, ArN**H**), 7.35-6.45 (m, 5H, 3 × Ar**H** + ArN**H**$_2$), 4.79 (s, 1H, C**H**Ar), 2.20-1.75 (m, 2H, ArCH$_2$), 2.18 (s, 6H, 2 × ArCH$_3$), 1.39-0.83 (m, 2H, CH$_2$C**H**$_2$CH$_3$), 0.53 (t, *J* = 7.7 Hz, 3H, CH$_2$C**H**$_3$);
^{13}C NMR (75 MHz, DMSO-*d*$_6$): δ = 160.65, 155.00, 142.08, 139.57, 135.00, 131.89, 130.50, 129.45, 127.42, 127.35, 120.80, 97.21, 56.84, 26.28, 20.94, 20.66, 18.46, 13.31(two overlapping).

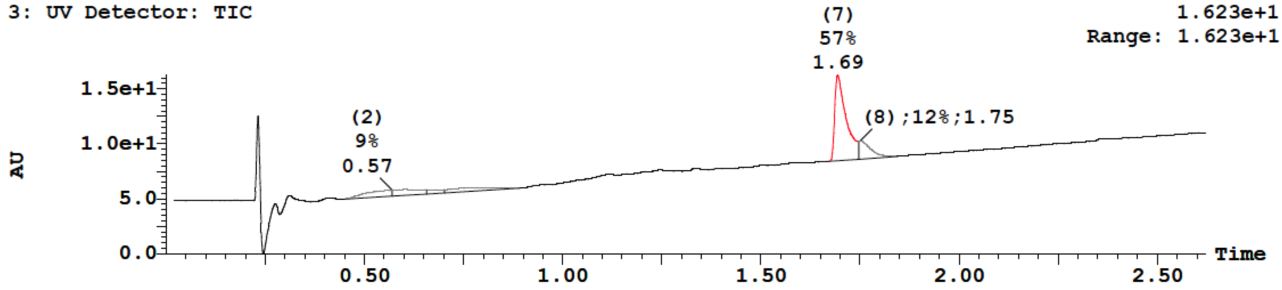

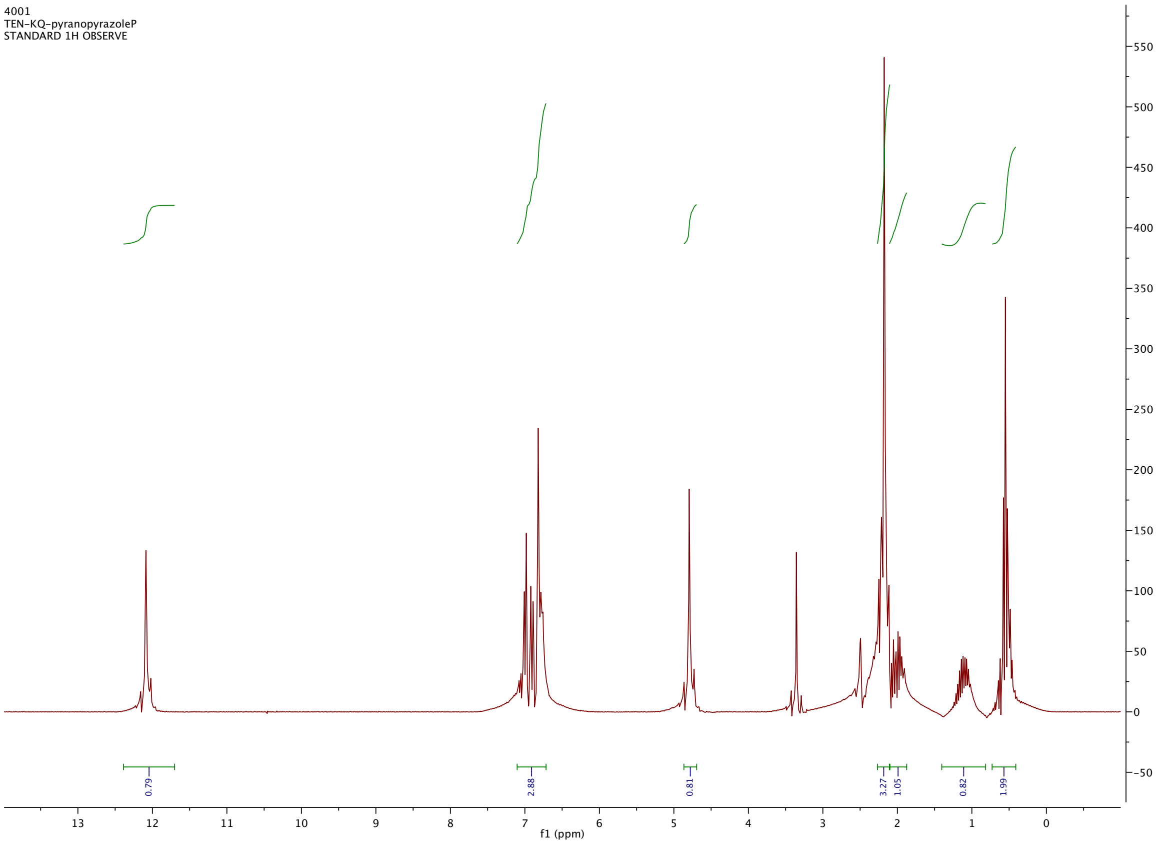

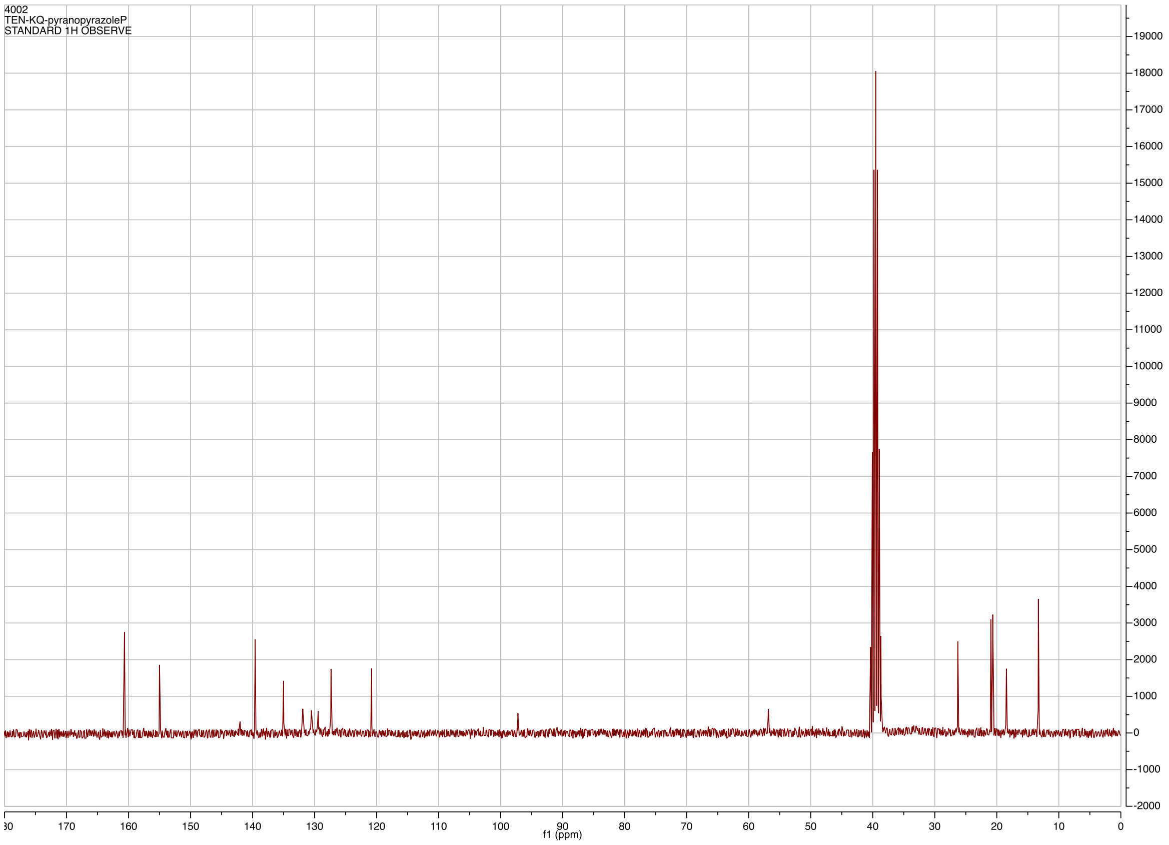
4002
TEN-KQ-pyranopyrazoleP
STANDARD 1H OBSERVE
f1 (ppm)

6-amino-4-(5-((3,4-dichlorophenoxy)methyl)-2,4-dimethylphenyl)-3-methyl-1,4-dihydropyrano[2,3-*c*]pyrazole-5-carbonitrile (10q)

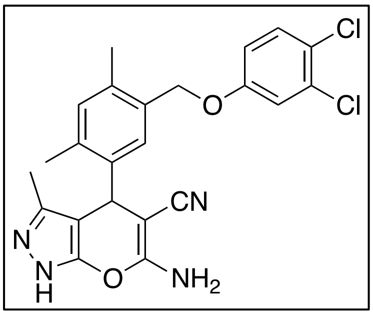

UPLC: Rt = 2.09, 2.15 (2,4-dihydro tautomer);
UPLC-MS (ESI) calculated for $C_{23}H_{20}Cl_2N_4O2$ $[M + H]^{-}$: *m/z* = 455.1 (^{35}Cl), 457.1 (1 x ^{35}Cl, 1 x ^{37}Cl), found *m/z* = 455.1 (100 %), 457.1 (64 %);
^{1}H NMR (300 MHz, DMSO-d_6): δ = 12.06 (s, 1H, ArN**H**), 7.51 (m, 1H, Ar**H**), 7.29 (m, 1H, Ar**H**), 7.00 (m, 3H, ArH), 6.83 (m, 2H, ArN**H**$_2$), 5.02 (s, 2H, ArC**H**$_2$OAr), 4.80 (s, 1H, C**H**Ar), 2.23 (s, 6H, 2 × ArCH$_3$), 1.64 (s, 3H ArCH$_3$);
^{13}C NMR (75 MHz, DMSO-d_6): δ = 160.74, 157.84, 155.06, 135.31, 135.24, 132.54, 132.03, 131.99, 131.55, 130.86, 129.87, 129.83, 122.51, 120.79, 116.77, 115.95, 97.43, 68.93, 56.74, 40.35, 18.56, 18.04 (two overlapping), 9.59.

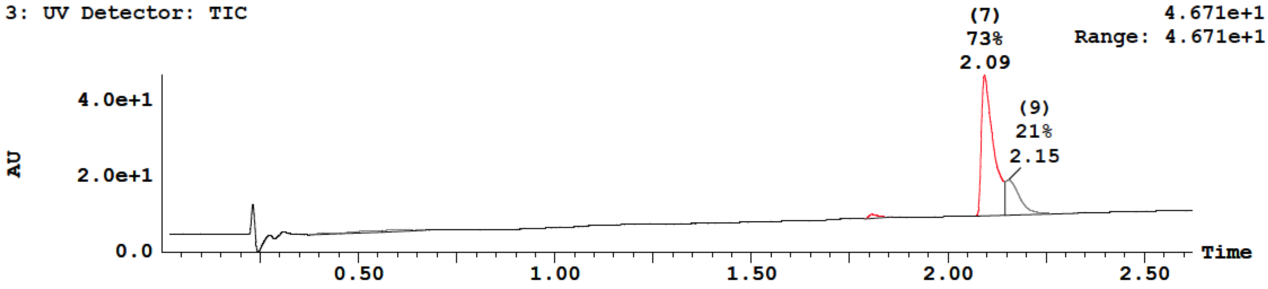

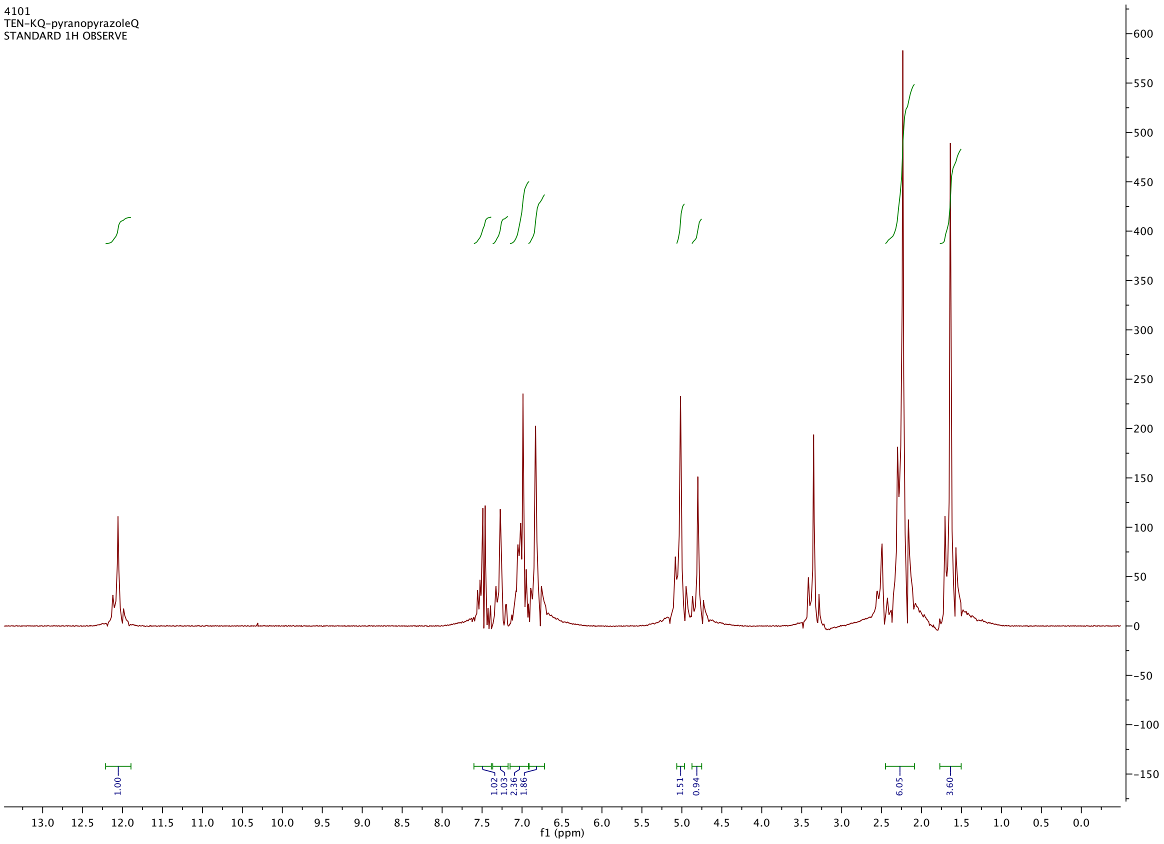

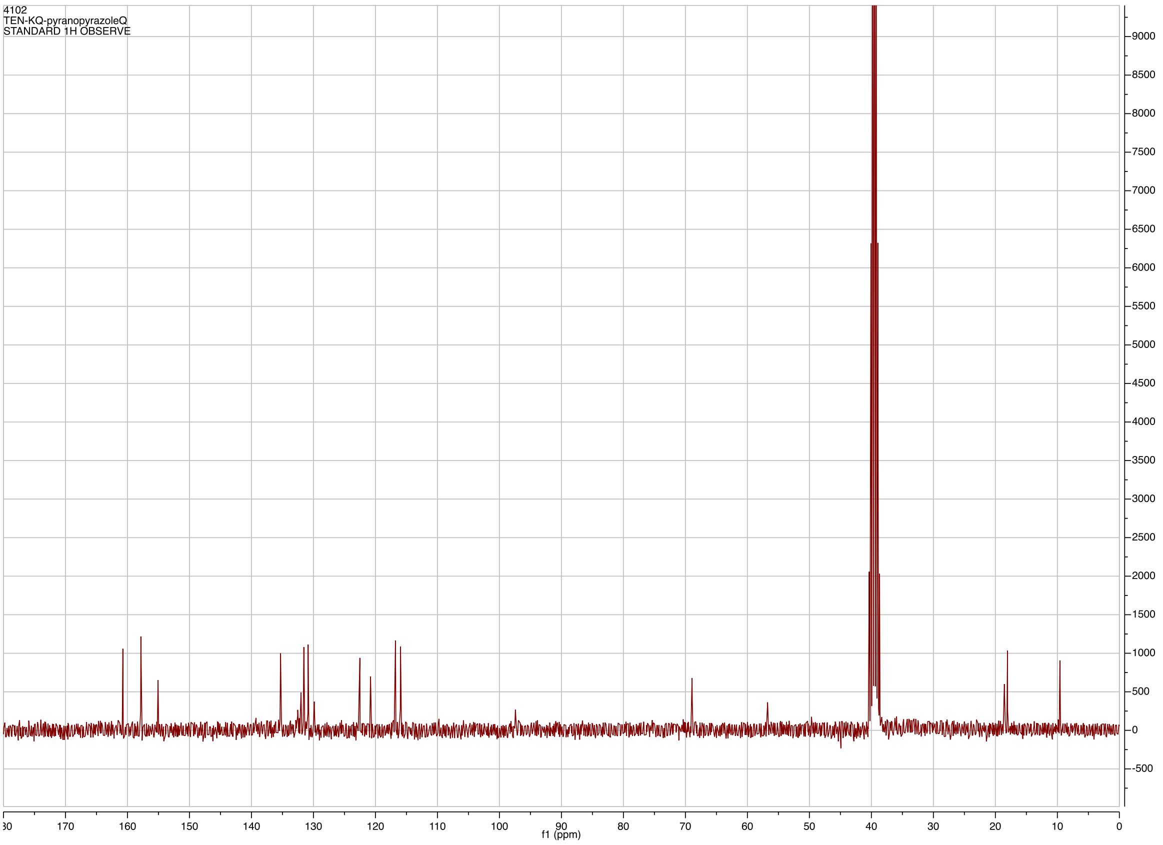
4102
TEN-KQ-pyranopyrazoleQ
STANDARD 1H OBSERVE
f1 (ppm)

6-amino-4-(2,4-dimethyl-5-(phenoxymethyl)phenyl)-3-methyl-1,4-dihydropyrano[2,3-*c*]pyrazole-5-carbonitrile (10r)

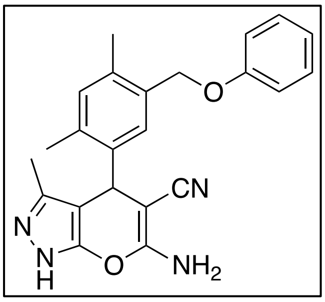

UPLC: Rt = 1.82, 1.88 (2,4-dihydro tautomer);
UPLC-MS (ESI) calculated for $C_{23}H_{23}N_4O_2$ $[M + H]^-$: *m/z* = 387.18, found *m/z* = 387.2;
^{1}H NMR (300 MHz, DMSO-d_6): δ = 12.06 (s, 1H, ArN**H**), 7.38-7.19 (m, 3H, Ar**H**), 7.14 – 6.87 (m, 4H, Ar**H**), 6.83 (s, 2H, ArN**H$_2$**), 4.97 (s, 2H, ArC**H$_2$**OAr), 4.81 (s, 1H, C**H**Ar), 2.25 (s, 6H 2 × $ArCH_3$), 1.64 (s, 3H $ArCH_3$);
^{13}C NMR (75 MHz, DMSO-d_6): δ = 160.74, 158.38, 155.07, 139.21, 135.34, 135.03, 134.83, 132.75, 132.44, 129.72, 129.41, 120.81, 120.67, 114.86, 97.38, 68.04, 56.80, 18.55, 18.04 (two overlapping), 9.62.

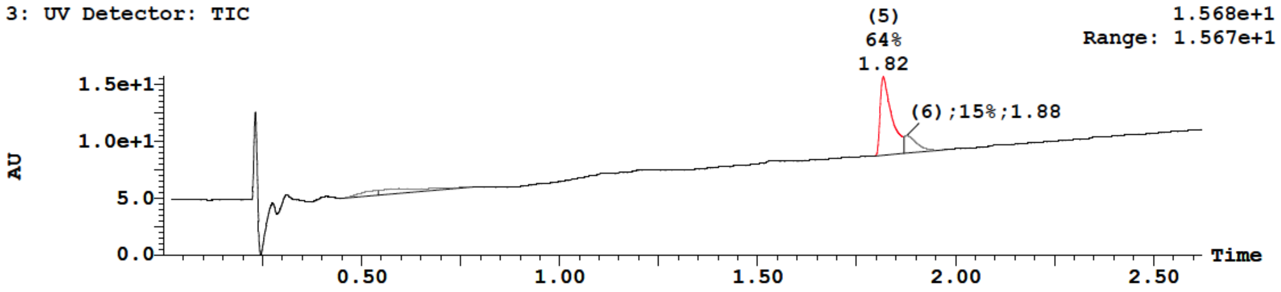

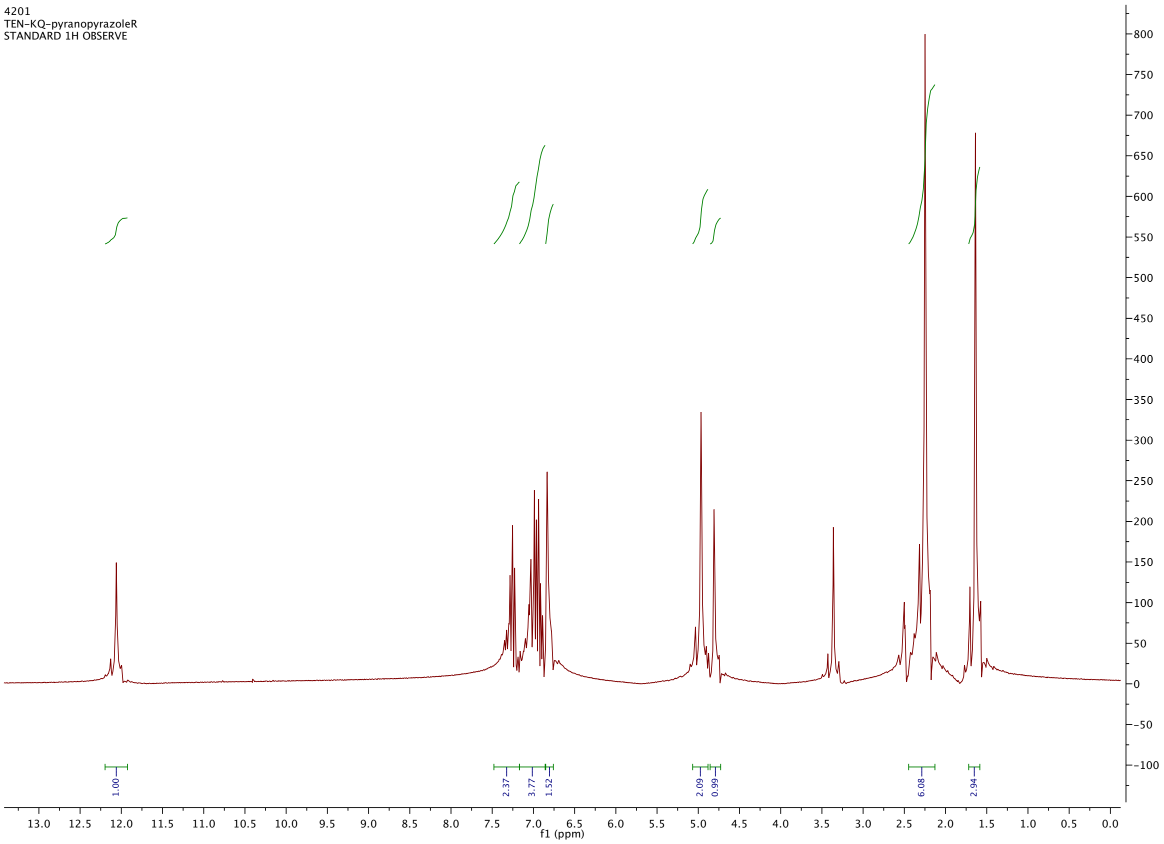

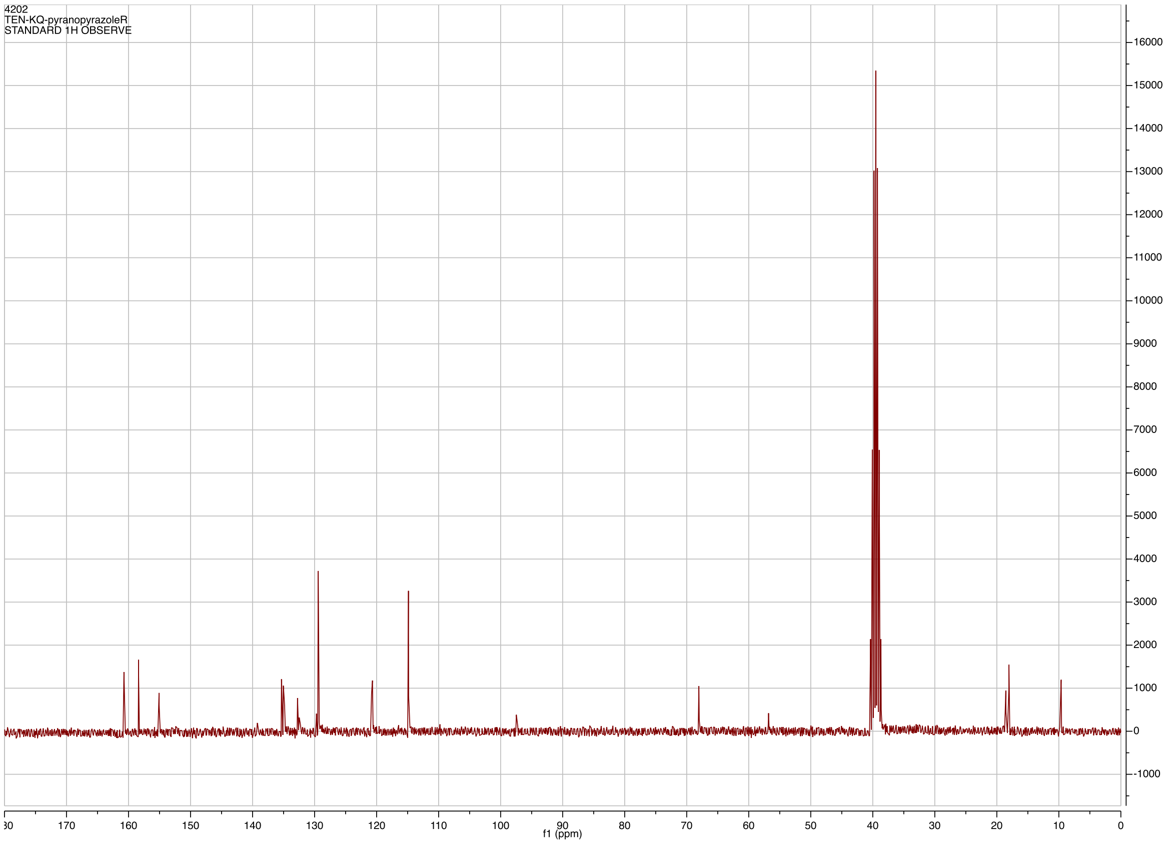
4202
TEN-KQ-pyranopyrazoleR
STANDARD 1H OBSERVE
f1 (ppm)

6-amino-4-(5-(((5-chloroquinolin-8-yl)oxy)methyl)-2,4-dimethylphenyl)-3-methyl-1,4-dihydropyrano[2,3-*c*]pyrazole-5-carbonitrile (10s)

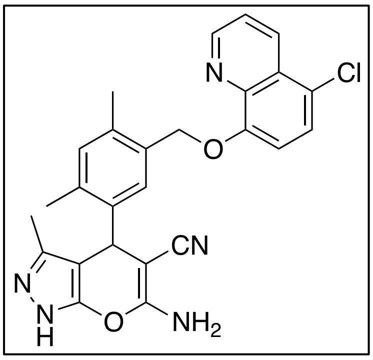

UPLC: Rt = 1.72, 1.78 (2,4-dihydro tautomer);
UPLC-MS (ESI) calculated for $C_{26}H_{23}ClN_5O_2$ [M + H]$^-$: *m/z* = 472.2 (^{35}Cl), 474.2 (^{37}Cl), found *m/z* = 472.2 (100 %), 474.2 (33 %);
^{1}H NMR (300 MHz, DMSO-*d*$_6$): δ = 12.04 (s, 1H, ArN**H**), 9.05-8.82 (m, 1H, Ar**H**), 8.57-8.38 (m, 1H, Ar**H**), 7.80 – 7.53 (m, 2H, Ar**H**), 7.28 (m, 1H, Ar**H**), 7.14 (s, 1H, Ar**H**), 7.01 (s, 1H, Ar**H**), 6.83 (s, 2H, ArN**H**$_2$), 5.19 (s, 2H, ArC**H**$_2$OAr), 4.86 (s, 1H, C**H**Ar), 2.31 (s, 3H ArCH$_3$), 2.29 (s, 3H ArCH$_3$), 1.70 (s, 3H ArCH$_3$);
^{13}C NMR (75 MHz, DMSO-*d*$_6$): δ = 160.75, 155.06, 153.64, 149.86, 140.49, 135.50, 135.03, 132.50, 132.47, 132.17, 130.13, 130.07, 126.69, 126.16, 123.00, 120.83, 120.77, 110.34, 97.50, 69.23, 56.80, 40.08, 18.60, 18.13, 9.66.

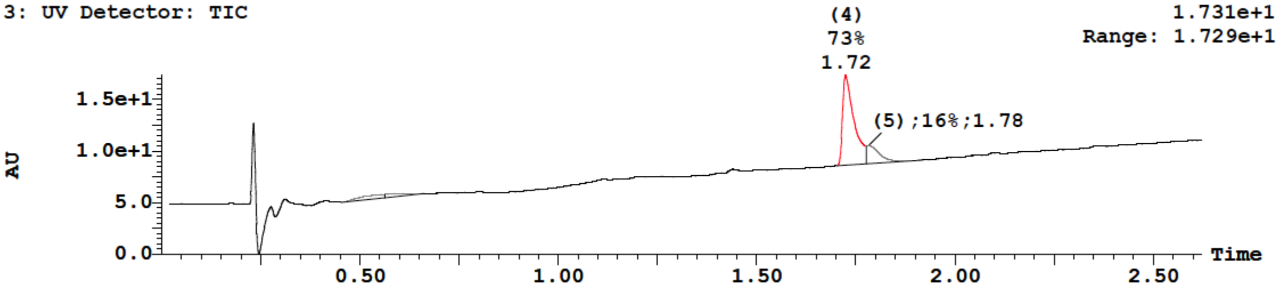

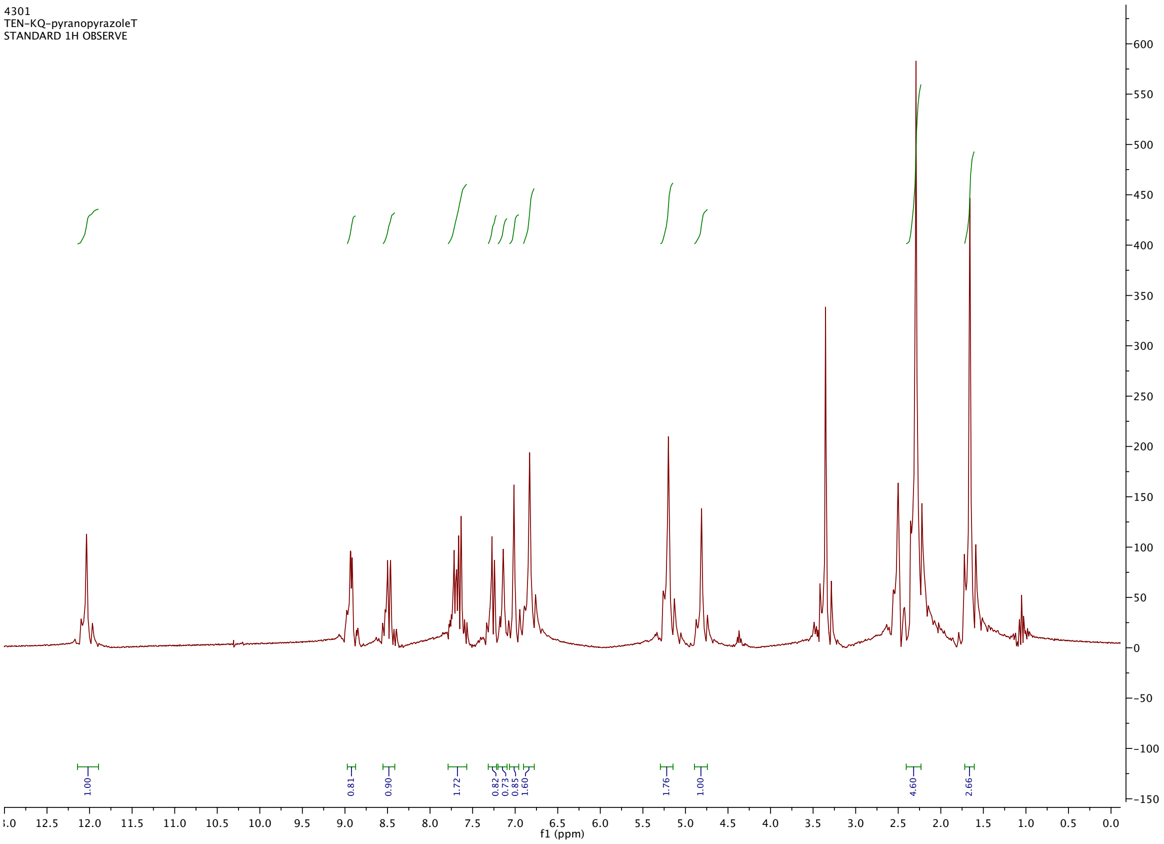

4301
TEN-KQ-pyranopyrazoleT
STANDARD 1H OBSERVE
f1 (ppm)

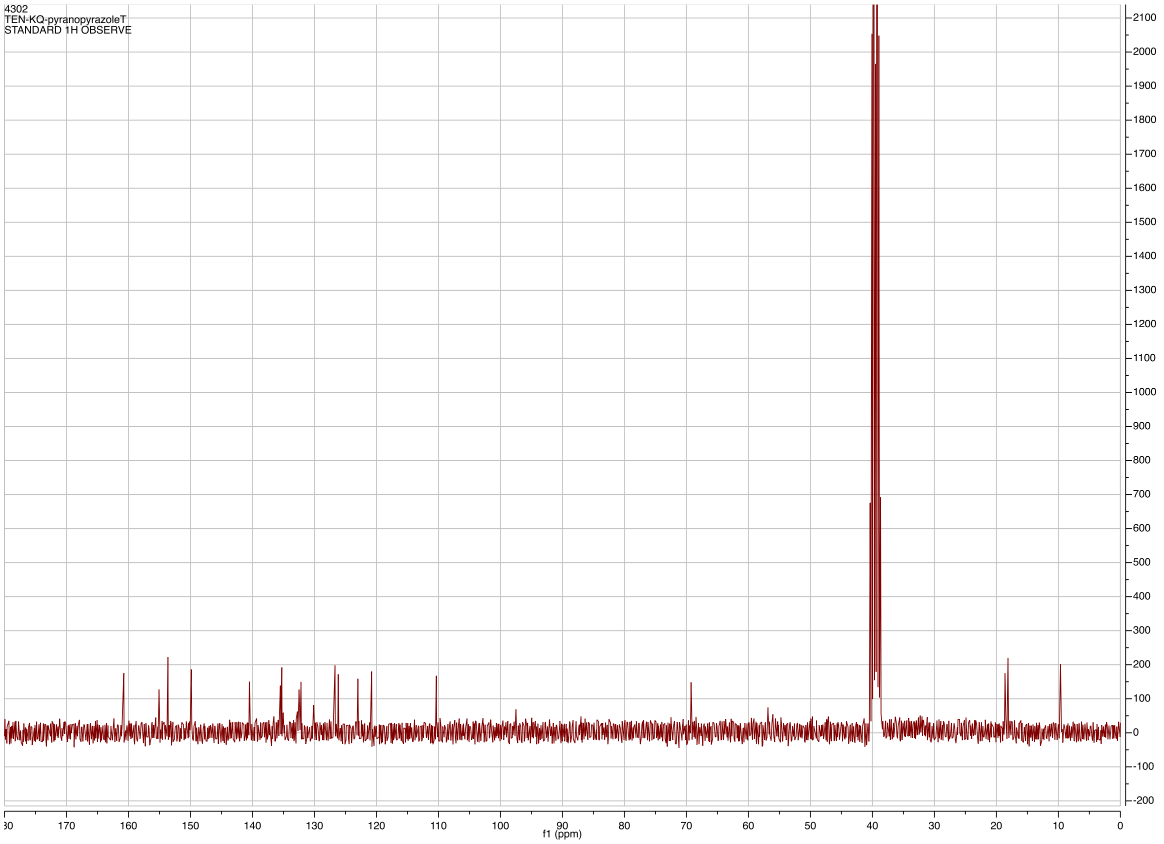

4302
TEN-KQ-pyranopyrazoleT
STANDARD 1H OBSERVE
f1 (ppm)

6-amino-4-(5-(((4-chloronaphthalen-1-yl)oxy)methyl)-2,4-dimethylphenyl)-3-methyl-1,4-dihydropyrano[2,3-*c*]pyrazole-5-carbonitrile (10t)

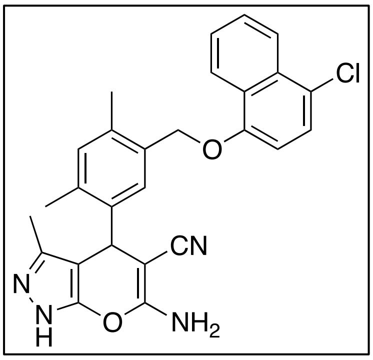

UPLC: Rt = 2.21, 2.28 (2,4-dihydro tautomer);
UPLC-MS (ESI) calculated for $C_{27}H_{24}ClN_4O_2$ $[M + H]^-$: *m/z* = 471.2 (^{35}Cl), 473.2 (^{37}Cl), found *m/z* = 471.2 (100 %), 473.2 (33 %);
^{1}H NMR (300 MHz, DMSO-d_6): δ = 12.10 (s, 1H, ArN**H**), 8.25-8.01 (m, 2H, Ar**H**), 7.74 – 7.56 (m, 2H, Ar**H**), 7.58 (d, *J* = 8.3 Hz, 2H, Ar**H**), 7.25 (s, 1H, Ar**H**), 7.07 (d, *J* = 8.4 Hz, 1H, Ar**H**), 7.02 (s, 1H, Ar**H**), 6.88 (s, 2H, ArN$\mathbf{H_2}$), 5.19 (s, 2H, ArC$\mathbf{H_2}$OAr), 4.86 (s, 1H, **CH**Ar), 2.31 (s, 3H $ArCH_3$), 2.29 (s, 3H $ArCH_3$), 1.70 (s, 3H $ArCH_3$);
^{13}C NMR (75 MHz, DMSO-d_6): δ = 160.81, 155.21, 153.04, 135.31, 134.61, 134.57, 134.38, 132.65, 132.37, 130.44, 127.98, 126.62, 126.25, 126.13, 123.67, 122.08, 121.76, 120.91, 109.35, 106.29, 97.69, 68.25, 56.97, 18.55, 18.02 (two overlapping), 9.66.

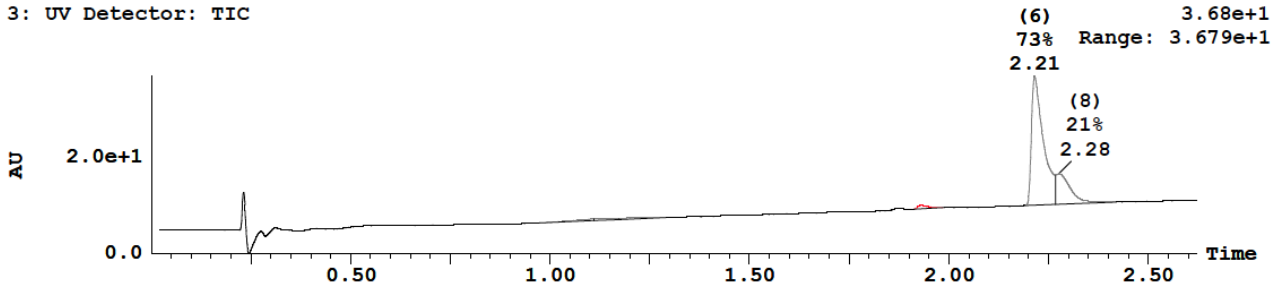

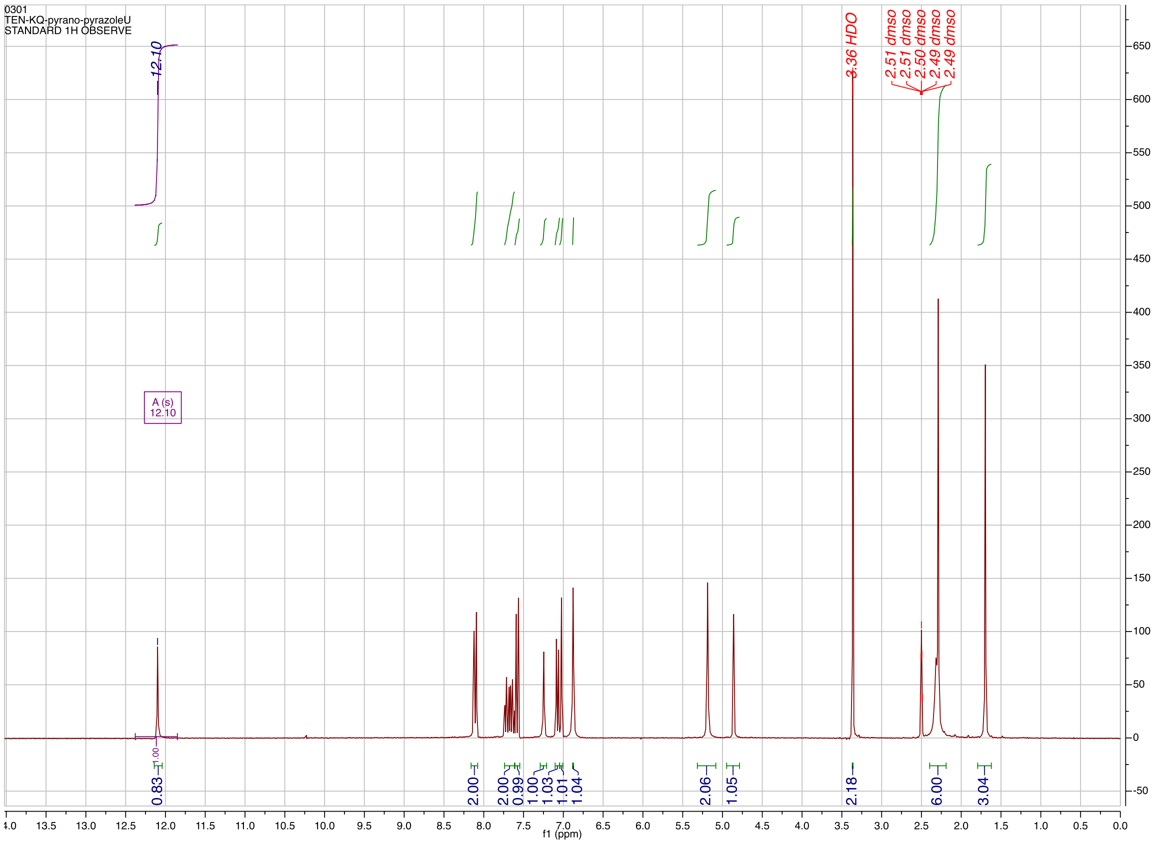

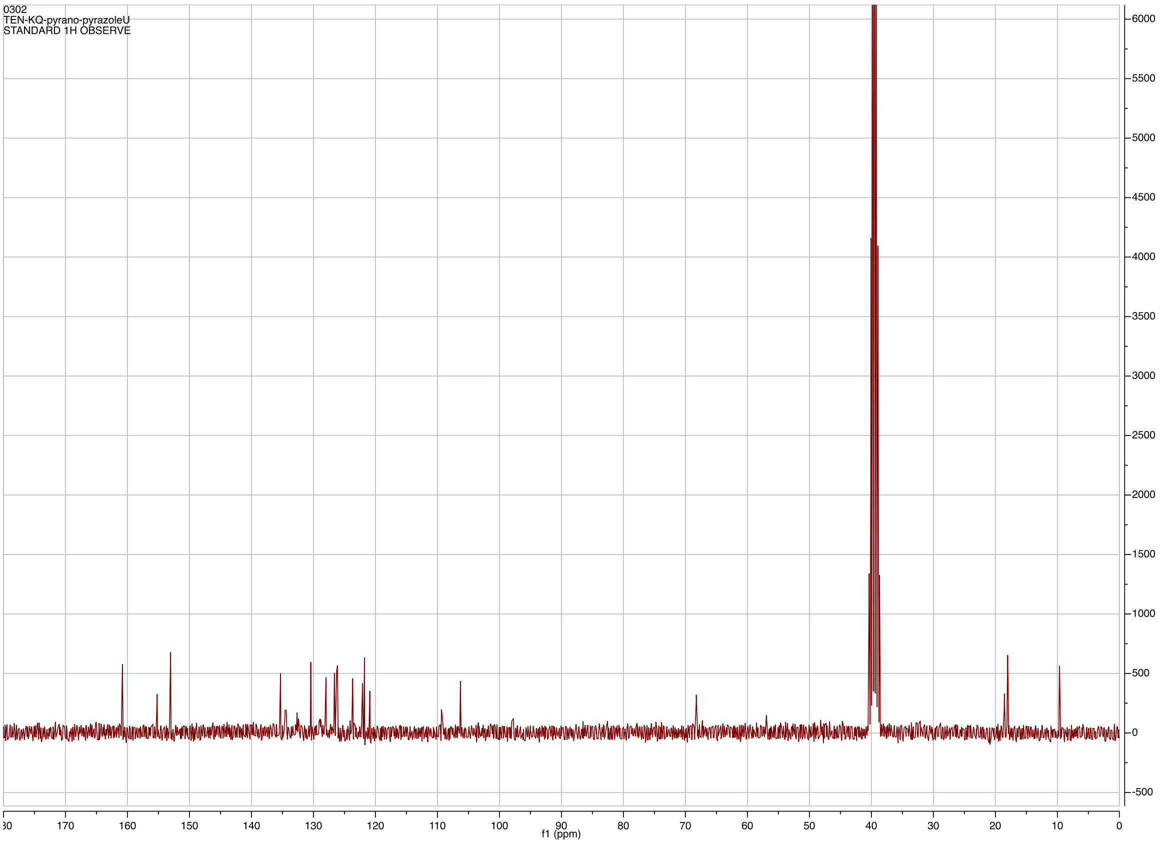
0302
TEN-KQ-pyrano-pyrazoleU
STANDARD 1H OBSERVE
f1 (ppm)

6-amino-4-(4-chlorophenyl)-3-methyl-1,4-dihydropyrano[2,3-*c*]pyrazole-5-carbonitrile (10u)

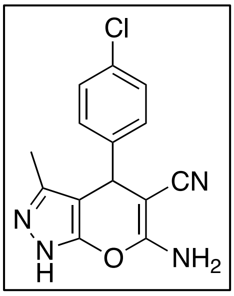

UPLC: Rt = 1.42 (2,4-dihydro tautomer seen as a shoulder);
UPLC-MS (ESI) calculated for $C_{14}H_{12}ClN_4O$ $[M + H]^-$: *m/z* = 287.1 (^{35}Cl), 289.1 (^{37}Cl), found *m/z* = 287.1 (100 %), 289.1 (33 %);
^1H NMR (400 MHz, DMSO-d_6): δ = 12.13 (s, 1H, ArN**H**), 7.38 (d, *J* = 8.4 Hz, 2H, 2 × Ar**H**), 7.19 (d, *J* = 8.5 Hz, 2H, 2 × Ar**H**), 6.92 (s, 2H, ArN**H**$_2$), 4.63 (s, 1H, C**H**Ar), 1.79 (s, 3H, ArC**H**$_3$);
^{13}C NMR (100 MHz, DMSO-d_6): δ = 160.90, 154.69, 143.49, 135.65, 131.21, 129.36, 128.45, 120.64, 97.19, 56.74, 35.55, 9.74.

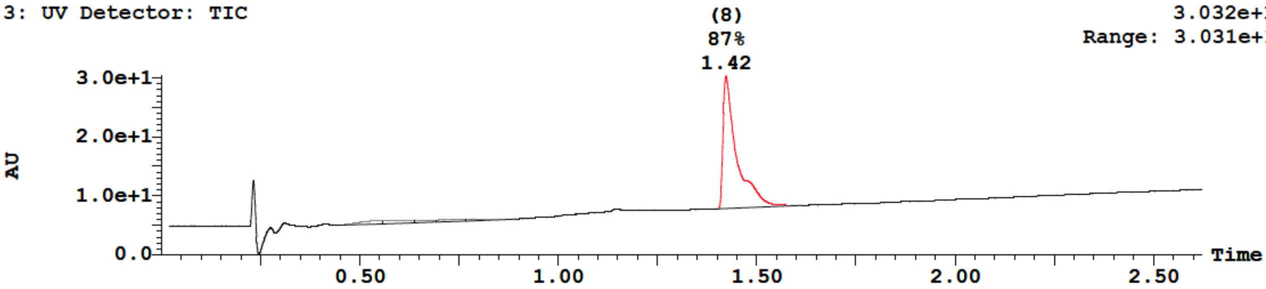

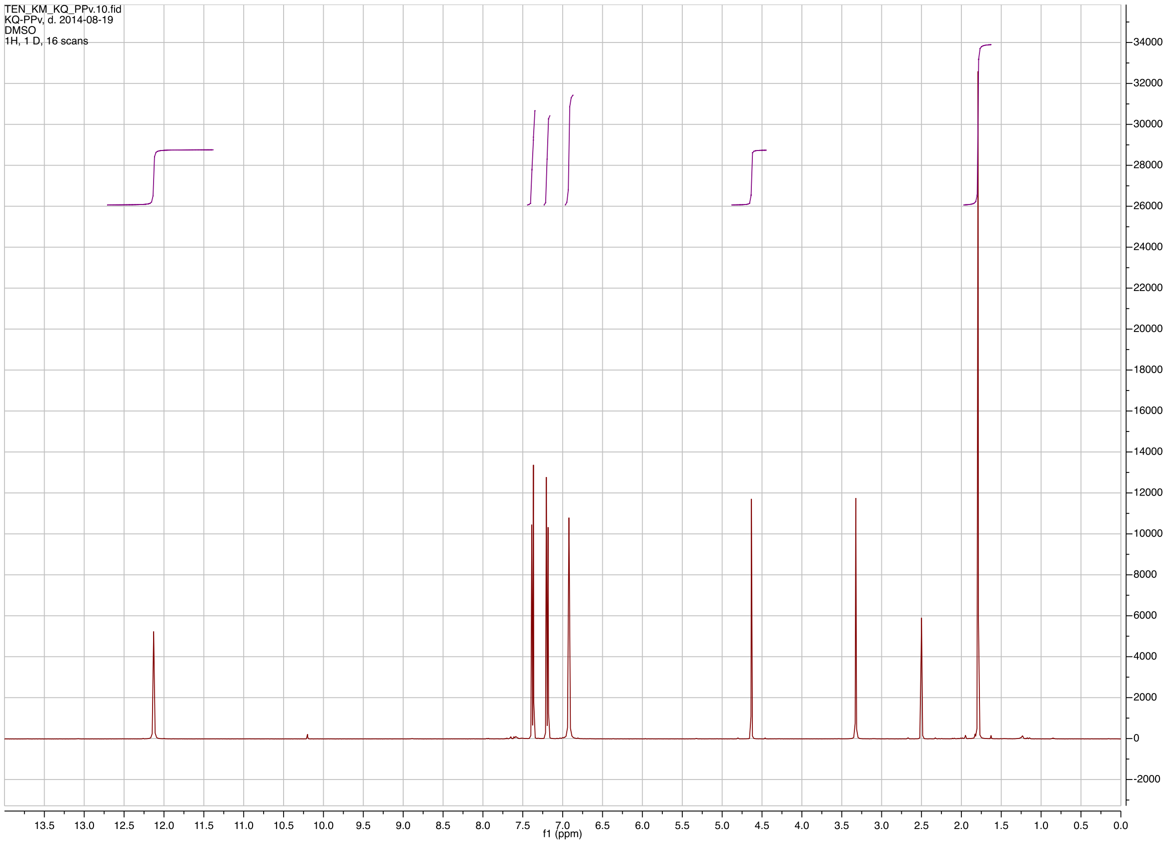

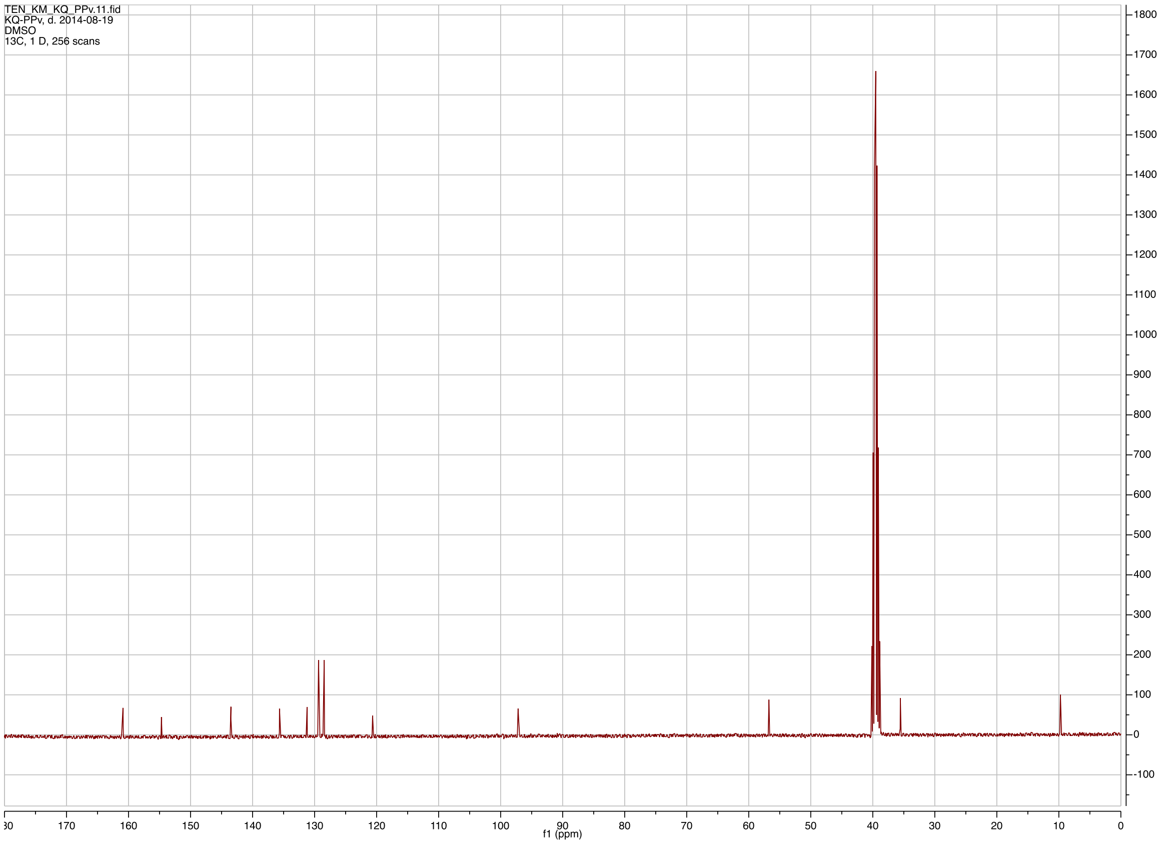
TEN_KM_KQ_PPv.11.fid
KQ-PPv, d. 2014-08-19
DMSO
13C, 1 D, 256 scans
f1 (ppm)

6-amino-3-methyl-4-(*o*-tolyl)-1,4-dihydropyrano[2,3-*c*]pyrazole-5-carbonitrile (10v)

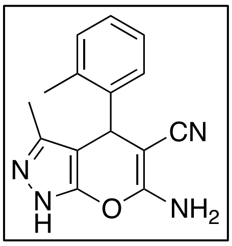

UPLC: Rt = 1.34 (2,4-dihydro tautomer seen as a shoulder);
UPLC-MS (ESI) calculated for $C_{15}H_{15}N_4O$ $[M + H]^{-}$: *m/z* = 267.1, found *m/z* = 267.1;
^{1}H NMR (400 MHz, DMSO-d_6): δ = 12.07 (s, 1H, ArN**H**), 7.18-7.06 (m, 3H, 3 × Ar**H**), (dd, *J* = 7.7, 2.1 Hz, 1H, Ar**H**), 6.82 (s, 2H, ArN**H**$_2$), 4.84 (s, 1H, C**H**Ar), 2.28 (s, 3H, ArC**H**$_3$), 1.68 (s, 3H, ArC**H**$_3$);
^{13}C NMR (100 MHz, DMSO-d_6): δ = 160.74, 155.06, 141.86, 135.30, 134.95, 130.46, 128.88, 126.56, 126.30, 120.69, 97.53, 56.73, 33.07, 18.90, 9.52.

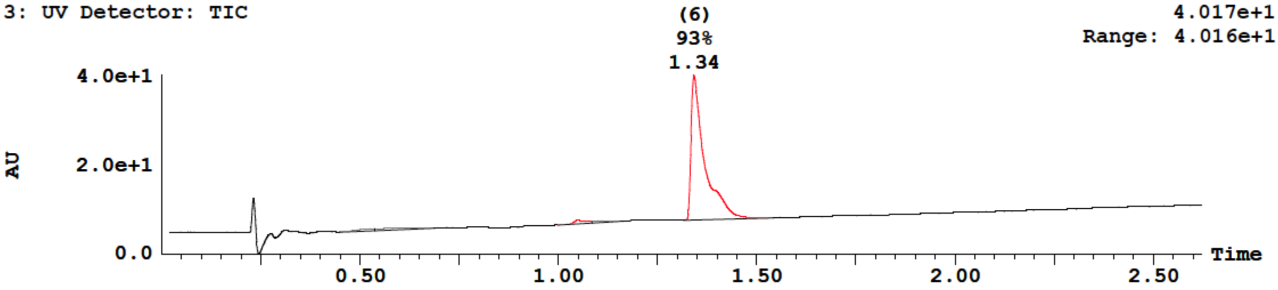

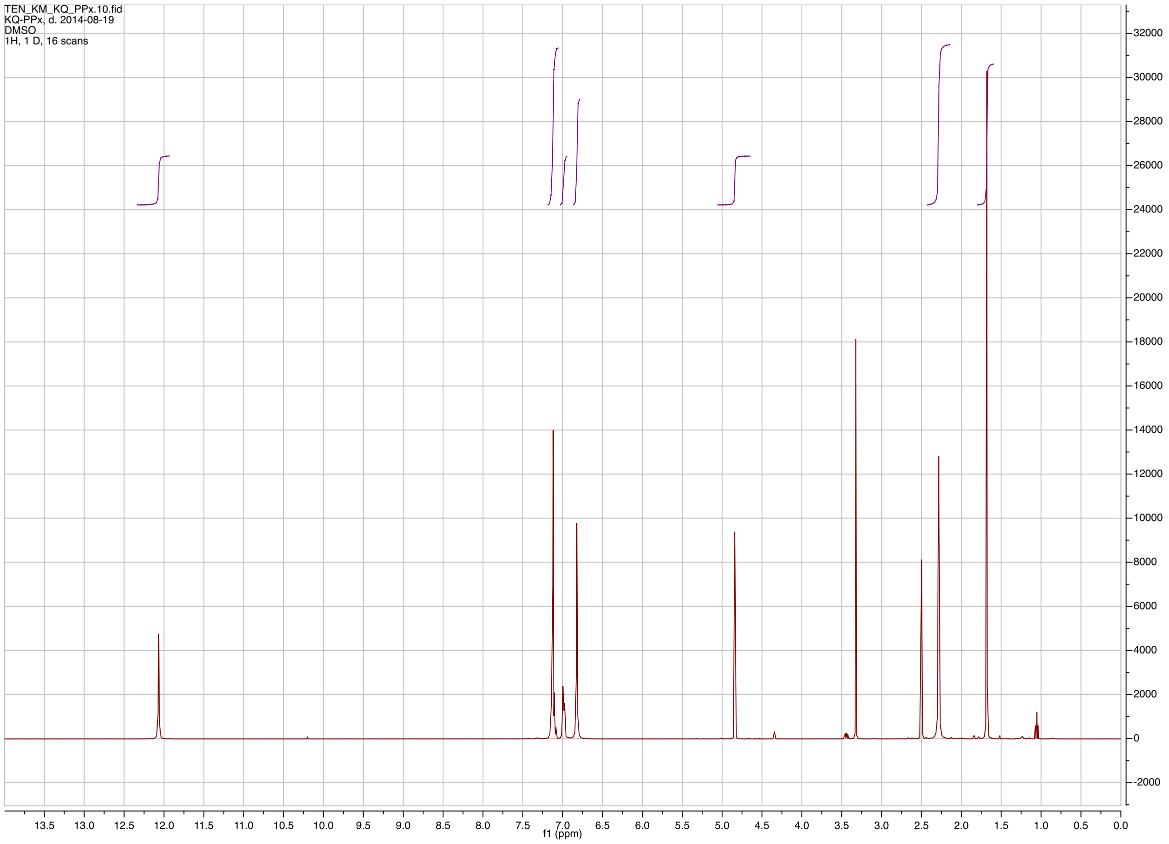

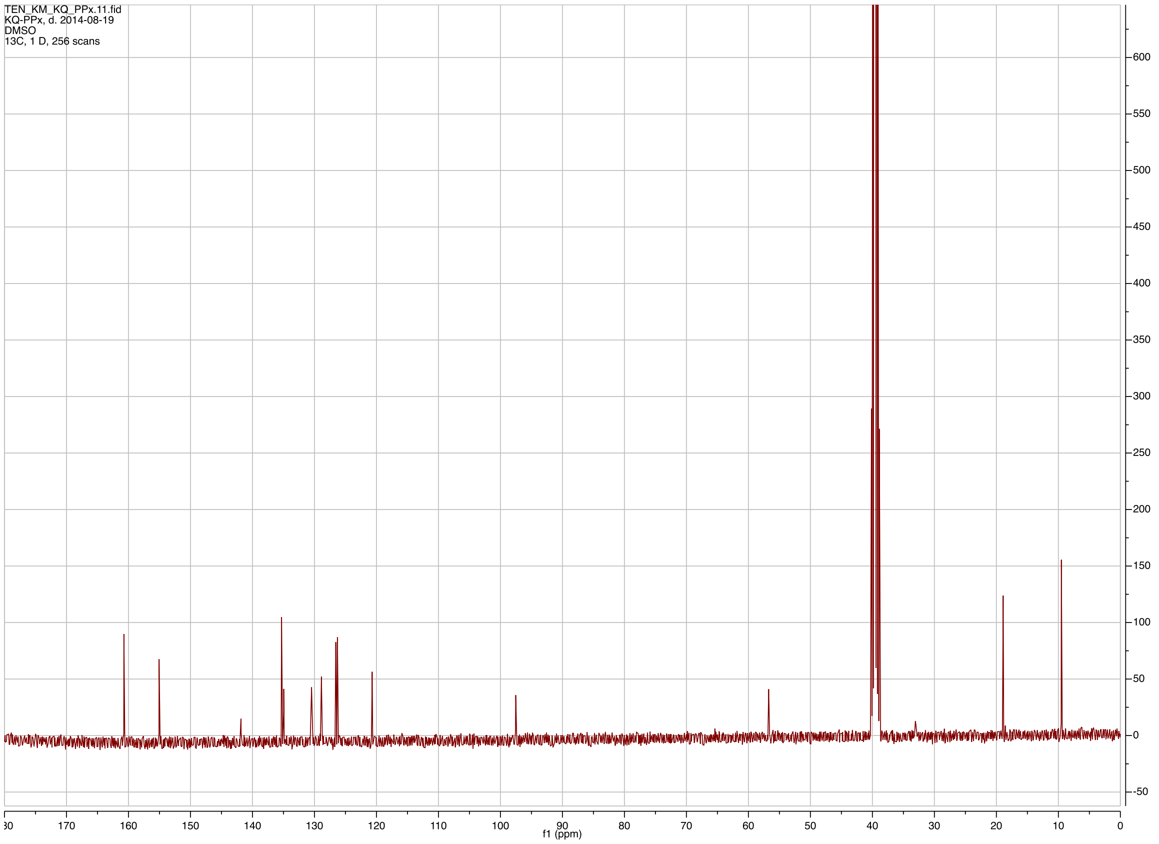

TEN_KM_KQ_PPx.11.fid
KQ-PPx, d. 2014-08-19
DMSO
13C, 1 D, 256 scans
f1 (ppm)

6-amino-4-(2,4-dichlorophenyl)-3-methyl-1,4-dihydropyrano[2,3-*c*]pyrazole-5-carbonitrile (10x)

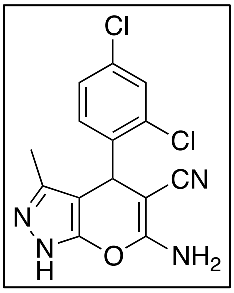

UPLC-UV: Rt = 1.54 (2,4-dihydro tautomer seen as a shoulder);
UPLC-MS (ESI) calculated for $C_{14}H_{11}Cl_2N_4O_2$ $[M + H]^-$: *m/z* = 321.0 (2 x ^{35}Cl), 323.0 (1 x ^{37}Cl, 1 x ^{35}Cl,), found *m/z* = 321.0 (100 %), 323.0 (64 %);
^1H NMR (400 MHz, DMSO-d_6): δ = 12.17 (s, 1H, ArN**H**), 7.58 (s, 1H, Ar**H**), 7.42 (d, *J* = 7.6 Hz, 1H, Ar**H**), 7.42 (d, *J* = 7.7 Hz, 1H, Ar**H**), 7.02 (s, 2H, ArN$\mathbf{H}_2$), 5.06 (s, 1H, C**H**Ar), 1.78 (s, 3H, ArC$\mathbf{H}_3$);
^{13}C NMR (100 MHz, DMSO-d_6): δ = 161.36, 154.93, 140.13, 135.49, 132.87, 132.16 (two overlapping), 128.88, 128.08, 120.33, 96.38, 55.24, 33.11, 9.60;
IR (neat): 1410, 1585, 2183, 3101, 3246.

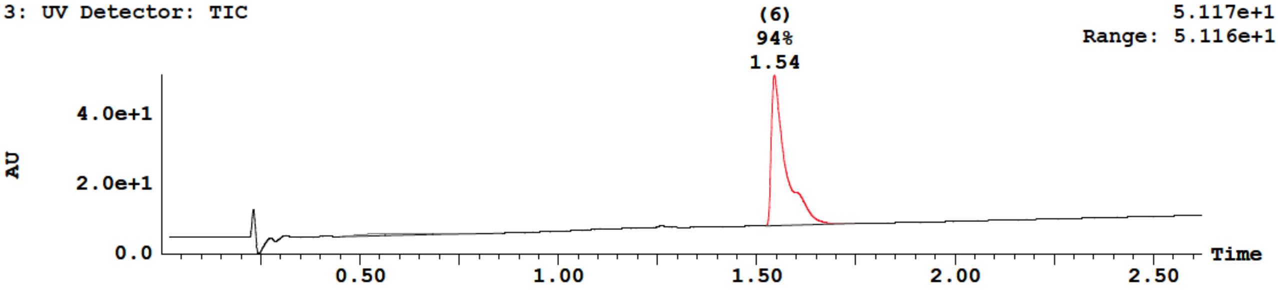

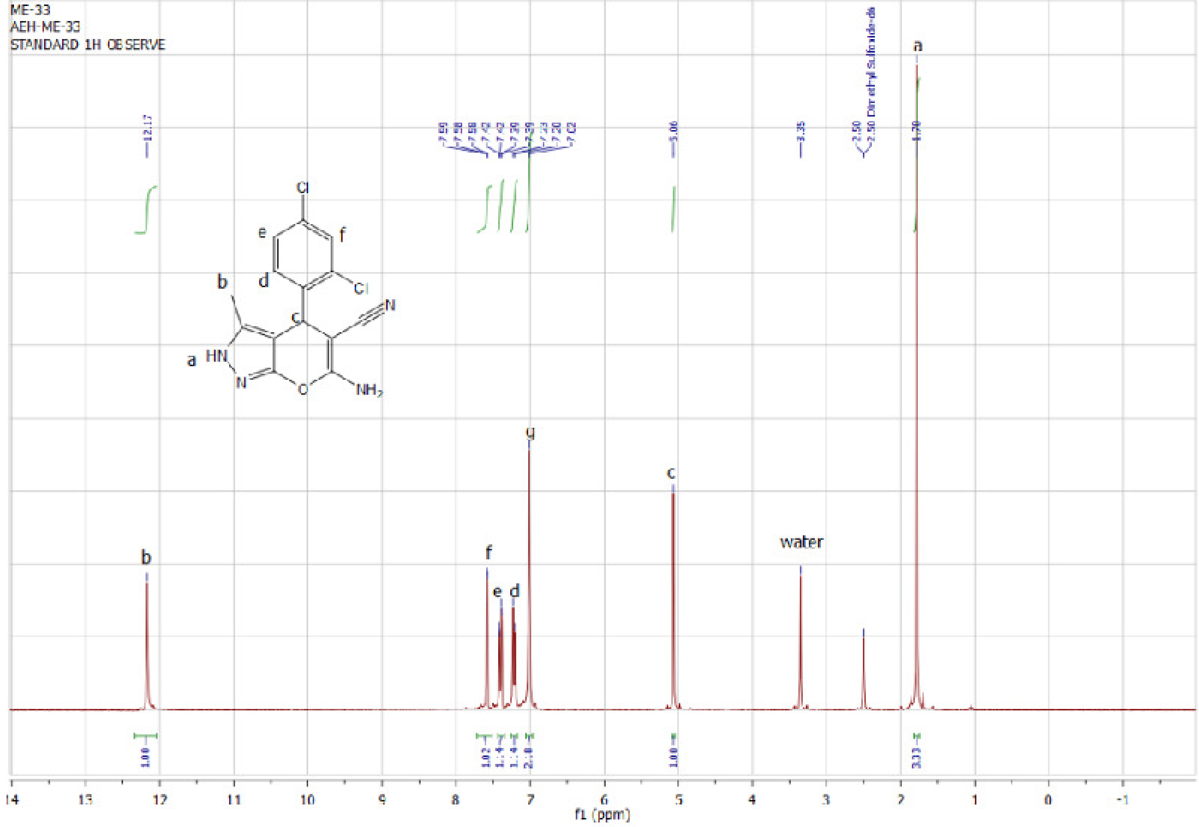

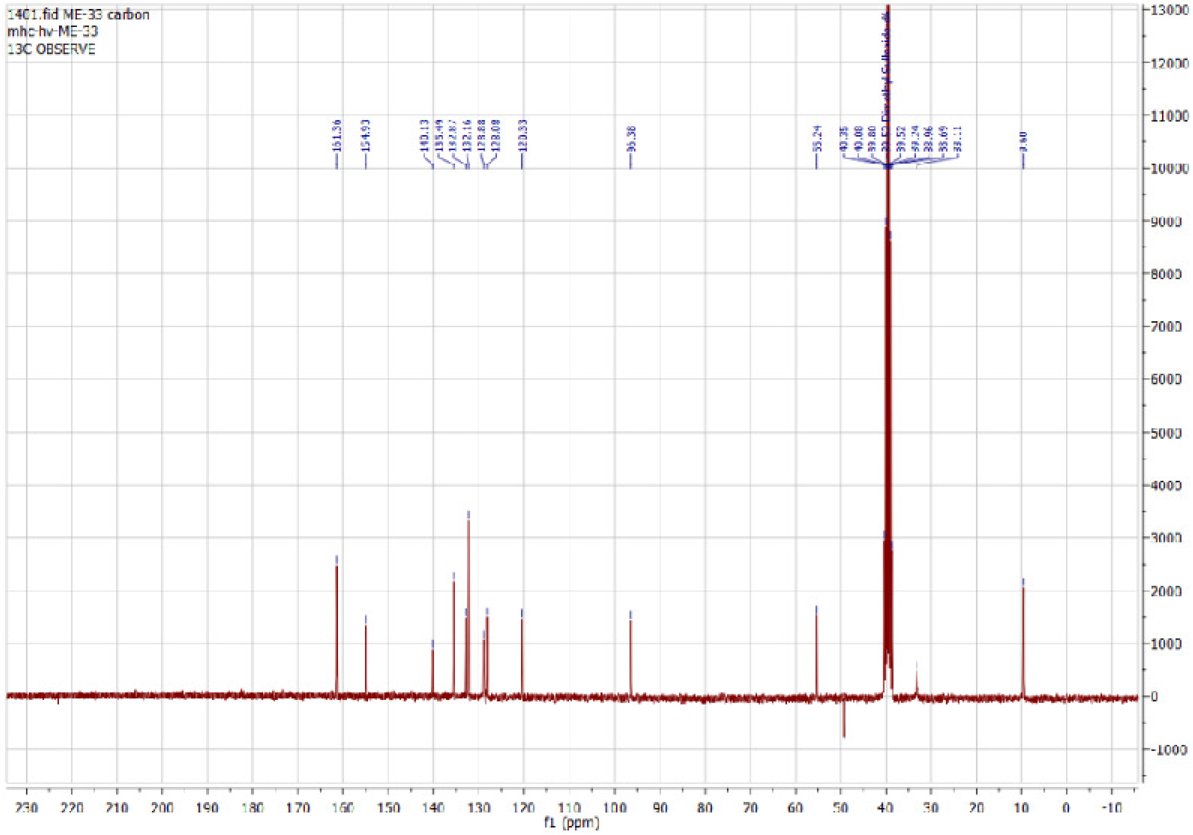

1401.fid ME-33 carbon
mhc-hv-ME-33
13C OBSERVE
161.36
154.93
140.13
135.49
132.16
128.88
128.08
120.33
96.38
55.24
40.35
40.08
39.80
39.52
38.96
38.69
9.60
f1 (ppm)

6-amino-4-(4-methoxy-2,3-dimethylphenyl)-3-methyl-1,4-dihydropyrano[2,3-*c*]pyrazole-5-carbonitrile (10y)

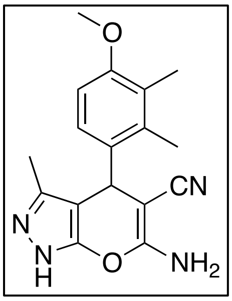

UPLC: Rt = 1.47 (2,4-dihydro tautomer seen as a shoulder).
UPLC-MS (ESI) calculated for $C_{17}H_{19}N_4O_2$ $[M + H]^-$: *m/z* = 311.2, found *m/z* = 311.1
^{1}H NMR (300 MHz, DMSO-d_6): δ = 12.04 (s, 1H, ArN**H**), 6.80-6.75 (m, 4H, 2 × Ar**H** + 2 × ArN**H**$_2$), 4.85 (s, 1H, C**H**Ar), 3.22 (s, 3H, ArOC**H**$_3$), 2.10 (s, 3H, ArC**H**$_3$), 2.09 (s, 3H, ArC**H**$_3$), 1.69 (s, 3H, ArC**H**$_3$);

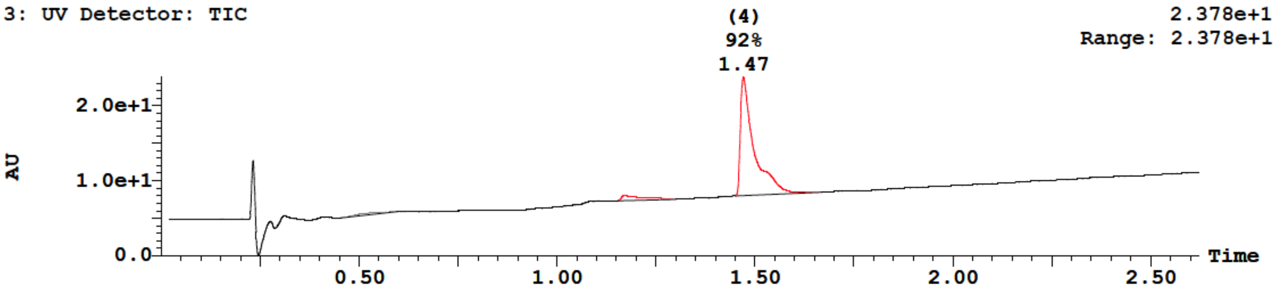

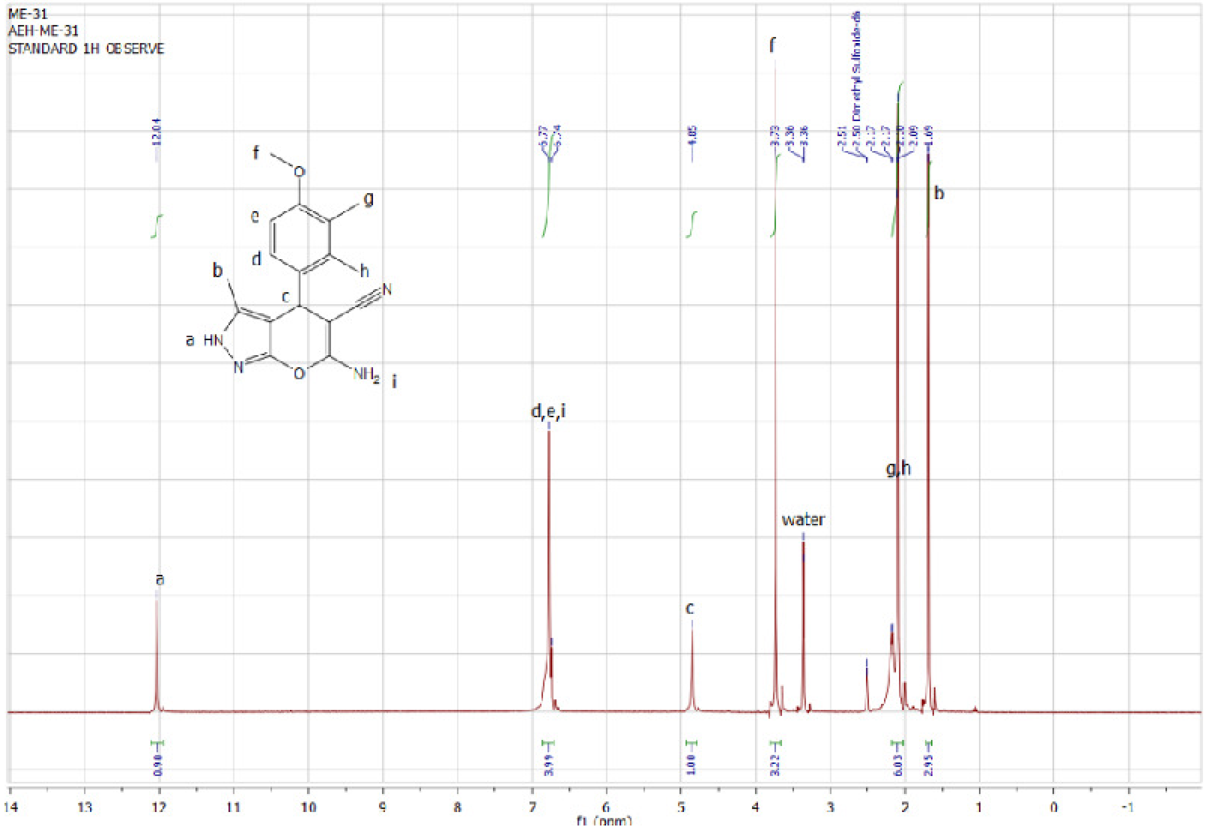

6-amino-3-methyl-4-phenyl-1,4-dihydropyrano[2,3-*c*]pyrazole-5-carbonitrile (10z)

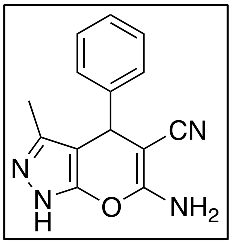

UPLC-UV: Rt = 1.25 (2,4-dihydro tautomer seen as a shoulder).
UPLC-MS (ESI) calculated for $C_{14}H_{13}N_4O$ $[M + H]^-$: m/z = 253.1, found m/z = 253.1;
^{1}H-NMR (400 MHz, DMSO-d_6): δ = 12.09 (s, 1H, ArN**H**), 7.31 (dd, J = 8.0, 6.8 Hz, 2H, Ar**H**), 7.23 (d, J = 7.4 Hz, 1H, Ar**H**), 7.20 – 7.13 (m, 2H, Ar**H**), 6.86 (s, 2H, ArN**H**$_2$), 4.59 (s, 1H, C**H**Ar), 1.78 (s, 3H ArCH$_3$);
^{13}C-NMR (100 MHz, DMSO-d_6): δ = 160.86, 154.76, 144.45, 135.55, 128.43, 127.46, 126.72, 120.79, 97.64, 57.17, 36.23, 9.74.

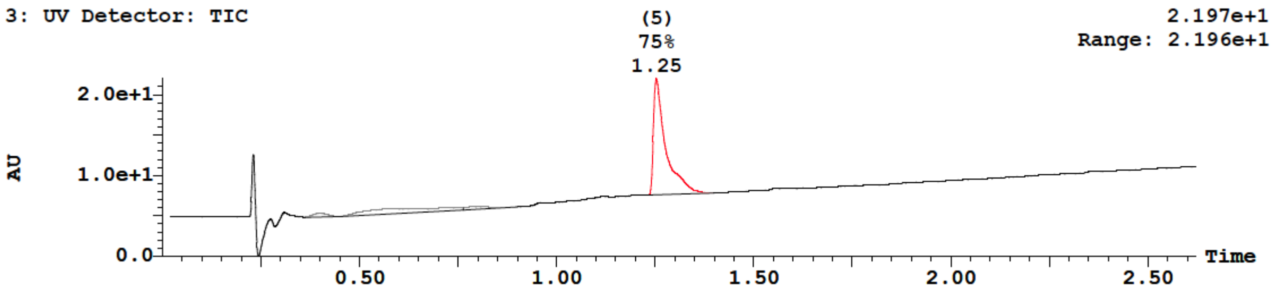

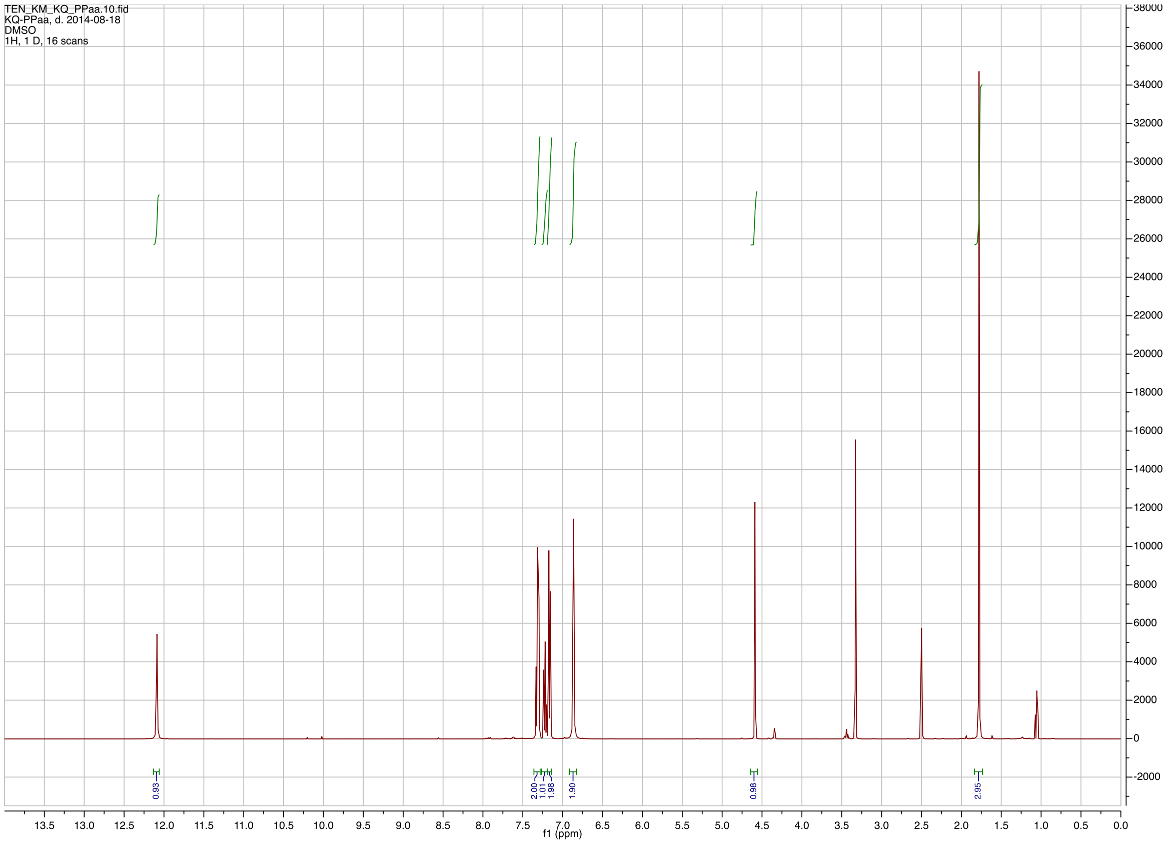

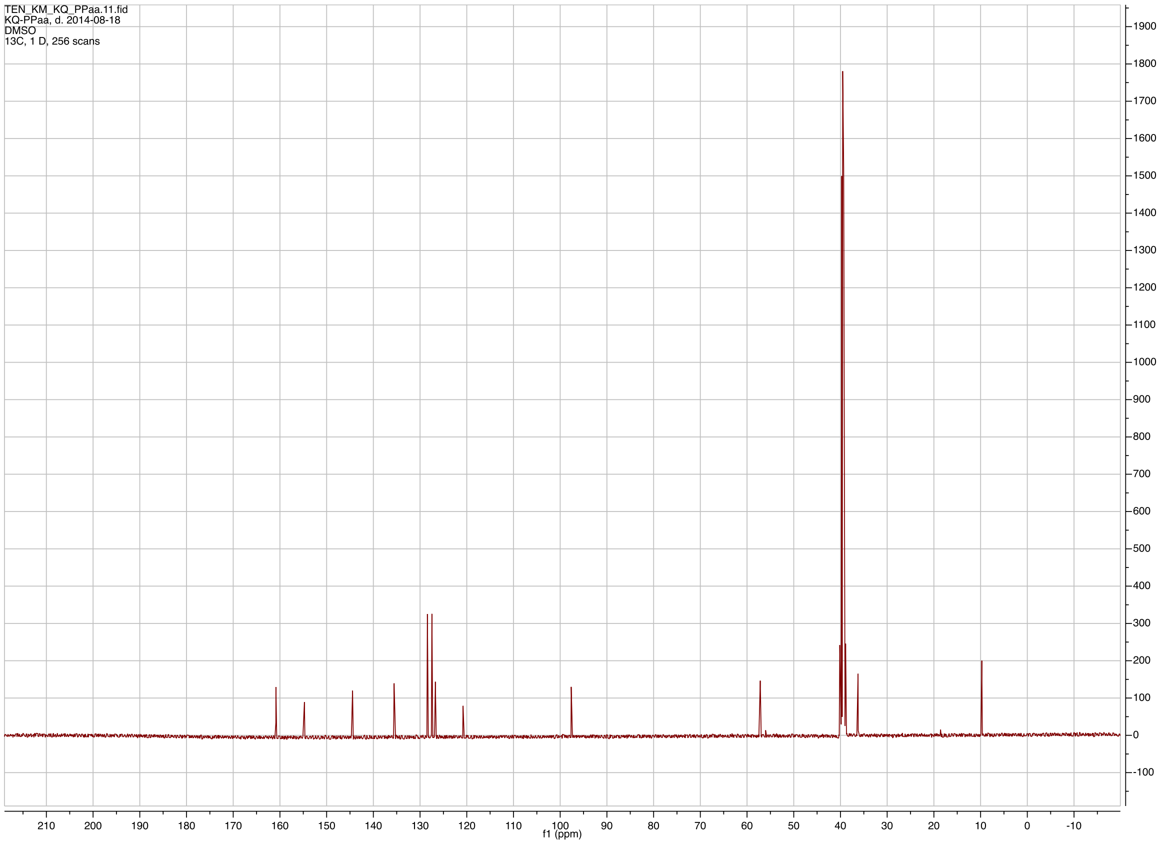
TEN_KM_KQ_PPaa.11.fid
KQ-PPaa, d. 2014-08-18
DMSO
13C, 1 D, 256 scans
f1 (ppm)

6-amino-4-(2,4-dimethylphenyl)-3-methyl-1,4-dihydropyrano[2,3-*c*]pyrazole-5-carbonitrile (10aa)

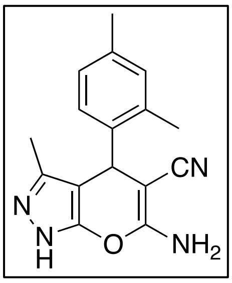

UPLC-UV: Rt = 1.48 (2,4-dihydro tautomer seen as a shoulder);
UPLC-MS (ESI) calculated for $C_{16}H_{17}N_4O$ $[M + H]^-$: *m/z* = 281.1, found *m/z* = 281.1;
^{1}H-NMR (400 MHz, DMSO-d_6): δ = 12.05 (s, 1H, ArN**H**), 6.93 (d, *J* = 2.3 Hz, 2H, Ar**H**), 6.86 (d, *J* = 8.3 Hz, 1H, Ar**H**), 6.79 (s, 2H, ArN**H**$_2$), 4.79 (s, 1H, C**H**Ar), 2.24 (s, 3H ArC**H**$_3$), 2.23 (s, 3H ArC**H**$_3$), 1.69 (s, 3H ArC**H**$_3$);
^{13}C NMR (100 MHz, DMSO-d_6): δ = 160.65, 155.05, 135.40, 135.27 (two overlapping signals), 134.72, 131.11, 128.85, 126.93 (two overlapping signals), 120.71, 97.62, 56.94, 20.53 (two overlapping signals), 18.83, 9.56.

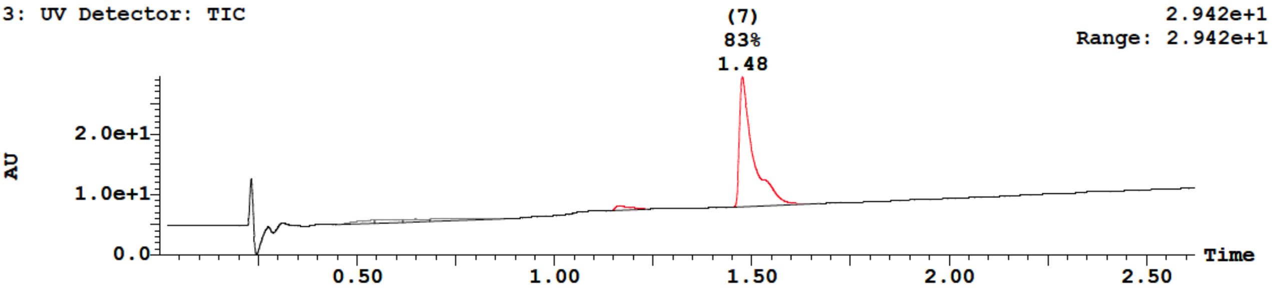

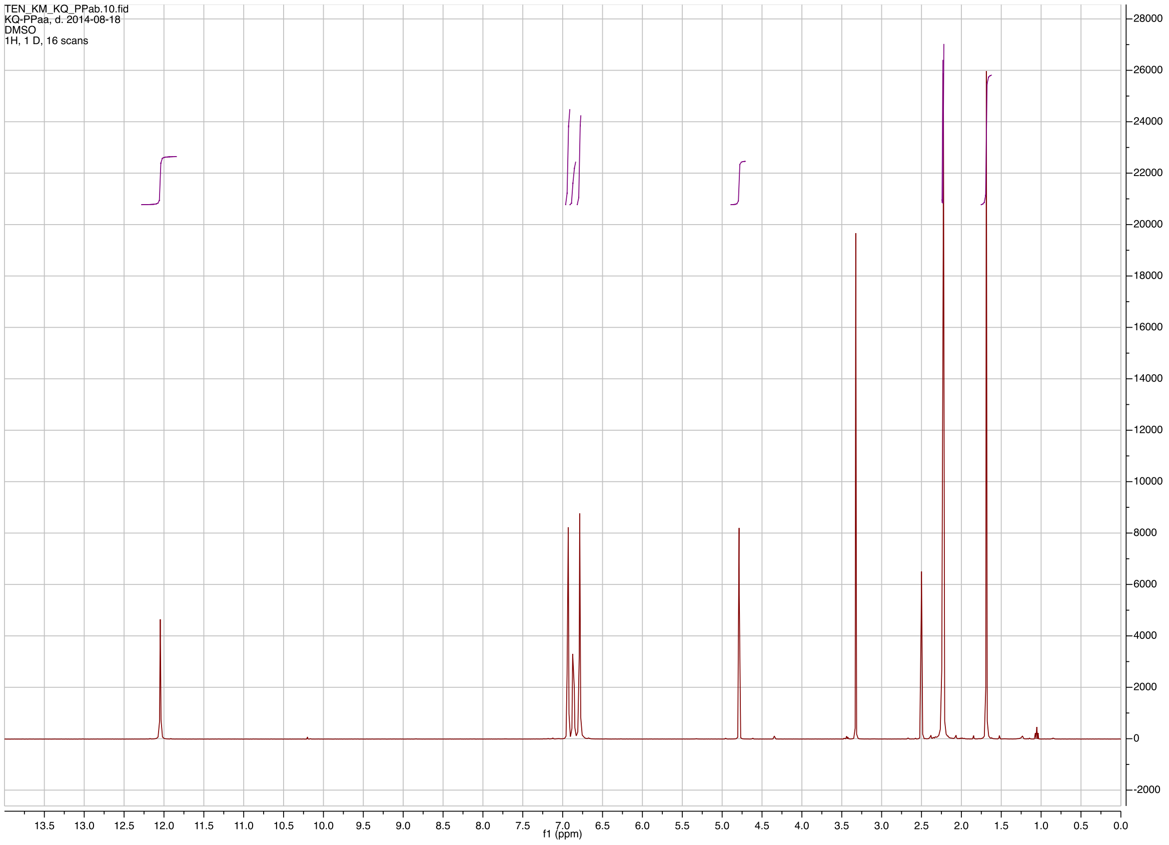

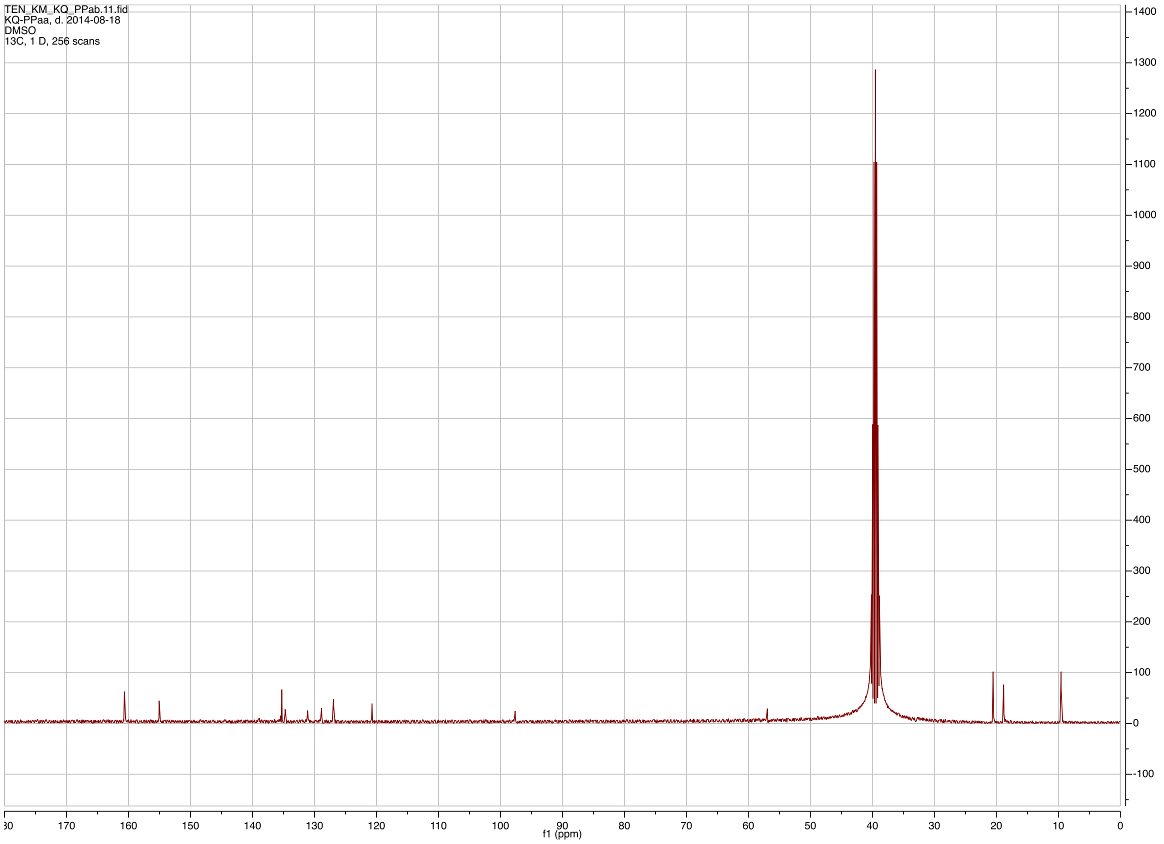
TEN_KM_KQ_PPab.11.fid
KQ-PPaa, d. 2014-08-18
DMSO
13C, 1 D, 256 scans
f1 (ppm)

6-amino-4-(2,5-dimethylphenyl)-3-methyl-1,4-dihydropyrano[2,3-*c*]pyrazole-5-carbonitrile (10ab)

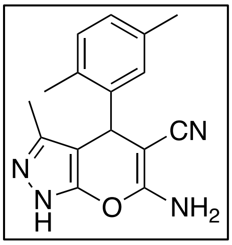

UPLC-UV: Rt = 1.46;
UPLC-MS (ESI) calculated for $C_{16}H_{17}N_4O$ [M + H]$^-$: *m/z* = 281, found *m/z* = 281.1;
^{1}H NMR (400 MHz, DMSO-d_6): δ = 12.05 (s, 1H, ArN**H**), 7.01 (d, *J* = 7.7 Hz, 1H, Ar**H**), 6.92 (dd, *J* = 7.8, 1.8 Hz, 1H, Ar**H**), 6.81 (s, 2H, ArN**H**$_2$), 6.78 (s, 1H, Ar**H**), 4.79 (s, 1H, C**H**Ar), 2.24 (s, 3H ArC**H**$_3$), 2.19 (s, 3H ArC**H**$_3$), 1.69 (s, 3H ArC**H**$_3$);
^{13}C NMR (100 MHz, DMSO-d_6): δ = 160.71, 155.05, 135.30, 135.01, 131.86, 130.40, 129.19, 127.31 (two overlapping signals), 120.72, 97.59, 56.87, 20.67 (two overlapping signals), 18.52, 9.56.

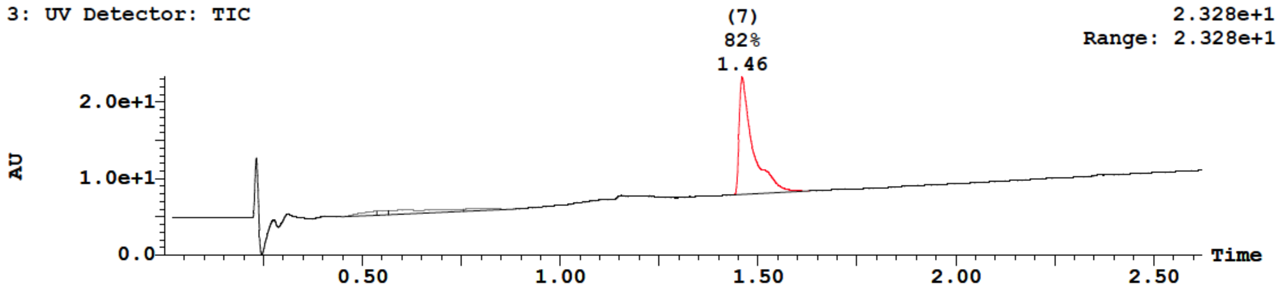

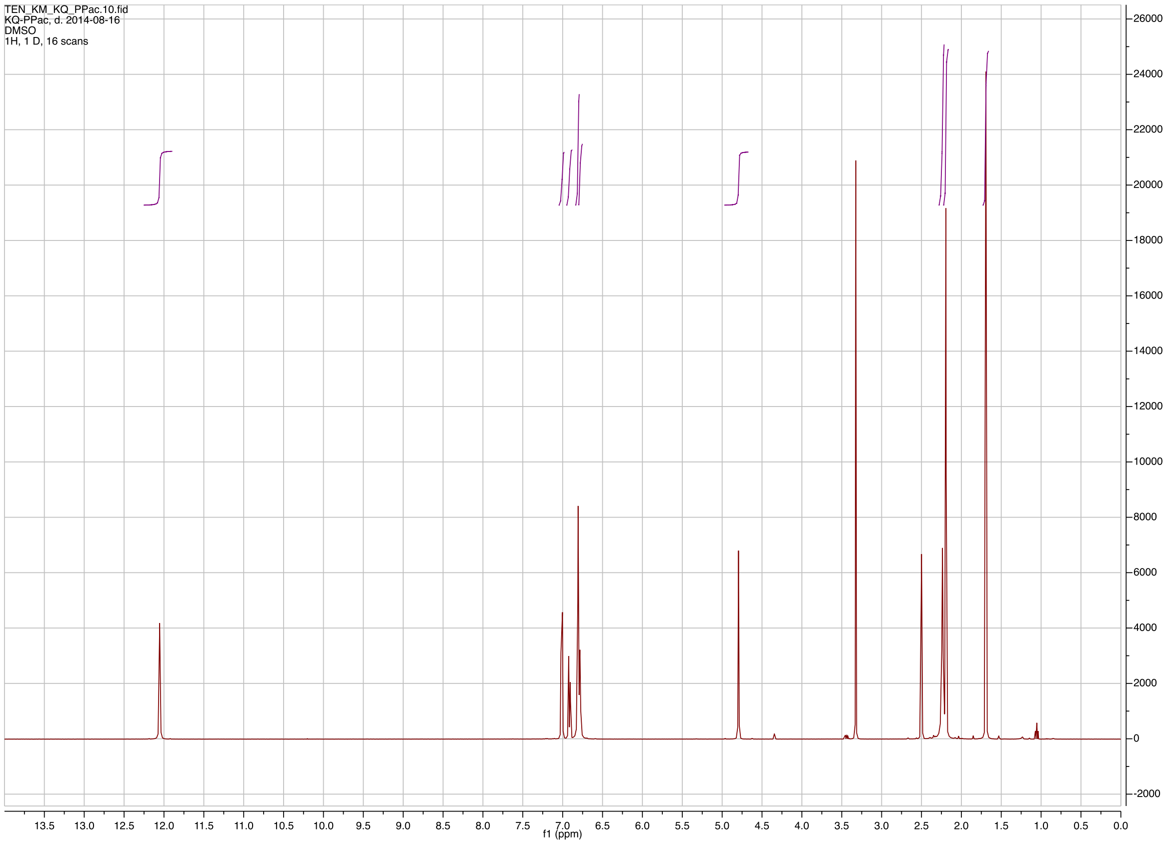

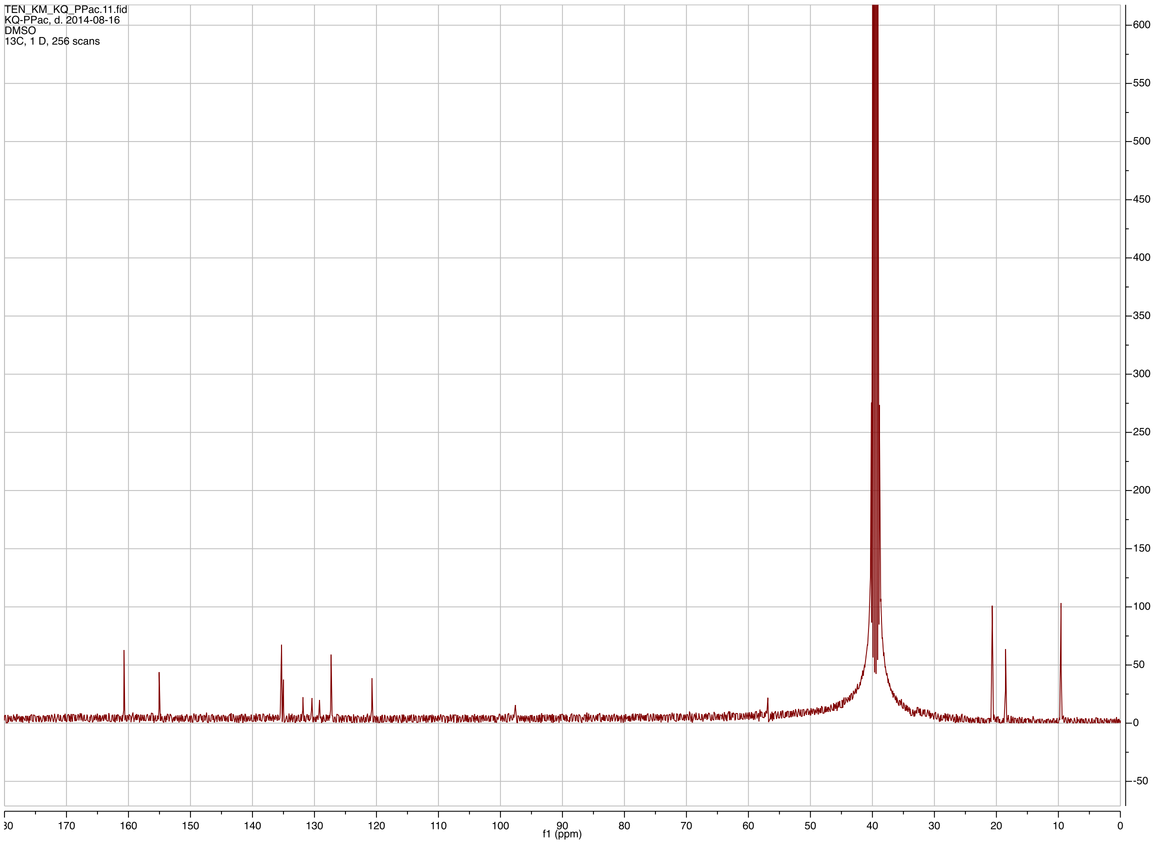

TEN_KM_KQ_PPac.11.fid
KQ-PPac, d. 2014-08-16
DMSO
13C, 1 D, 256 scans
f1 (ppm)

6-amino-4-(4-fluorophenyl)-3-methyl-1,4-dihydropyrano[2,3-*c*]pyrazole-5-carbonitrile (10ac)

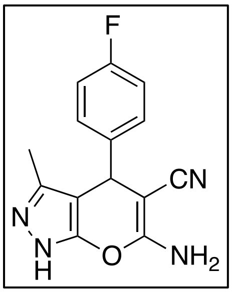

UPLC-UV: Rt = 1.25;
UPLC-MS (ESI) calculated for $C_{14}H_{12}FN_4O$ [M + H]$^-$: *m/z* = 271.1, found *m/z* = 271.1
^{1}H-NMR (400 MHz, DMSO-d_6): δ = 12.11 (s, 1H, ArN**H**), 7.29 – 7.03 (m, 4H, 4 × Ar**H**), 6.89 (s, 2H, ArN$\mathbf{H_2}$), 4.63 (s, 1H, C**H**Ar), 1.78 (s, 3H ArC$\mathbf{H_3}$);
^{13}C NMR (100 MHz, DMSO-d_6): δ = 160.94 (d, *J* = 241 Hz), 160.81, 154.69, 140.66 (d, *J* = 2.9 Hz), 135.61, 129.33 (d, *J* = 8.2 Hz), 120.70, 115.16 (d, *J* = 21.4 Hz), 97.49, 57.07, 35.43, 9.73; IR (neat):1395, 1491, 1591, 2198, 3090, 3226.

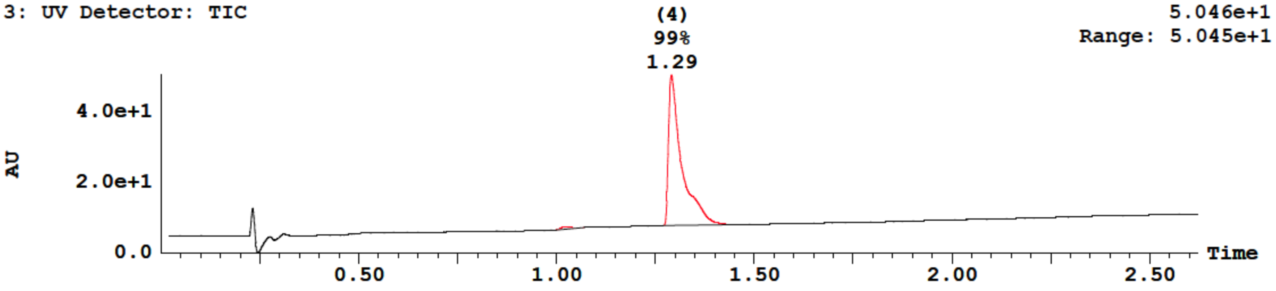

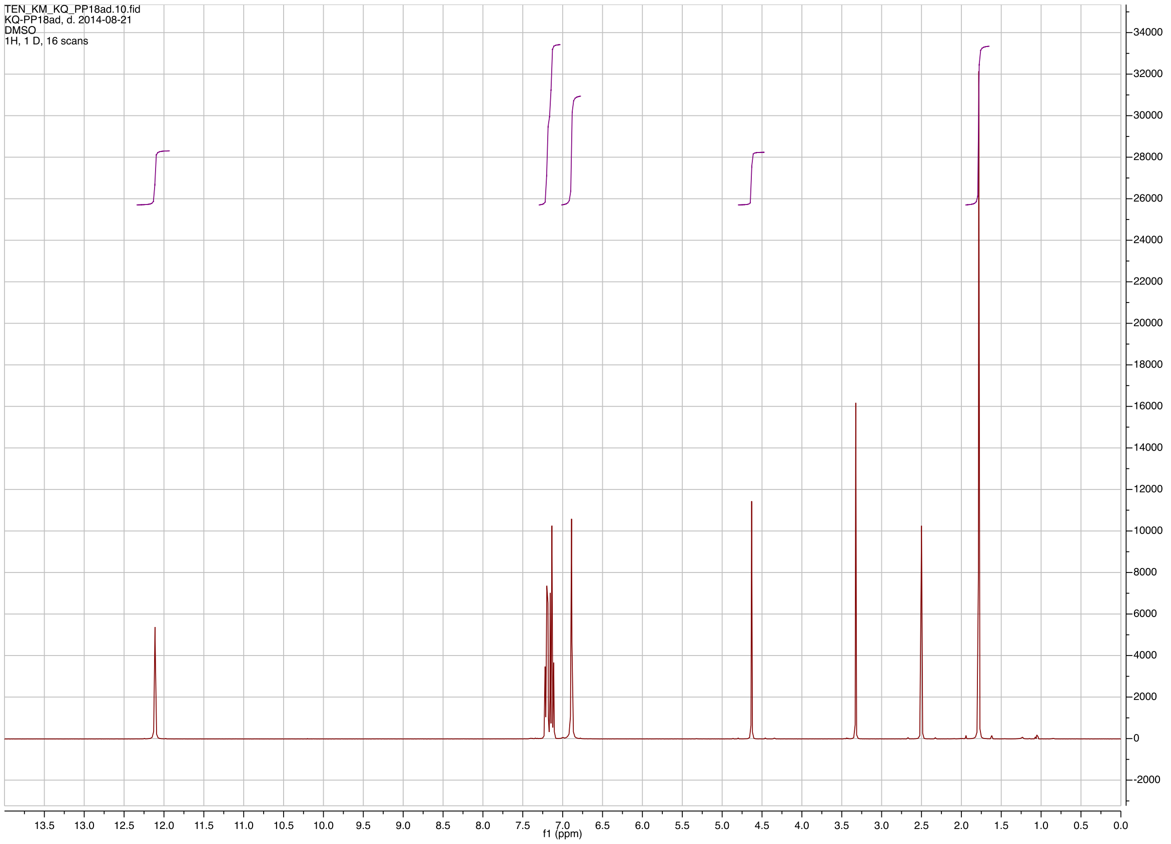

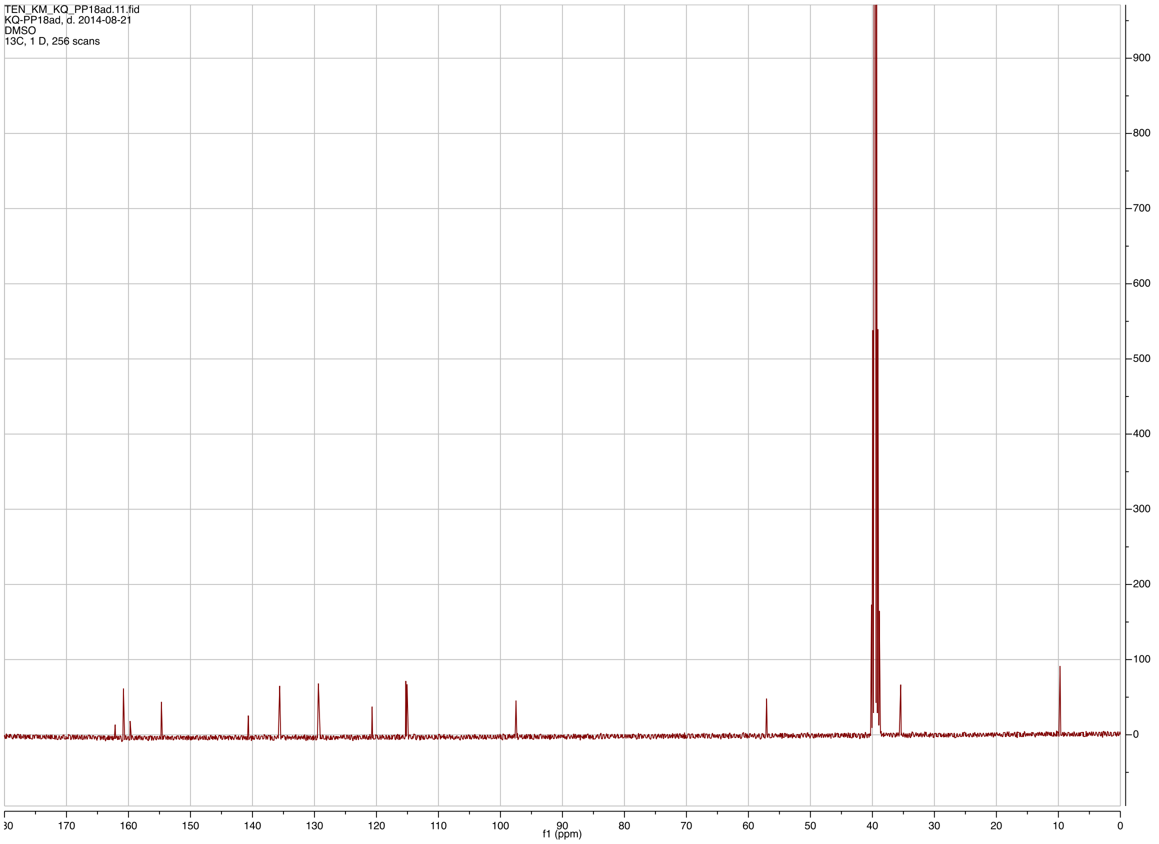
TEN_KM_KQ_PP18ad.11.fid
KQ-PP18ad, d. 2014-08-21
DMSO
13C, 1 D, 256 scans
f1 (ppm)
30
170
160
150
140
130
120
110
100
90
80
70
60
50
40
30
20
10
0
900
800
700
600
500
400
300
200
100
0

6-amino-3-methyl-1,4-diphenyl-1,4-dihydropyrano[2,3-*c*]pyrazole-5-carbonitrile (10ad)

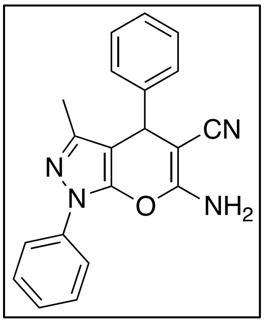

UPLC-UV: Rt = 1.91;
UPLC-MS (ESI) calculated for $C_{20}H_{17}N_4O$ $[M + H]^{-}$: *m/z* = 329.1, found *m/z* = 329.1;
^{1}H NMR (400 MHz, DMSO-d_6): δ = 7.82-7.75 (m, 2H, ArN**H**), 7.49 (dd, *J* = 8.5, 7.4 Hz, 2H, Ar**H**), 7.39-7.29 (m, 4H, Ar**H**), 7.29-7.23 (m, 4H, Ar**H**), 7.20 (s, 2H, ArN$\mathbf{H_2}$), 4.68 (s, 1H, C**H**Ar), 1.78 (s, 3H ArC$\mathbf{H_3}$);
^{13}C NMR (100 MHz, DMSO-d_6): δ = 159.41, 145.25, 143.87, 143.60, 137.52, 129.33, 128.52, 127.77, 127.04, 126.16, 120.00, 119.96, 98.63, 58.16, 36.73, 12.57.

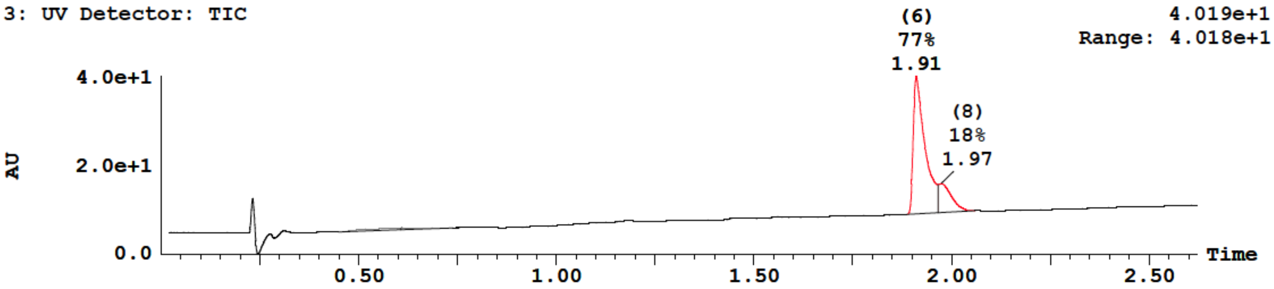

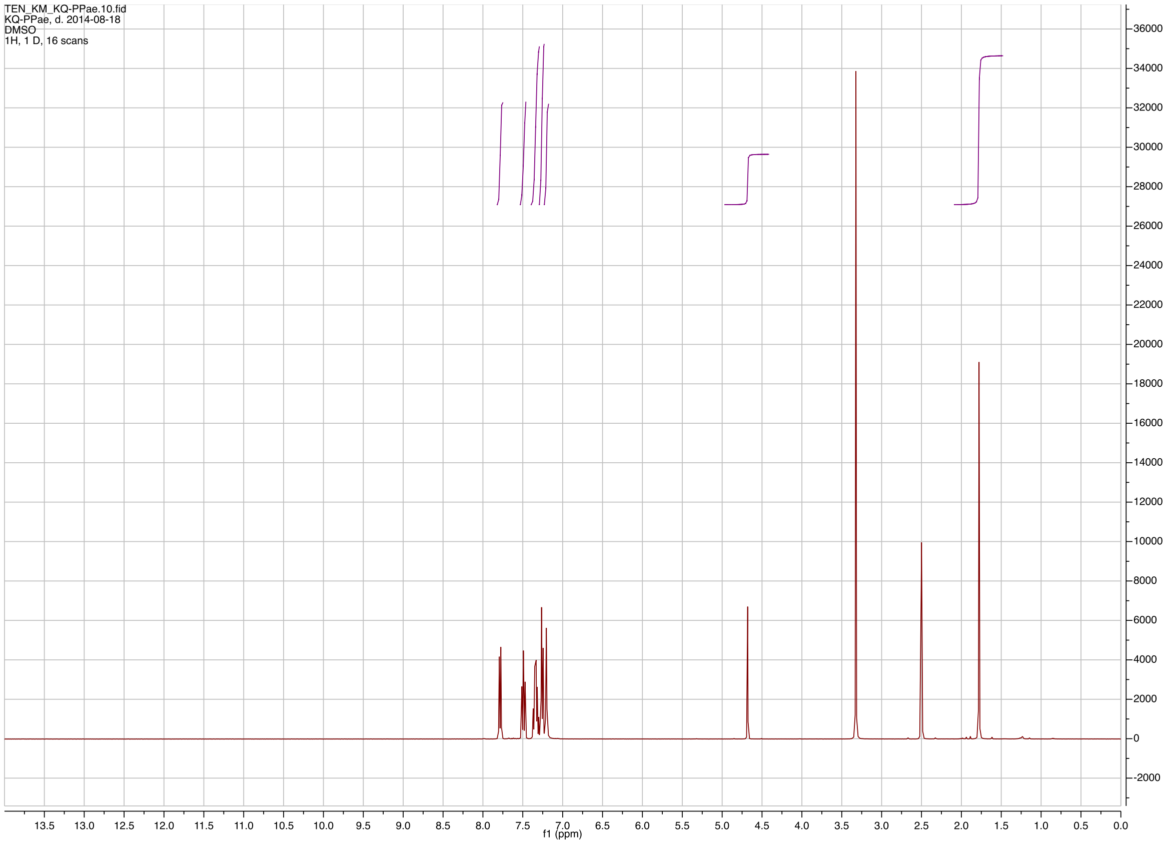

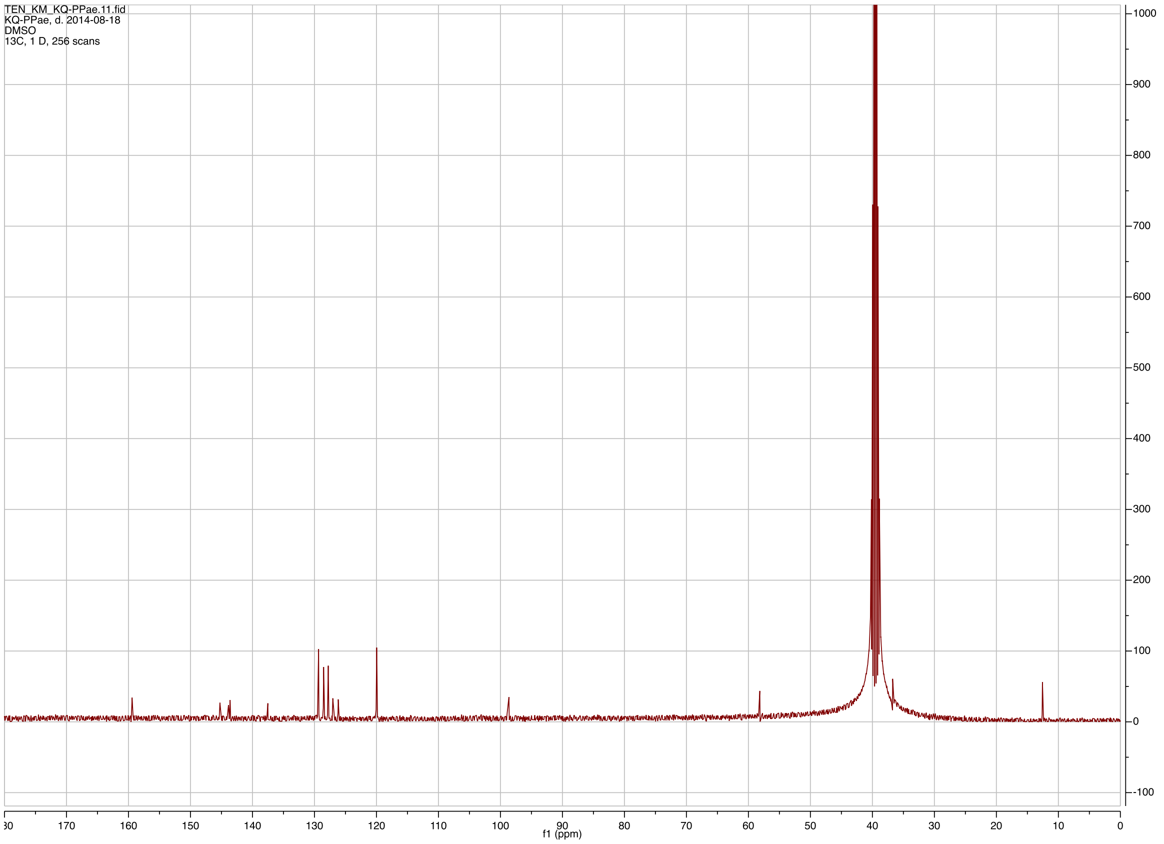
TEN_KM_KQ-PPae.11.fid
KQ-PPae, d. 2014-08-18
DMSO
13C, 1 D, 256 scans
f1 (ppm)

6-amino-1-(4-chlorophenyl)-4-(2,5-dimethylphenyl)-3-methyl-1,4-dihydropyrano[2,3-*c*]pyrazole-5-carbonitrile (10ae)

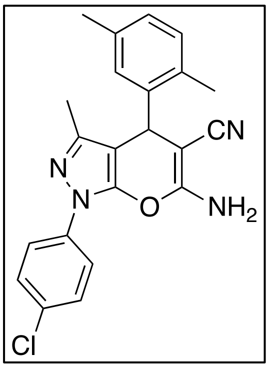

UPLC-UV: Rt = 2.32;
UPLC-MS (ESI) calculated for $C_{22}H_{20}ClN_4O$ $[M + H]^-$: *m/z* = 391.1 (^{35}Cl), 393.1 (^{37}Cl), found *m/z* = 391.2 (100 %), 393,2 (32 %);
^{1}H NMR (300 MHz, DMSO-d_6): δ = 7.84 (d, *J* = 8.9 Hz, 2H, ArNH), 7.53 (d, *J* = 8.9 Hz, 2H, ArH), 7.19 (s, 2H, ArNH_2), 7.06 (d, *J* = 7.7 Hz, 1H, ArH), 6.95 (dd, *J* = 7.7, 1.8 Hz, 1H, ArH), 6.87 (s, 1H, ArH), 4.90 (s, 1H, CHAr), 2.32 (s, 3H ArCH_3), 2.20 (s, 3H ArCH_3), 1.70 (s, 3H ArCH_3).

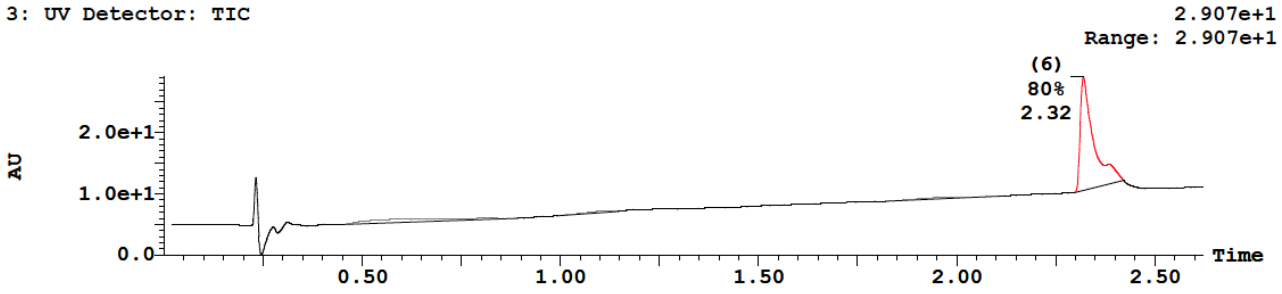

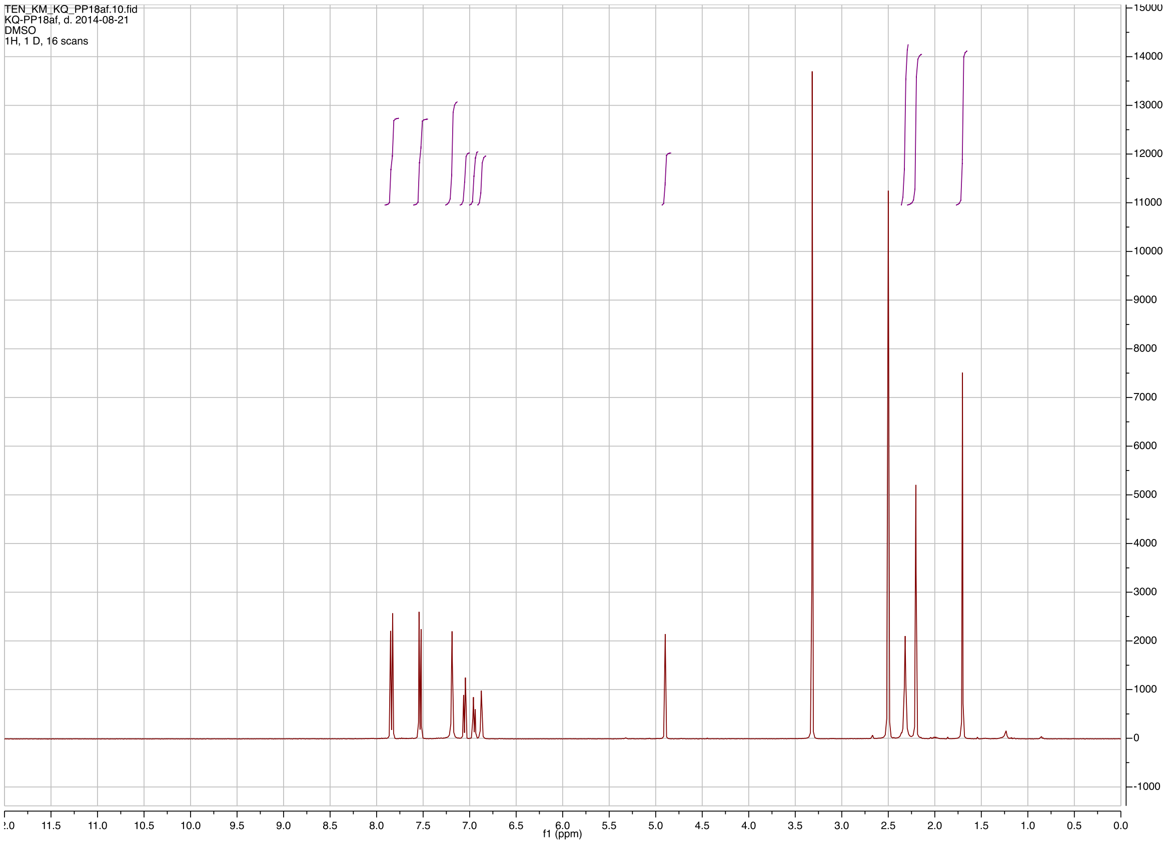

6-amino-1-(4-chlorophenyl)-3-methyl-4-(*o*-tolyl)-1,4-dihydropyrano[2,3-*c*]pyrazole-5-carbonitrile (10af)

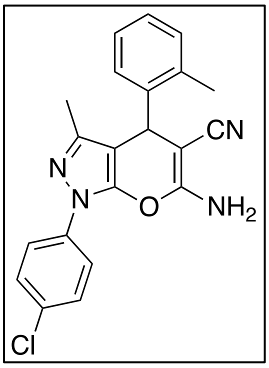

UPLC-UV: Rt = 2.21;
UPLC-MS (ESI) calculated for $C_{21}H_{18}ClN_4O$ $[M + H]^-$: *m/z* = 377.1 (^{35}Cl), 379.1 (^{37}Cl), found *m/z* = 377.1 (100 %), 379,1 (32 %);
^{1}H NMR (300 MHz, DMSO-*d*$_6$): δ = 7.83 (d, *J* = 7.6 Hz, 2H, 2 × Ar**H**), 7.53 (d, *J* = 7.7 Hz, 2H, 2 × Ar**H**), 7.22-7.09 (m, 6H, 4 × Ar**H** + ArN**H**$_2$), 4.94 (s, 1H, C**H**Ar), 2.37 (s, 3H ArC**H**$_3$), 1.69 (s, 3H ArC**H**$_3$);
IR (neat): 1384, 1511, 1662, 2224, 3192, 3332, 3453.

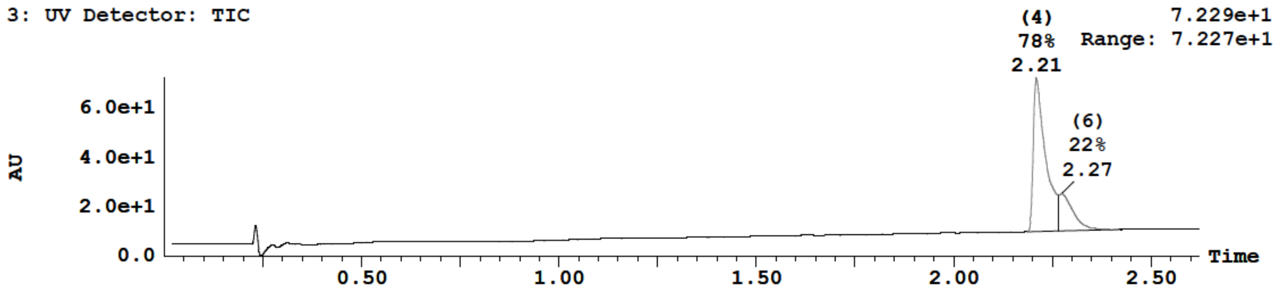

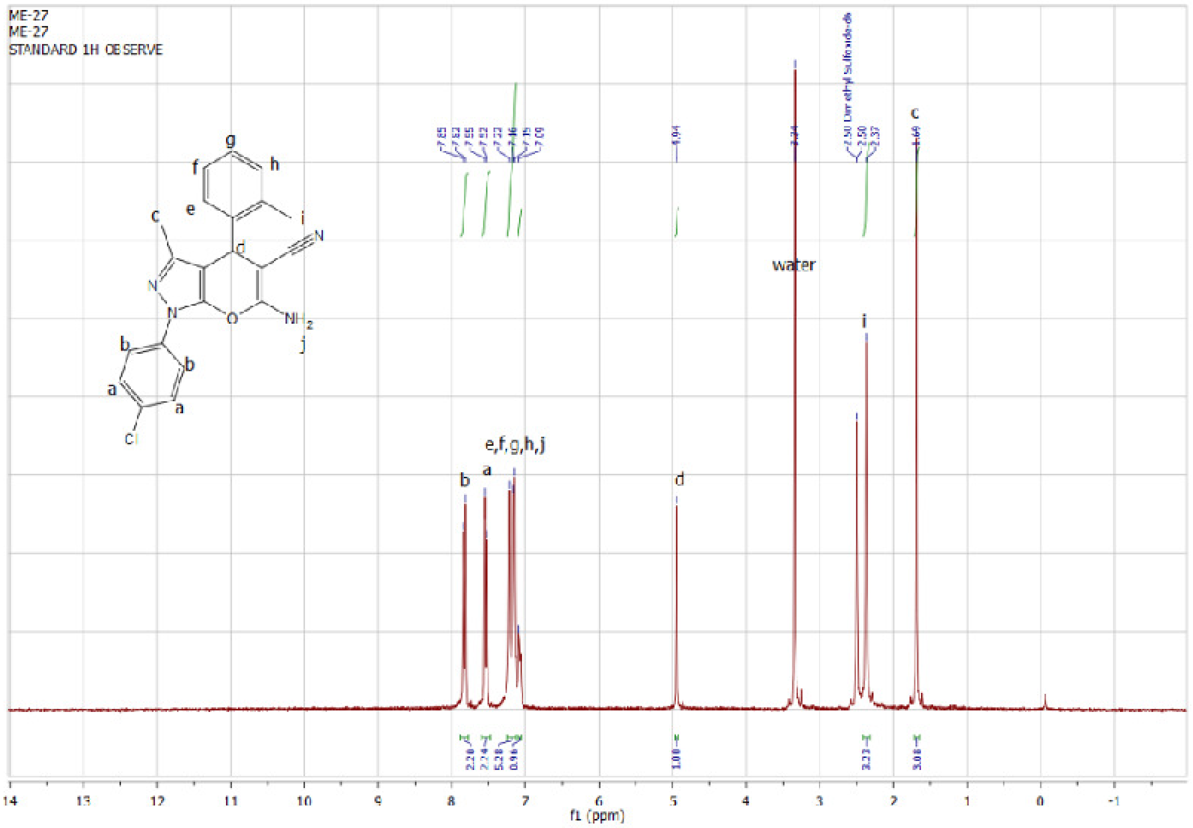

6-amino-4-(2,4-dichlorophenyl)-3-methyl-1-phenyl-2,4-dihydropyrano[2,3-*c*]pyrazole-5-carbonitrile (10ag)

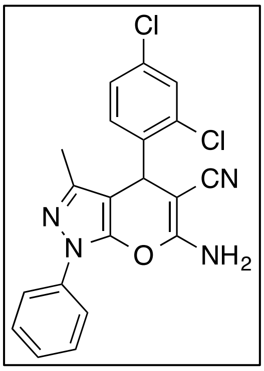

UPLC-UV: Rt = 2.38
UPLC-MS (ESI) calculated for $C_{20}H_{17}Cl_2N_4O$ $[M + H]^-$: *m/z* = 399.1 (2 × ^{35}Cl), 401.1 (1 × ^{37}Cl, 1 × ^{37}Cl), found *m/z* = 399.1 (100 %), 401,1 (64 %); ^{1}H NMR (300 MHz, DMSO-d_6): δ = 1.65 (s, 3H), 1.98 (s, 3H), 2.09 (s, 3H), 3.76 (s, 3H), 4.95 (s, 1H), 6.75-6.90 (m, 2H), 7.12 (s, 2H), 7.50 (d, 2H), 7.80 (d, 2H).

S2: Model set of compounds for development of binding mode model

S3: Test set of compounds for validation of binding mode model
