# Supplementary material for: Synthesis and biological evaluation of dihydropyrano-[2,3-c]pyrazoles as a new class of PPARγ partial agonists
Source: PLoS One. 2017 Feb 28;12(2):e0162642. doi: 10.1371/journal.pone.0162642 (PMC5330453; doi:10.1371/journal.pone.0162642)
Supplement: S2 File — (DOCX) [file pone.0162642.s002.docx]

**S2: Model set of compounds for development of binding mode model**

**S1 Table: Model set of compounds for development of binding mode model (continued).**
