# Supplementary material for: Synthesis and biological evaluation of dihydropyrano-[2,3-c]pyrazoles as a new class of PPARγ partial agonists
Source: PLoS One. 2017 Feb 28;12(2):e0162642. doi: 10.1371/journal.pone.0162642 (PMC5330453; doi:10.1371/journal.pone.0162642)
Supplement: S3 File — (DOCX) [file pone.0162642.s003.docx]

**S3: Test set of compounds for validation of binding mode model**

**S2 Table: Test set of compounds for validation of binding mode model (continued)**
